# Glutamine Deprivation Triggers Tribbles Homolog 3 Dependent G‐Quadruplex Resolution to Maintain DNA Repair and Tumor Survival

**DOI:** 10.1002/advs.202520798

**Published:** 2026-03-07

**Authors:** Qiang Ji, Xuedan Sun, Zhangran Sun, Mengfan Li, Xinyu Cheng, Shuai Tian, Rick F. Thorne, Jinming Li, Guangzhi Liu, Mian Wu, Xiaoying Liu

**Affiliations:** ^1^ Translational Research Institute of Henan Provincial People's Hospital and School of Basic Medical Sciences Henan University Zhengzhou China; ^2^ Department of Hepatobiliary Surgery Centre for Leading Medicine and Advanced Technologies of IHM The First Affiliated Hospital of USTC Division of Life Sciences and Medicine University of Science and Technology of China Hefei China; ^3^ School of Life Sciences Anhui Medical University Hefei China; ^4^ Henan Key Laboratory of Stem cell Differentiation and Modification Henan Provincial People's Hospital Zhengzhou China

**Keywords:** DDX5, DNA repair, G4 DNA, nutrient stress, TRIB3

## Abstract

Glutamine is an essential amino acid for tumor survival, but therapies targeting glutamine metabolism have largely failed due to adaptive resistance mechanisms. Here, we identify the pseudokinase TRIB3 as a key mediator of the metabolic adaptation of hepatocellular carcinoma (HCC) cells to limiting glutamine availability. TRIB3 is upregulated under glutamine deprivation in a c‐Jun‐dependent manner, functioning in the nucleus to safeguard DNA repair fidelity, allowing the timely resolution of DNA damage and preventing replication catastrophe. TRIB3 binds to G‐quadruplex DNA (G4‐DNA) structures throughout the genome, recruiting the helicase DDX5 to resolve them as a cooperative functional complex. Depleting TRIB3 or DDX5 in HCC cells leads to exaggerated G4‐DNA accumulation and heightened DNA damage associated with the downregulation of DNA damage repair (DDR) pathways. We illustrate this effect on homologous recombination (HR) pathway genes, finding that TRIB3‐DDX5 prevents G4‐DNA accumulation at the *BRCA1* and *RAD51AP1* promoter regions that would otherwise suppress transcription. In vivo, TRIB3 silencing suppresses HCC xenograft growth, patently increasing DNA damage and apoptosis when mice were maintained on glutamine‐deficient diets. Clinically, TRIB3 is overexpressed in HCC and correlates with poor prognosis. Our results propose the TRIB3‐DDX5‐G4 axis as a therapeutic target in HCC and other TRIB3‐high malignancies.

## Graphical abstract

Glutamine deprivation triggers transient DNA damage yet activates adaptive repair in hepatocellular carcinoma cells. We identify TRIB3 as a stress‐induced nuclear scaffold that associates with DDX5 and G‐quadruplex DNA at*BRCA1* and*RAD51AP1* promoters. TRIB3 loss increases G4 accumulation, suppresses HR gene transcription, elevates γ‐H2A.X, and sensitizes tumors to nutrient stress.

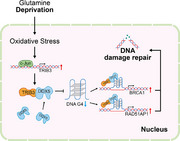

## 1 Introduction

Hepatocarcinoma consists of two major types: hepatocellular carcinoma (HCC) and intrahepatic cholangiocarcinoma (ICC), with the former accounting for over 80% of global liver cancer cases [1]. Treatment options for early‐stage disease include surgery, radiofrequency ablation, transarterial chemoembolization, and liver transplantation. However, most HCC cases are diagnosed at advanced stages, rendering these treatments ineffective.

Metabolic reprogramming enables cancer cells to overcome nutrient scarcity, hypoxia, and other metabolic challenges within the tumor microenvironment (TME) [2]. Glutamine (Gln), the most abundant circulating amino acid [3], is often indispensable for tumor survival. This dependency is reflected in the phenomenon of “glutamine addiction”, a phenotype characterized by increased glutaminase activity, dysregulated uptake, and reliance on Gln‐derived metabolites. Yet tumors face Gln shortages due to extreme demand and dysfunctional vasculature [4, 5]. To sustain proliferation, Gln‐addicted cells activate salvage pathways including autophagy [6], Gln‐independent anaplerosis [7, 8], macropinocytosis‐mediated amino acid scavenging, and fatty acid/lactate utilization [9, 10]. While these adaptations allow cancer cells to survive Gln fluctuations, they simultaneously create metabolic dependencies that can be therapeutically exploited—for example, by targeting compensatory pathways that become essential under nutrient stress.

As a central metabolic substrate, Gln contributes carbon to the TCA cycle for energy and nucleotide precursors while providing nitrogen for de novo biosynthesis [11], thereby supporting genome maintenance and efficient DNA replication. Critically, Gln also maintains redox homeostasis by serving as the primary precursor for the synthesis of the master antioxidant glutathione (GSH) while also fueling NADPH production to recycle oxidized GSH [11, 12]. States of Gln insufficiency can jeopardize genomic integrity by both impairing dNTP supplies and triggering oxidative DNA damage via the disabling of redox control. The resulting replicative stress manifests as cell cycle arrest leading to a dynamic interplay between salvage pathways seeking stress resolution or cell death induction [13]. Despite efforts to target this vulnerability through inhibitors of Gln uptake (e.g., V9302) or metabolism (e.g., CB‐839), these agents have shown limited efficacy in clinical trials [14, 15]. This resistance underscores our limited understanding of the dynamic adaptive mechanisms that drive metabolic resilience in Gln‐addicted tumors.

Of relevance to this study is the third member of the Tribbles pseudokinase family, Tribbles homolog 3 (TRIB3). Current evidence shows TRIB proteins function as molecular scaffolds, exerting effects through protein–protein interactions [16]. For example, TRIB3 physically interacts with PPARγ and suppresses its transcriptional activity [17], whereas TRIB3 stabilizes SSRP1 by coupling with USP10 to facilitate SSRP1 deubiquitination [18]. An initial characterization of TRIB3 in cancer (previously known as SKIP3) revealed it was liver‐restricted in normal tissues, with aberrant upregulation occurring in different carcinoma types including HCC [19, 20]. Moreover, functional studies in HCC cell models have implicated TRIB3 in sorafenib resistance, with recent findings that this resistance is triggered by ROS‐mediated ER stress and activation of the unfolded protein response (UPR) [21, 22]. Indeed, TRIB3 is commonly induced downstream of multiple stress‐response pathways including hypoxia [23], ER stress [24, 25], nutrient restriction (amino acid, glucose, and fatty acid deprivation) [26, 27, 28, 29, 30] and inflammation [31]. Acting as a molecular switch, TRIB3 orchestrates context‐dependent cell fate decisions by balancing adaptive survival and death pathways downstream of integrated stress signaling.

Here, we identify TRIB3 as a novel guardian of genome stability through its role in resolving Gln stress‐induced G‐quadruplex DNA (G4) structures. These planar nucleic acid motifs, formed through Hoogsteen hydrogen bonding between guanine bases, are abundant throughout the genome and serve important regulatory functions under physiological conditions [32, 33, 34, 35]. However, when pathologically stabilized, G4 structures can disrupt critical cellular processes, inducing DNA damage, interfering with essential protein–DNA interactions (including RNA polymerase binding), and impeding both transcription and replication [36, 37, 38]. We found that upregulated TRIB3 recruits the DEAD‐box helicase 5 (DDX5) to resolve G4 structures under Gln deprivation, while promoting transcriptional activation of DNA repair machinery, thereby ensuring the timely resolution of DNA damage and maintenance of genomic integrity. Disruption of this pathway under Gln‐deprived conditions exacerbates DNA damage and induces apoptosis in HCC cells, establishing a metabolic vulnerability that could be exploited in combination therapies with existing inhibitors of Gln uptake and metabolism.

## 2 Results

### 2.1 Gln Deprivation Triggers Acute DNA Damage but Elicits Adaptive Repair Responses in Cancer Cells

**FIGURE 1:**
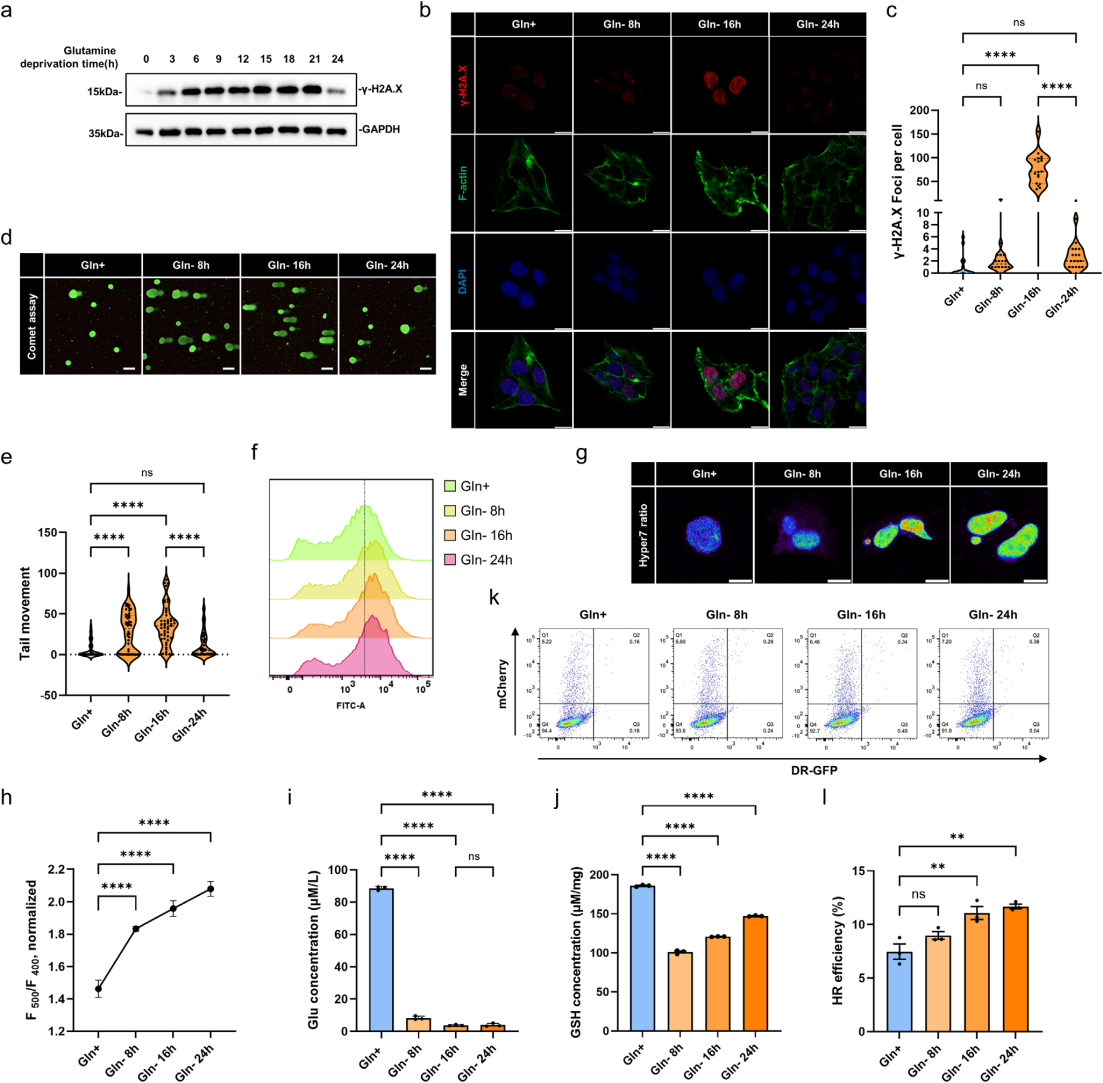
Glutamine (Gln) deprivation triggers DNA damage and modulates the DNA damage repair (DDR) in hepatocellular carcinoma (HCC) cells. (a) Expression of γ‐H2A.X (p‐Ser139) in HepG2 cells under Gln deprivation (0–24 h), as determined by Western blotting. (b, c) Representative confocal images of γ‐H2A.X (p‐Ser139) (red), F‐actin (green) and DAPI (blue) staining in HepG2 cells under Gln deprivation (0, 8, 16, 24 h) (scale bars, 10 µm) (b); quantification of γ‐H2A.X foci/cell (c). (d, e) Representative alkaline comet assays undertaken in HepG2 cells under Gln‐deprived conditions (0, 8, 16, 24 h) (scale bars, 10 µm) (d); quantification of comet tail movement (e). (f) Reactive oxygen species (ROS) levels in HepG2 cells under Gln deprivation for 0, 8, 16, 24 h measured using the DCFH‐DA probe, as determined by flow cytometry. (g, h) Representative confocal images of ROS signals (Ex500/ Ex400) in HepG2 cells with NLS‐Hyper7 overexpression under Gln deprivation for 0, 8, 16, 24 h (scale bars, 10 µm) (g); quantification of NLS‐Hyper7 ratios (h). (i, j) Intracellular glutamate (Glu) (i) and GSH (j) levels in HepG2 cells under Gln‐deprived conditions (0, 8, 16, 24 h). (k, l) Representative flow cytometric scatter plots depicting GFP versus mCherry distributions in HepG2 cells co‐expressing I‐SceI, DR‐GFP and mCherry reporters following Gln deprivation for 0, 8, 16, and 24 h (k) and HR activity. (l) quantified from the relative populations of GFP‐positive versus mCherry‐positive cells. Data information: (c,d) values are median, first and third quartile; (h, i, j, l) values are mean ± SEM; (c) *n* = 18, (e) *n* = 50, (h) *n* = 12, (i, j, l) *n* = 3 biological replicates; (c, e, h, i, j, l) one‐way ANOVA with Tukey's multiple comparison test. ns, not significant, ^**^ *p* < 0.01, ^****^ *p* < 0.0001.

To define how Gln scarcity impacts genomic stability, we monitored temporal dynamics of DNA damage and repair in Gln‐addicted HepG2 HCC cells. Using Western blotting analysis of γ‐H2A.X (p‐Ser139) as a marker of DNA double‐strand breaks (DSBs), we observed increased DSB levels within 12–18 h of Gln withdrawal which subsequently declined at 24 h (Figure 1a). Consistent findings were obtained using immunofluorescence staining against γ‐H2A.X (Figure 1b,c), while alkaline comet assays showed that the pronounced DNA fragmentation illustrated by the increased frequency comet tails was largely resolved 24 h post‐Gln deprivation (Figure 1d,e). Together these results reflect a time‐resolved interplay between Gln scarcity and genome maintenance, where acute metabolic stress induces repairable DSBs.

Previous studies demonstrate that Gln deficiency induces oxidative stress and DNA damage [39]. To validate oxidative stress, we measured ROS levels in Gln‐deprived HepG2 cells by flow cytometry, observing sustained increases over 24 h (Figure 1f). Further live imaging of Gln‐deprived HepG2 cells transfected with the nuclear H_2_O_2_ probe HyPer7 [40] revealed a progressive increase in the HyPer7 excitation ratio (Ex500/Ex400) over 24 h, indicating sustained nuclear H_2_O_2_ accumulation (Figure 1g,h). Notably, the persistent oxidative stress contrasts with the efficient DNA damage repair (DDR) observed by 24 h. Since Gln serves as a crucial precursor for GSH synthesis, we monitored this key antioxidant and its biosynthetic precursor. Intracellular levels of glutamate (Glu), the direct substrate for de novo GSH synthesis, dropped sharply and were nearly exhausted by ∼8 h of Gln deprivation (Figure 1i), confirming substrate insufficiency. Despite this, GSH levels declined more gradually, decreasing by less than 50% over 24 h (Figure 1j). This partial maintenance likely reflects the buffering capacity of the pre‐existing GSH pool and recycling of oxidized GSH via GSH reductase, which can temporarily sustain GSH levels even when de novo synthesis is limited. While partial recovery of GSH was observed by 24 h, this limited restoration—constrained by Glu depletion—was insufficient to effectively counteract the persistent accumulation of ROS. Nevertheless, DNA repair mechanisms were actively engaged during this period, as demonstrated by increased homologous recombination (HR) activity (Figure 1k,l).

Together these results propose that Gln‐deprived HepG2 cells adapt to chronic oxidative stress through partial GSH recovery and ROS‐independent DNA repair activation, revealing a prioritized genome stabilization response. This paradoxical adaptation prompted us to investigate the molecular mediators orchestrating these effects.

### 2.2 TRIB3 is Rapidly Upregulated and Nuclear‐Enriched in Response to Gln Deprivation and Correlates With Poor Clinical Outcomes

To identify mediators of the Gln deprivation response, we performed RNA‐seq on HepG2 cells at 12 and 24 h, integrating these data with a prior 48‐h dataset to identify 61 consistently differentially expressed genes (DEGs) across all timepoints (Figure S1a) [41]. Reactome pathway enrichment analysis of the 61 consistently DEGs revealed significant enrichment for pathways related to cellular responses to stress, stimuli, and starvation, as well as the UPR (Figure S1b).

**FIGURE 2:**
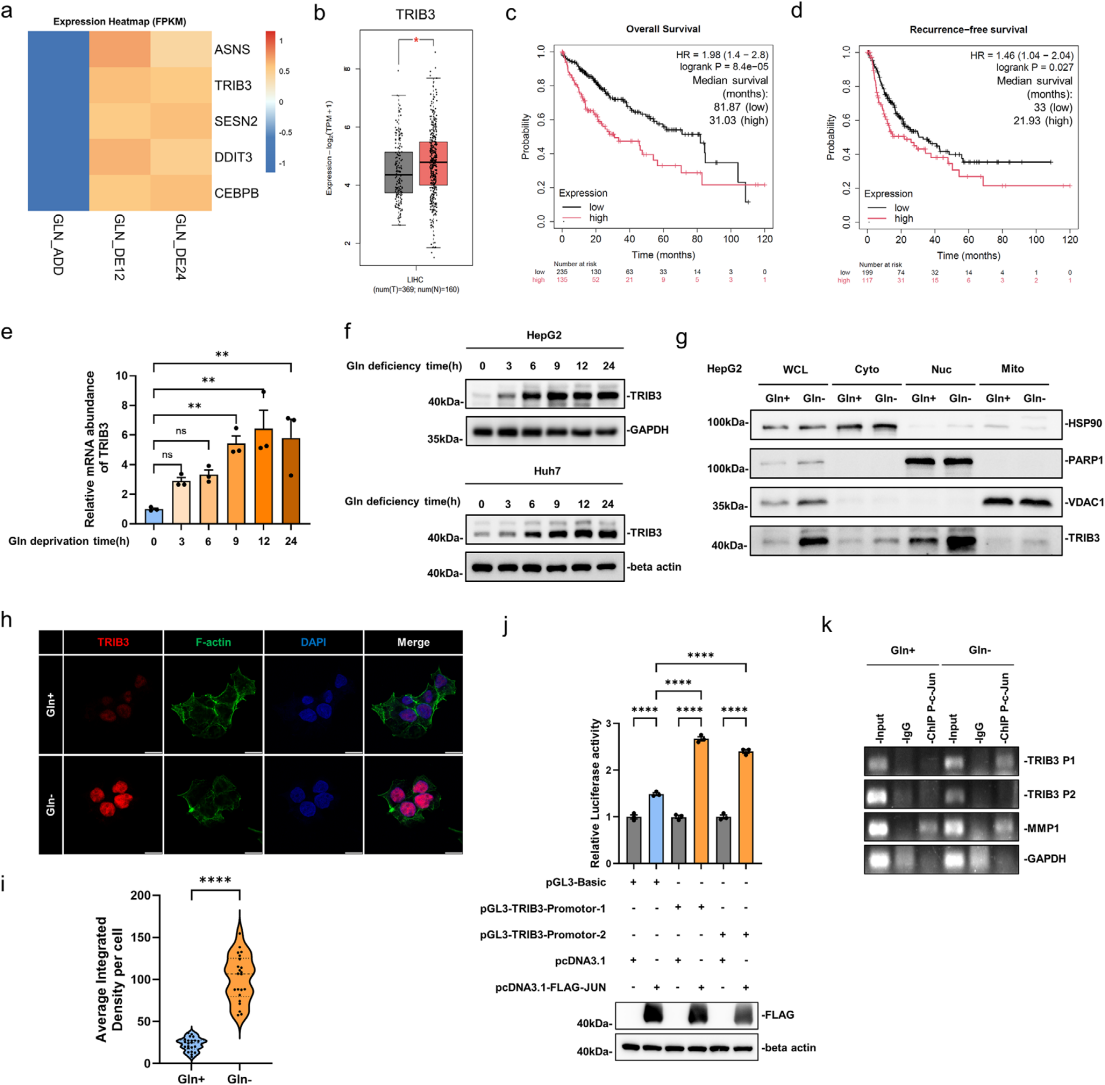
TRIB3 is upregulated and nuclea‐enriched in HepG2 cells under glutamine (Gln) deprivation. (a) Heatmap comparing the transcript expression of the ASNS, TRIB3, SESN2, DDIT3, and CEBPB genes in HepG2 cells cultured with Gln added (GLN_ADD) or withdrawn for 12 (GLN_DE12) or 24 h (GLN_DE24), based on RNA‐seq analysis. (b) TRIB3 expression (FPKM) in the TCGA liver hepatocellular carcinoma (LIHC) dataset in tumor (T) and normal (N) tissues, plotted with GEPIA2 (http://gepia.cancer‐pku.cn/). (c, d) Kaplan–Meier analysis of overall survival (OS; c) and recurrence‐free survival (RFS; d) in HCC patients stratified by TRIB3 mRNA expression, plotted using Kaplan–Meier Plotter (https://kmplot.com). Numbers at risk are indicated below each plot. (e) TRIB3 mRNA expression in HepG2 cells subjected to Gln deprivation for 0–24 h, determined by qPCR. (f) TRIB3 protein expression in HepG2 and Huh7 cells following Gln deprivation for 0–24 h, determined by Western blotting. (g) TRIB3 expression in whole cell lysates (WCL) and subcellular fractions (Cyto: cytoplasm; Nuc: nucleus; Mt: mitochondria) isolated from HepG2 cells with or without Gln deprivation for 24 h, determined by Western blotting. (h, i) Representative confocal images of γ‐H2A.X (p‐Ser139) (red), F‐actin (green), and DAPI (blue) staining in HepG2 cells with or without Gln deprivation for 12 h (scale bars, 10 µm) (h) and quantification of TRIB3 expression per cell (average integrated density) (i). (j) Dual‐luciferase reporter assays undertaken in HEK 293T cells after expression of the indicated pGL3 promoter constructs, with and without c‐Jun overexpression. (k) Endogenous ChIP analysis of p‐c‐Jun (Ser73) to the P1 or P2 region of the *TRIB3* promoter in HepG2 cells with or without 6 h of Gln deprivation, MMP1 served as a positive control. Data information: (c, d) *n* = 371 patients. (i) *n* = 22, (j) *n* = 3 biological replicates. (b, i) values are median, first and third quartile; (e) values are mean ± SEM; *n* = 3 biological replicates; (e, j) one‐way ANOVA with Tukey's multiple comparison test; (i) two‐tailed unpaired *t* test. ns, not significant, ^*^ *p* < 0.05, ^**^ *p* < 0.01, ^****^ *p* < 0.0001.

We focused on the five DEGs retrieved in the “Cellular response to starvation” pathway, namely ASNS, TRIB3, DDIT3, SESN2, and CEBPB, finding that TRIB3, DDIT3, and SESN2 were upregulated, CEBPB was downregulated, with ASNS being unchanged in response to Gln withdrawal (Figure 2a). Among these genes, only TRIB3 was significantly overexpressed in liver hepatocellular carcinoma (LIHC) compared to normal liver tissue (Figures 2b, S1c). Moreover, Kaplan–Meier survival analyses showed patients with higher tumor TRIB3 expression had a median overall survival of 31.0 months compared to 81.9 months for lower expression cases (HR = 1.98, 95% CI 1.4–2.8, *p* = 8.4 × 10^−^^5^) and median recurrence‐free survival (RFS) of 21.9 months versus 33.0 months (HR = 1.46, 95% CI 1.04–2.04, *p* = 0.027) (Figure 2c,d). Similarly, higher ASNS and DDIT3 were associated with worse OS whereas higher SESN2 or CEBPB expression levels predicted improved outcomes (Figure S1d–g). Among these stress‐responsive genes, TRIB3 was prioritized for further investigation based on its significant overexpression in LIHC and the strongest association with poor survival outcomes.

Tracking the kinetics of TRIB3 upregulation in HepG2 cells following Gln deprivation showed that its mRNA levels increased within 3 h, peaking at approximately sixfold after 12 h of deprivation (Figure 2e). These findings were reflected at the protein level where a robust and sustained increase in TRIB3 was observed in HepG2 and Huh7 cells (Figure 2f). Additionally, since TRIB proteins are known to exhibit dynamic and context‐dependent localization, we considered the site(s) of cellular localization of TRIB3 following Gln deprivation. Subcellular fractionation combined with immunofluorescence staining showed that TRIB3 is predominantly localized to the nucleus following Gln withdrawal (Figure 2g–i).

These results demonstrate that Gln deprivation triggers rapid nuclear accumulation of TRIB3 in HCC cells, with clinical data implicating its overexpression in poor HCC prognosis.

### 2.3 C‐Jun Drives Transcriptional Upregulation of TRIB3 During Gln Deprivation

Since TRIB3 upregulation is predominantly a transcriptional response, we used the integrative transcription factor target finder platform [42] to identify potential transcriptional regulators. The common outputs from six predictive algorithms identified three candidate transcription factors (Figure S2a) with knockdown experiments showing that JUN silencing but not MYC nor TFAP2C, markedly downregulated TRIB3 expression under basal conditions (Figure S2b). Moreover, JUN knockdown effectively abolished TRIB3 upregulation under Gln starvation (Figure S2c,d).

To identify c‐Jun specific motifs in the *TRIB3* proximal promoter, interrogation of the JASPAR database identified two putative binding sites, P1 and P2 (Figure S2e). Analysis of publicly available ENCODE HepG2 ChIP‐seq data revealed minimal c‐Jun occupancy at the P1 and P2 regions under basal (Gln‐replete) conditions (Figure S2e), consistent with the low basal TRIB3 expression observed. However, luciferase reporter assays using pGL3 vectors containing individual c‐Jun binding sites showed that both P1 and P2 fragments were transcriptionally responsive to ectopic c‐Jun expression in experiments undertaken with either HEK 293T and HepG2 cells (Figures 2j, S2f). Notably, under Gln deprivation, endogenous ChIP assays demonstrated that c‐Jun preferentially binds to the *TRIB3* P1 promoter site (Figure 2k), indicating that c‐Jun recruitment to the *TRIB3* promoter is stress‐inducible rather than constitutive. These results establish that JUN‐mediated transcriptional activation of TRIB3 occurs through stress‐inducible binding to the P1 promoter element, rather than constitutive occupancy.

Lastly, consistent with established evidence that oxidative stress activates the c‐Jun signaling pathway [43], we found that H_2_O_2_ treatment activated this pathway in HepG2 cells, an effect blocked by the antioxidant N‐acetyl‐L‐cysteine (NAC) (Figure S2g). This oxidative challenge also increased γ‐H2A.X and TRIB3 levels, and the attenuation of both by NAC establishes that TRIB3 upregulation is ROS‐dependent. While acute H_2_O_2_ induced TRIB3, the magnitude of induction was less pronounced than under Gln deprivation, suggesting that additional Gln‐sensitive mechanisms likely cooperate with oxidative stress to achieve full TRIB3 upregulation.

Collectively, these results establish c‐Jun as the primary transcriptional activator responsible for TRIB3 induction following Gln deprivation in HCC cells.

### 2.4 TRIB3 is Required for Efficient DDR by Sustaining DDR Pathway Activation

**FIGURE 3:**
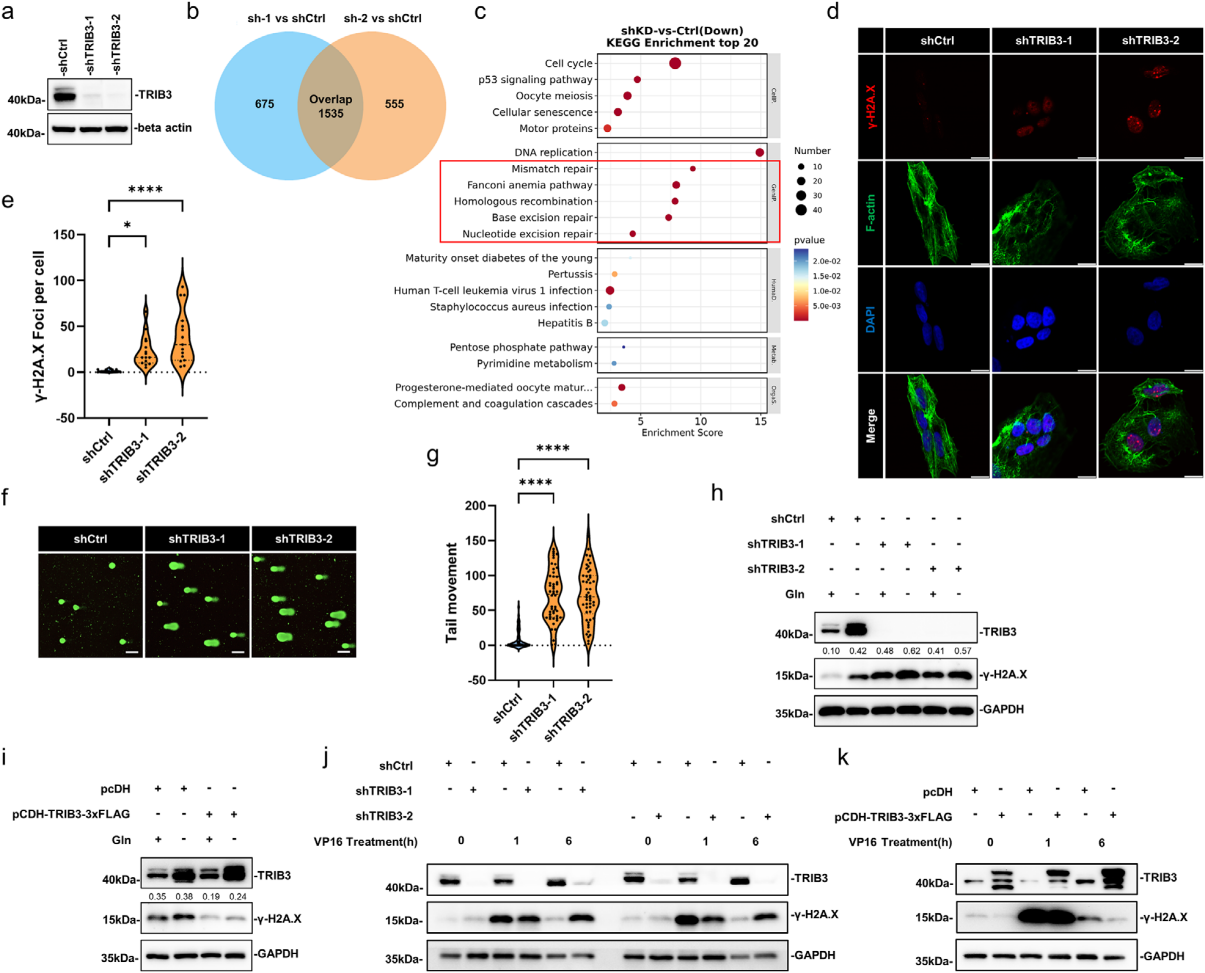
TRIB3 knockdown induces DNA damage and suppresses DNA damage repair (DDR) pathway. (a) Silencing efficiency of shRNA‐mediated knockdown of TRIB3 in HepG2 cells using independent shRNAs, confirmed by Western blotting. (b, c) Venn diagram intersection of differentially expressed genes (DEGs; |Log2FC|>1, *p* < 0.05) in HepG2 cells identified between control and TRIB3 shRNA knockdown conditions, as determined by RNA‐seq (b). Top 20 KEGG enrichment pathways obtained from the 1535 common genes identified in “sh‐1 vs shCtrl” and “sh‐2 vs shCtrl” comparisons (c). (d, e) Representative confocal images showing γ‐H2A.X (red), F‐actin (green), and DAPI (blue) staining in HepG2 cells following TRIB3 shRNA knockdown (scale bars, 10 µm) (d); quantification of γ‐H2A.X foci per cell (e). (f, g) Representative images of alkaline comet assay in HepG2 cells following TRIB3 shRNA knockdown (scale bars, 10 µm) (f); quantification of tail movement (g). (h, i) Western blotting analyses of HepG2 cells with and without TRIB3 knockdown (h) or transient TRIB3‐3xFLAG overexpression (i) against TRIB3, γ‐H2A.X, and GAPDH loading control. Numbers indicate relative band intensities for γ‐H2A.X normalized to GAPDH, as determined using Image Lab software (Bio‐Rad). (j, k) HepG2 cells with either TRIB3 knockdown (j) or overexpression (k) were treated for 1 h with 10 µM VP‐16 to induce double‐strand breaks (DSBs). Media were then replaced for an additional 1 or 6 h before harvest and Western blotting against TRIB3, γ‐H2A.X and GAPDH. Data information: (e, g) values are median, first, and third quartile; (e) *n* = 15, (g) *n* = 50; (e, g) one‐way ANOVA with Tukey's multiple comparison test. ^*^ *p* < 0.05, ^****^ *p* < 0.0001.

Building on the observation that HCC cells resolve DNA damage despite persistent oxidative stress under Gln deprivation (Figure 1), and having identified TRIB3 as a stress‐induced nuclear regulator (Figure 2), we next asked whether TRIB3 coordinates DDR. RNA‐seq analysis of TRIB3‐depleted HepG2 cells revealed broad downregulation of HR, mismatch repair (MMR), and excision repair pathways (Figures 3a–c, S3a–f), suggesting TRIB3 contributes to the activation of DDR programs. Consistent with persistent DNA damage, TRIB3 depletion increased γ‐H2A.X foci accumulation and DNA under basal conditions (Figure 3d–g). Moreover, TRIB3 knockdown exacerbated γ‐H2A.X levels under Gln starvation, whereas TRIB3 overexpression prevented the Gln depletion‐associated increase in γ‐H2A.X (Figure 3h,i), suggesting that TRIB3 promotes the resolution of DSBs. To assess this, we challenged cells with the DNA‐damaging agent etoposide (VP‐16) to rapidly induce DSBs. We observed that γ‐H2A.X levels were sustained in TRIB3 knockdown cells after 6 h of treatment whereas TRIB3 overexpression promoted a relative decrease in γ‐H2A.X levels compared to control cells (Figure 3j,k). These findings further associate TRIB3 with sustained regulation of DSB‐associated DDR following direct genotoxic challenge.

Together these results suggest that TRIB3 contributes to genomic integrity in HCC by supporting DDR processes in both metabolic and genotoxic stress contexts.

### 2.5 TRIB3 Forms a Nuclear Complex With DDX5 to Regulate DDR

**FIGURE 4:**
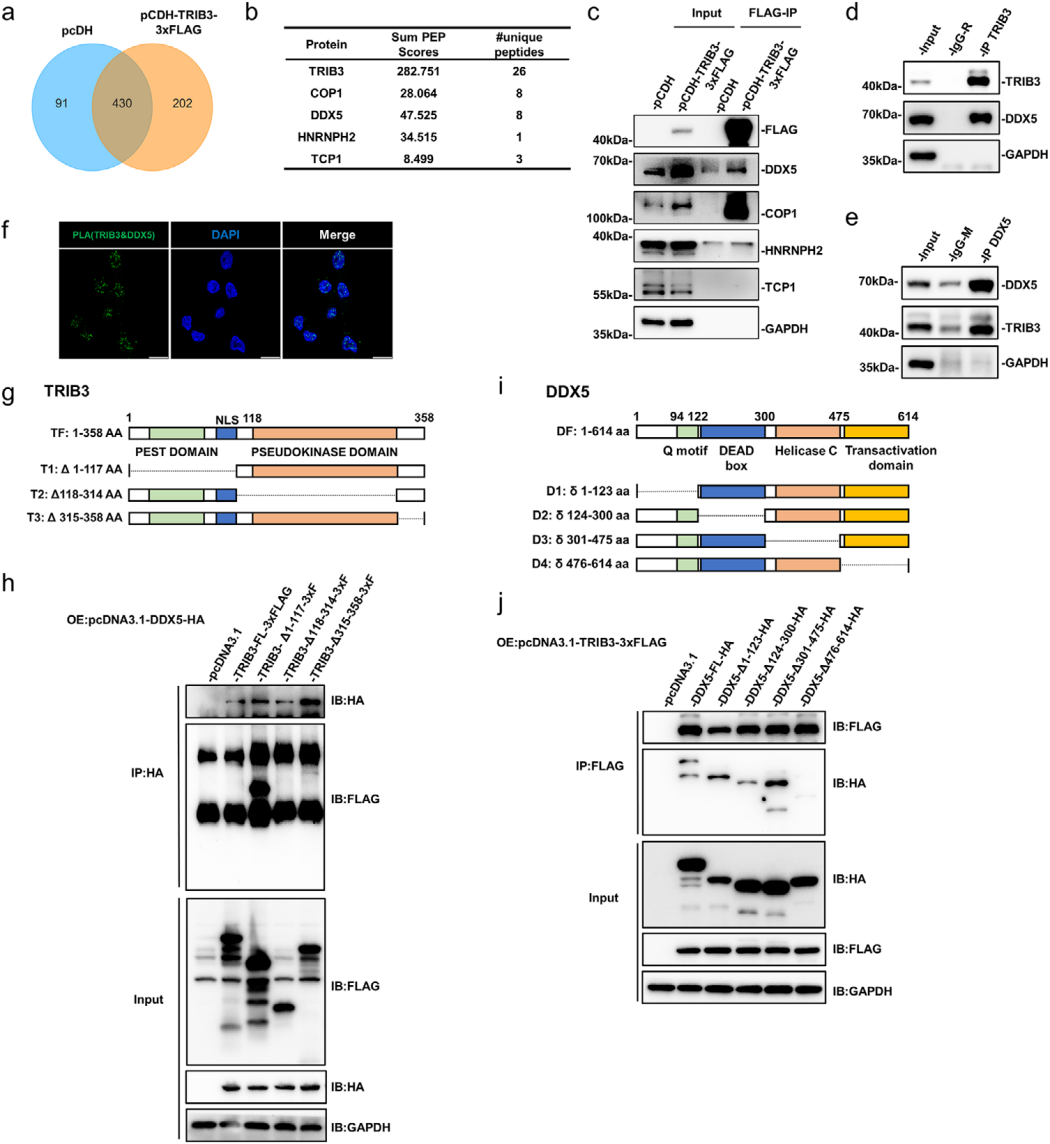
TRIB3 interacts with DDX5 in HepG2 cells. (a–c) Screening and verification of TRIB3‐interacting proteins. HepG2 cells bearing empty pCDH vector or exogenous pCDH‐TRIB3‐3×FLAG were subject to immunoprecipitation with FLAG magnetic beads before analysis by liquid chromatography‐mass spectrometry (LC‐MS). Venn diagram illustrates proteins recovered in each sample group (a); top 5 candidates recovered among the 202 specific TRIB3 interacting proteins (b); verification of the candidates in (b) by Western blotting (c). (d, e) Demonstration of reciprocal co‐immunoprecipitation between endogenous TRIB3 and DDX5 from HepG2 cells using antibodies against TRIB3 (d) or DDX5 (e). (f) Representative images of proximity ligation assay (PLA) in HepG2 cells showing interactions between TRIB3 and DDX5 (green), with nuclear counterstaining with DAPI (blue) (scale bars, 10 µm). (g, h) Schematic depicting the domain architecture of TRIB3 protein and the design of truncation mutants (g). HEK 293T cells were co‐transfected with pcDNA3.1‐DDX5‐HA in combination with full‐length (FL) FLAG‐tagged TRIB3 or truncation mutants before conducting immunoprecipitations against HA. Input lysates and immunoprecipitants were then subject Western blotting (h). (i, j) Schematic depicting the domain architecture of the DDX5 protein and the design of truncation mutants (i). Co‐transfection assay in HEK 293T cells using the pcDNA3.1‐TRIB3‐3×FLAG overexpression plasmid in combination with FL‐HA‐tagged DDX5 or DDX5 truncation mutants (j).

To uncover molecular partners underlying TRIB3's genome maintenance function, we performed affinity purification mass spectrometry (AP‐MS) against ectopically expressed FLAG‐TRIB3 in HepG2 cells. After eliminating artefacts, this analysis identified 202 candidate proteins including robust recovery of TRIB3 and the previously reported interacting protein COP1 (Figure 4a,b, and Table S1). Using the STRING database to construct a PPI network, we recovered a compact module of cellular components related to ubiquitin‐dependent protein degradation including the proteasome subunit alpha (PMSA) proteins (Figure S4a). TRIB3 formed part of a peripheral node closely connected to the DDX5 helicase and the RNA binding protein HNRNPH2 while the COP1 interaction formed an intermediary link to the proteasome module. Consistently, Gene Ontology (GO) enrichment analysis of the PPI network proteins recovered strong proteasome‐associated signatures along with hits on telomere maintenance and protein folding‐related functions (Figure S4b).

Together with COP1, we sought to verify candidate binding interactions between TRIB3, DDX5, and HNRNPH2, including TCP1 in the analysis, a protein specifically co‐purified with TRIB3 recovered with TRIB3, albeit with lower confidence. Immunoprecipitation (IP) assays targeting FLAG‐TRIB3 showed robust recovery of COP1, selective recovery of TRIB3 and to a lesser extent HNRNPH2, but no binding of TCP1 (Figure 4c). Experiments undertaken in the endogenous context showed that TRIB3 and DDX5 were reciprocally recovered in co‐IP experiments (Figure 4d,e). Moreover, proximity ligation assays (PLA) detected interactions between TRIB3 and DDX5, predominantly in the nucleus (Figure 4f) and domain mapping analyses showed that DDX5 binds to the pseudokinase domain of TRIB3 (aa 118–314) (Figure 4g,h), while TRIB3 associates with the C‐terminal transcriptional activation domain of DDX5 (aa 476–614) (Figure 4i,j). To further establish direct binding, we performed in vitro co‐IP using purified recombinant TRIB3‐His and DDX5‐FLAG proteins, which confirmed specific reciprocal interaction in a defined system free of cellular co‐factors (Figure S4c–f). Collectively, these data demonstrate that TRIB3 forms a specific nuclear complex with DDX5 through direct, reciprocal interactions between its pseudokinase domain and the C‐terminal transcriptional activation domain of DDX5.

If the TRIB3‐DDX5 interaction functionally contributes to the TRIB3‐mediated DNA damage response, we reasoned that knockdown of DDX5 should recapitulate the effects of TRIB3 silencing. Accordingly, targeted knockdown of DDX5 using independent shRNAs resulted in a marked increase in γ‐H2A.X levels, nuclear foci formation, and DNA fragmentation (Figure S5a–e). This phenocopy of the DDR observed upon TRIB3 depletion strongly suggests that DDX5 is not merely an interacting partner but an essential functional component of this complex. Nonetheless, how DDX5 contributes to the TRIB3‐mediated DDR remained an open question.

### 2.6 TRIB3 Promotes DDX5 Association With Genomic G4‐DNA

**FIGURE 5:**
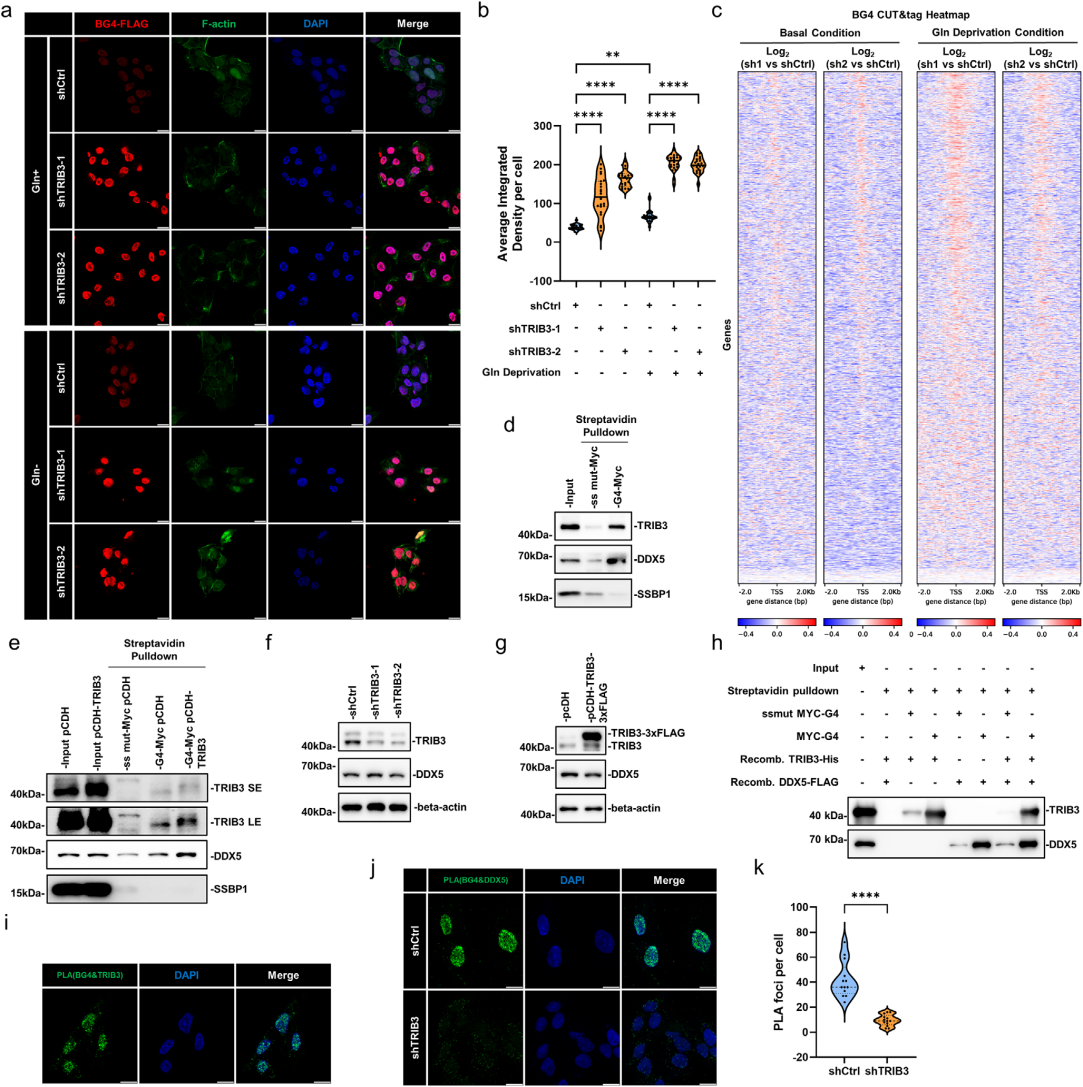
TRIB3 knockdown leads to accumulation of G4 which binds to the TRIB3/DDX5 complex. (a, b) Representative confocal images of BG4 (red), F‐actin (green), and DAPI (blue) staining in HepG2 cells following TRIB3 knockdown in combination with Gln deprivation for 12 h (scale bars, 10 µm) (a); quantification of average integrated density of BG4 per cell (b). (c) BG4 CUT&Tag signal heatmaps in log2 scale centered around genome‐wide TSS (±2 kb) comparing differences between shCtrl and shTRIB3 knockdown HepG2 cells. Averaged results from duplicate determinations using two independent shRNAs (shTRIB3‐1 and shTRIB3‐2) under normal (left) and Gln‐depleted conditions (right). Red signal signifies increased G4‐DNA occupancy of promoter regions. (d) Pulldown assay using streptavidin beads preloaded with biotinylated annealed G4‐Myc or ssmut‐Myc probes incubated with HepG2 nuclear lysates. Input and pulldown samples were subject to Western blotting against TRIB3, DDX5, and SSBP1 as a loading control. (e) Streptavidin‐based pulldown assays conducted as per (d) with the nuclear lysates of HepG2 cells without or with TRIB3 overexpression. Short (SE) and long (LE) exposures are shown for the TRIB3 Western blotting panel. (f, g) Western blotting analysis of TRIB3 and DDX5 in HepG2 cells subject to TRIB3 knockdown (f) or overexpression (g). (h) In vitro pulldown assays were conducted using purified recombinant TRIB3 and DDX5 proteins in combination with biotinylated annealed G4‐Myc or ssmut‐Myc probes as indicated. The probes were recovered with Streptavidin beads and the binding of TRIB3 and DDX5 monitored by Western blotting. (i) Proximity ligation assays (PLA) assays conducted between BG4 and TRIB3 in HepG2 cells. Representative image of PLA foci demonstrating BG4‐TRIB3 interactions (green), with nuclear staining for DAPI (blue) (scale bars, 10 µm). (j, k) PLA assays conducted between BG4 and DDX5 in HepG2 cells with or without TRIB3 knockdown. Representative PLA images (scale bars, 10 µm) (j); quantification of interaction foci per cell (k). Data information: (b, k) values are median, first and third quartile; (b) *n* = 23, (c) *n* = 2 technical replicates, (k) *n* = 20; (b) one‐way ANOVA with Tukey's multiple comparison test; (k) two‐tailed unpaired *t* test. ^**^ *p* < 0.01, ^****^ *p* < 0.0001.

Given that DDX5 is a known resolvase of G4 structures [44], and that unresolved G4s are established drivers of replication stress and genomic instability [37, 38], we hypothesized that the TRIB3‐DDX5 complex might suppress DNA damage by actively resolving G4‐DNA structures. To test this, we first assessed global G4 dynamics using the BG4 monoclonal antibody, a well‐validated tool for detecting in situ G4 structures when combined with epifluorescence microscopy [45]. Consistent with our hypothesis, knockdown of TRIB3 dramatically elevated nuclear G4 structures, an effect amplified by Gln deprivation (Figure 5a,b). Notably, DDX5 knockdown produced identical phenotypic outcomes to TRIB3 knockdown (Figure S6a,b), proposing that both proteins function within the same pathway to resolve G4 structures. To complement these observations, we performed CUT&Tag profiling of BG4 to examine G4‐DNA occupancy at genomic resolution. Analyses focused on transcription start site (TSS) regions showed that TRIB3 knockdown modestly increased G4‐DNA signal intensity under basal culture conditions, an effect that was further enhanced by Gln restriction (Figure 5c). These results corroborate the immunofluorescence data and support an association between TRIB3 loss and G4‐DNA accumulation at promoter‐proximal regions.

Next, to determine whether TRIB3 and/or DDX5 interact directly with G4 structures, we designed biotinylated Myc‐G4 probes‐a widely used benchmark G‐quadruplex structure‐along with Myc mutant control probes for use in vitro pulldown assays (Figure S6c). We found that endogenous TRIB3 and DDX5 in HepG2 cells were efficiently recovered with Myc‐G4 but not control probes (Figure 5d). Moreover, TRIB3 overexpression enhanced the recovery of DDX5 in the Myc‐G4 assay (Figure 5e), although this was not due to TRIB3 altering DDX5 levels as shown by TRIB3 knockdown and overexpression experiments (Figure 5f,g), suggesting that TRIB3 facilitates DDX5 recruitment to G4 structures. Indeed, in vitro binding assays with recombinant proteins (Figure S4c,d) showed that TRIB3 was robustly recovered by the Myc‐G4 probe (Figure 5h), establishing a direct, structure‐dependent interaction between TRIB3 and G4‐DNA.

To demonstrate this function in intact cells, we utilized PLA with the BG4 probe to assess binding between G4 DNA structures and TRIB3 or DDX5. Within cell nuclei, we readily observed PLA foci representing interactions between both G4 DNA, TRIB3, and DDX5, respectively, while TRIB3 knockdown markedly reduced the interaction foci between BG4 and DDX5 (Figure 5i–k). As further validation of the G4‐dependency of these interactions, we introduced a G4 decoy (thrombin‐binding aptamer, TBA) into HepG2 cells before repeating the PLA‐based experiments. While TBA transfection significantly reduced BG4‐DDX5 PLA signals it did not influence TRIB3‐DDX5 protein interactions (Figure S6d–g), demonstrating that endogenous TRIB3‐DDX5 complexes can be competitively displaced without dissociating the core protein interaction.

Collectively, these findings support a model in which TRIB3 functions as a G4‐binding scaffold that promotes the association of DDX5 with genomic G4 structures. The accumulation of G4‐DNA observed following loss of either TRIB3 or DDX5, and the enhancement of this phenotype under Gln‐restricted conditions, is consistent with their cooperative involvement in pathways that limit G4 persistence. The further observation that TRIB3 depletion is associated with altered G4 occupancy at promoter‐proximal regions prompted us to next examine the transcriptional consequences of impaired G4 regulation in TRIB3/DDX5‐deficient cells.

### 2.7 TRIB3‐DDX5 Prevents G4‐Driven Transcriptional Suppression of HR Genes

**FIGURE 6:**
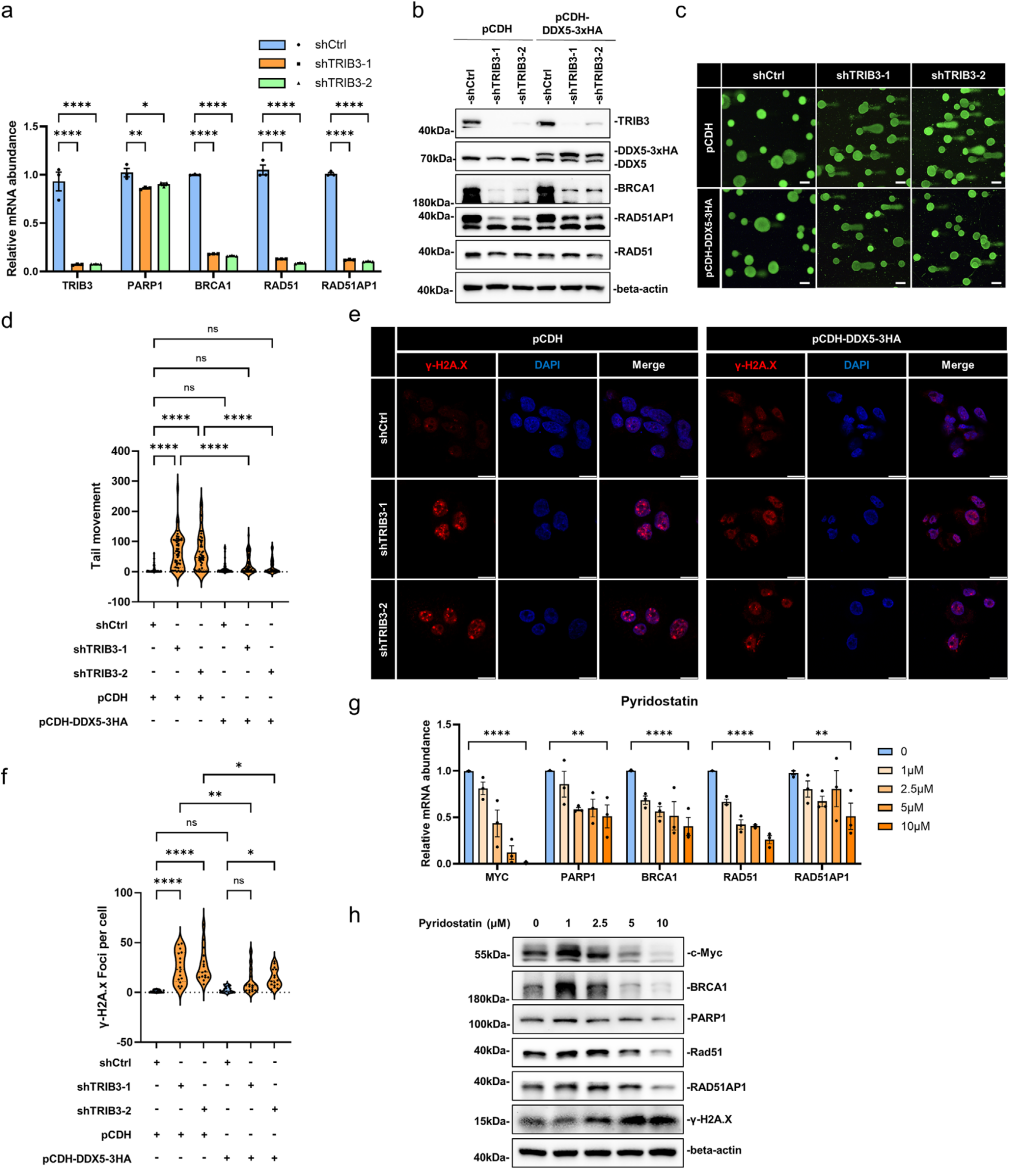
TRIB3 knockdown downregulates homologous recombination (HR) pathway genes. (a) Relative mRNA expression levels of TRIB3 and HR pathway‐related genes in HepG2 cells without and with TRIB3 knockdown, as determined by qPCR. (b) Western blotting of TRIB3 and HR pathway‐related genes in control or TRIB3 knockdown HepG2 cells in combination without (empty pCDH vector) or with exogenous DDX5 (pCDH‐DDX5‐3×HA) overexpression. (c, d) Representative images of alkaline comet assay in control or TRIB3 knockdown HepG2 cells in combination without or with DDX5 overexpression (scale bars, 10 µm) (c); quantification of tail movement (d). (e, f) Representative confocal images showing γ‐H2A.X (p‐Ser139) (red) and DAPI (blue) staining in control or TRIB3 knockdown HepG2 cells in combination without or with DDX5 overexpression (scale bars, 10 µm) (e); Quantification of γ‐H2A.X foci per cell (f). (g, h) HepG2 cells were treated with a concentration gradient of pyridostatin for 48 h. The mRNA and protein expression of HR‐related genes was assessed by qPCR (g) and Western blotting (h), respectively. MYC was included a positive control. Data information: (a, g) values are mean ± SEM, *n* = 3 biological replicates; (d, f) values are median, first and third quartile; (d) *n* = 50, (f) *n* = 16; (a, d, f, g) one‐way ANOVA with Tukey's multiple comparison test. ns, not significant, ^*^ *p* < 0.05, ^**^ *p* < 0.01, ^****^ *p* < 0.0001.

Having established that the TRIB3‐DDX5 complex interacts with G4 structures and correlates with their resolution, we next examined whether G4 accumulation in TRIB3‐ or DDX5‐depleted cells is associated with transcriptional changes in DNA repair pathways, as suggested by our RNA‐seq data (Figure S2a–c). We focused on HR given its reliance on sustained expression of key genes such as BRCA1 and RAD51AP1. Consistent with this, knockdown of either TRIB3 and DDX5 was accompanied by reduced expression of multiple HR components (BRCA1, RAD51AP1, PARP1, and RAD51) under standard culture conditions at both the mRNA and protein levels (Figures 6a, S7a–c). The suppressive effect of TRIB3 depletion on HR gene expression was also observed under Gln deprivation (Figure S7d), indicating that this association is not restricted to conditions of Gln limitation. Notably, DDX5 overexpression led to limited restoration of HR mediator protein levels in TRIB3‐knockdown cells (Figure 6b), suggesting that TRIB3 contributes to maintaining basal expression of these factors. In line with this, ectopic DDX5 expression attenuated, although not fully normalizing, increases in DNA fragmentation and γ‐H2A.X foci observed following TRIB3 depletion (Figure 6c–f), indicating incomplete functional rescue.

To further explore the link between G4 structures and HR gene expression, we treated cells with small‐molecule G4 stabilizers. Pyridostatin treatment recapitulated several features of TRIB3 or DDX5 knockdown [46, 47], including dose‐dependent reduction in HR gene expression and increased γ‐H2A.X accumulation (Figure 6g,h). Importantly, PhenDC3—a structurally distinct G4 ligand reported to exhibit fewer off‐target effects [48]—similarly reduced the expression of HR/DDR genes (Figure S7e), supporting an association between G4 stabilization and diminished repair gene transcription.

Collectively, these findings support a model in which the TRIB3‐DDX5 complex is associated with the maintenance of DDR gene expression, potentially through modulation of G4‐associated DNA structures.

### 2.8 TRIB3‐DDX5 is Associated with Promoter G4 Structures and HR Gene Expression

**FIGURE 7:**
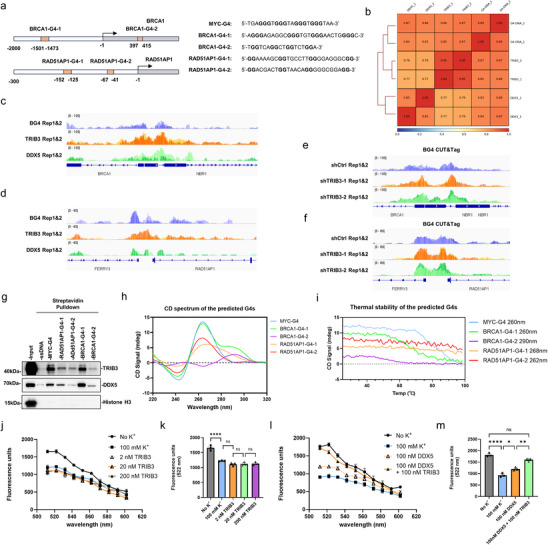
TRIB3‐DDX5 complex binds G4 structures in the promoters of *BRCA1* and *RAD51AP1*. (a) Schematic of the *BRCA1* and *RAD51AP1* proximal promoter regions depicting predicted G4 sequences. Experimentally validated sites obtained using the “Li+K+PDS‐treated” ChIP dataset from the G4Bank database ( https://tubic.tju.edu.cn/g4bank/ ) were ranked by QGRS mapper (https://bioinformatics.ramapo.edu/QGRS/index.php/) to identify high scoring sites. (b) Spearman correlation analysis of CUT&Tag peaks for BG4, TRIB3, and DDX5 in HepG2 cells. (c, d) IGV tracks across the BRCA1 (c) RAD51AP1 (d) loci depicting CUT&Tag peaks for BG4, TRIB3, and DDX5 from HepG2 cells under glutamine (Gln) deprived culture conditions (*n* = 2 technical replicates). Replicates were plotted using the same scale and differentially depicted by color shading. (e, f) IGV tracks across the BRCA1 (e) RAD51AP1 (f) loci depicting CUT&Tag peaks for BG4, TRIB3, and DDX5 from HepG2 cells subjected to control (shCtrl) or TRIB3 knockdown (shTRB3‐1, shTRIB3‐2) under Gln deprived conditions (*n* = 2 technical replicates). (g) Pulldown assay against HepG2 nuclear lysates using streptavidin beads preloaded with biotinylated annealed probes based on the G4‐DNA motifs in the *BRCA1* and *RAD51AP1* promoters in conjunction with ssDNA and MYC‐G4 control probes. Input and pulldown samples were subject to Western blotting against TRIB3, DDX5, and a histone H3 loading control. (h, i) Circular dichroism (CD) spectra (h) and thermal stability (i) of annealed G4‐DNA oligonucleotide probes used in the pulldown assays. (j‐m) Cell free G4‐DNA unwinding assays undertaken with 100 nM MYC‐G4‐Pu28 probes incubated with 2–200 nM TRIB3 (j) or with 100 nM DDX5 alone or in combination with 100 nM TRIB3 (l) in the presence of 100 mM K+ to maintain G4 folding. Spectral profiles (j, l) and quantitation at 522 nm (k, m). Data information: (j–m) values are mean ± SEM, *n* = 3 biological replicates. All assays were performed in K+‐containing buffers to maintain physiological G4 folding, ensuring that observed resolution activity, MYC‐G4‐Pu28(5’‐FAM, 3’BHQ‐1) were diluted of 100 nM. (j, l) reflects true helicase efficiency against stable substrates. (k, m) one‐way ANOVA with Tukey's multiple comparison test. ns, not significant, ^*^ *p* < 0.05, ^**^ *p* < 0.01, ^****^ *p* < 0.0001.

Building on evidence linking TRIB3‐DDX5 to HR gene expression and G4‐related phenotypes, we next asked whether this regulatory relationship involves their engagement with G4 structures in the promoters of HR genes. To identify G4 motifs in *BRCA1* and *RAD51AP1* promoters, we first retrieved experimentally defined G4 sequences from the G4Bank database [49], then used the QGRS mapper algorithm to identify the highest scoring motifs (Table S2). Within the close head‐to‐head orientation of these genes and their direct neighbors, we identified high‐scoring G4 consensus motifs in BRCA1's first exon, an upstream motif overlapping NBR1, and two additional motifs located between RAD51AP1 and FERRY3 (Figure 7a). CUT&Tag profiling assays mapping occupancy across these regions under Gln‐deprivation conditions showed good replicate concordance for each target (Figure 7b) and detected overlapping peaks for BG4, TRIB3 and DDX5 that were coincident within the BRCA1 and RAD51AP1 promoter regions harboring the predicted G4 motifs (Figure 7c,d). The common occupancy patterns within G4 promoter structures raised the possibility that the TRIB3‐DDX5 complex engaged directly with promoter G4 motifs. Moreover, the elevated G4 signals at these loci following TRIB3 knockdown (Figure 7e,f), further advances the notion that TRIB3 may function to resolve G4 structures.

To establish the molecular basis for the recruitment of TRIB3‐DDX5 to G4‐DNA, we performed comparative pulldown assays against the MYC‐G4 positive control, assessing their relative efficiencies for recovering TRIB3 and DDX5. Surprisingly, only the BRCA1‐G4‐1 probe provided comparable target enrichment to MYC‐G4 although the other probes also selectively bound TRIB3 and DDX5 to varying extents (Figure 7g), possibly reflecting different binding affinities at individual G4 sites. Repeating these assays with stem‐loop and double‐stranded DNA (dsDNA) control probes based on the MYC‐G4, BRCA1‐G4‐1, RAD51AP1‐G4‐1 sequences (Figure S7f) confirmed that the observed interactions were specific to G4‐forming sequences.

To better understand the probe‐dependent differences, we characterized the folding topologies and thermal stabilities of each G4 probe using circular dichroism (CD) spectroscopy, examining their CD signatures and temperature‐dependent unfolding profiles [50, 51]. Consistent with established criteria, the MYC‐G4 and RAD51AP1‐G4‐2 probes predominantly adopted the parallel G4 conformation, BRCA1‐G4‐2 displayed an antiparallel‐like signature, while BRCA1‐G4‐1 and RAD51AP1‐G4‐1 exhibited hybrid‐type features (Figure 7h). CD‐melting curves reveal distinct thermal stabilities among these motifs (Figure 7i), supporting the notion that differences in G4 topology and stability contribute to the differential enrichment of TRIB3/DDX5 observed across probes in the pulldown assays.

Having established that TRIB3 recruits DDX5 to G4‐DNA, we next asked whether this recruitment enhances the G4 resolvase activity of DDX5. To evaluate this, we measured G4 helicase activity in vitro by co‐incubated fluorescently labeled MYC‐G4 probes with purified TRIB3 and DDX5 proteins (Figure S4c,d). Spectral profiles provide a visual assessment of G4 unwinding while helicase activity was quantified by fluorescence emission changes at 522 nm, as per Yang et al. [44]. As expected, TRIB3 alone demonstrated no detectable helicase activity at any concentration tested (Figure 7j,k). In contrast, DDX5 alone efficiently resolved G4 structures, and notably, the addition of TRIB3 significantly enhanced DDX5's helicase activity, achieving unwinding levels comparable to those observed in K^+^‐free conditions (Figure 7l,m). Together, these findings support a cooperative mechanism in which TRIB3 both recruits DDX5 to promoter G4 structures and enhances its resolvase activity, thereby promoting a transcriptionally permissive state at target loci.

### 2.9 TRIB3 Knockdown Promotes DNA Damage and Apoptosis under Gln‐Limited Conditions In Vivo

We hypothesized that TRIB3 supports HCC growth under metabolic stress by resolving G4‐mediated replication stress. To test this, we evaluated the role of TRIB3 in HCC proliferation and tumorigenesis under both nutrient‐replete and Gln‐deprived conditions.

Consistent with their reliance on exogenous Gln, HepG2 cells exhibited significantly reduced proliferation and diminished clonogenic capacity using in vitro growth assays. TRIB3 knockdown resulted in even stronger growth inhibition and when combined with Gln deprivation, trended to further suppress growth (Figure S8a–c). For in vivo validation, we used Huh7 cells well‐established for their robust tumorigenic capacity in xenograft studies to generate doxycycline (Dox)‐inducible TRIB3 knockdown and control isogenic sublines. After tumor initiation, mice received regular or Dox‐supplemented water with either standard or Gln‐deficient chow. Phenocopying the in vitro data, Gln restriction reduced the size of Huh7 xenografts, with TRIB3 silencing resulted in profound growth inhibition under both diets (Figure S8d–f). These findings suggest a floor effect where the growth inhibition following TRIB3 depletion limits further discrimination.

Tumor tissue analyses confirmed the induction of TRIB3 by Gln restriction and its effective knockdown by Dox treatment in the in vivo setting (Figure S8g). Moreover, directional changes in HR protein expression follow the patterns anticipated from our in vitro analyses, with increased BRCA1 and RAD51AP1 expression under Gln restriction and diminishment upon TRIB3 knockdown. Further immunohistochemical staining showed increased TRIB3 staining in control tumors under the Gln‐deficient diet, while DDX5 levels remained unchanged across conditions (Figure S8h,i). Conversely, Ki67 proliferation marker staining was reduced in control tumors under Gln restriction, although intriguingly, TRIB3‐knockdown xenografts showed similar proliferative indices despite smaller tumor volumes. The latter result suggested that alternative growth‐limiting mechanisms such as apoptosis were likely in play.

We tested this hypothesis using TUNEL assays complemented by γ‑H2A.X staining. Gln restriction alone did not significantly increase apoptosis, whereas TRIB3 knockdown modestly elevated TUNEL‑positive cells. However, when Gln restriction was combined with TRIB3 silencing, the apoptosis rates were markedly amplified (Figure S8j,k). Parallel staining for γ‐H2A.X, used here as a proxy measure of DNA damage events, showed remarkably similar findings to the TUNEL analysis with exacerbated γ‐H2A.X staining evident when combining TRIB3 knockdown with Gln restriction (Figure S8l,m).

Collectively, these data demonstrate that TRIB3 is essential for mitigating DNA damage and apoptosis triggered by Gln limitation in vivo. The marked exacerbation of γ‐H2A.X and TUNEL staining when TRIB3 knockdown was combined with Gln restriction reveals that TRIB3 critically protects against genotoxic stress, thereby supporting HCC cell survival under metabolic stress conditions.

## 3 Discussion

The UPR and DDR are increasingly recognized as interconnected pathways, with emerging evidence highlighting cross‐talk between proteostatic stress and genomic stability mechanisms [52, 53]. The TRIB family proteins act as adaptors that link and modulate multiple signaling pathways, influencing a wide range of physiological and pathological processes [54], and are well‐established components of the UPR stress‐resolution toolkit, functioning as nodal regulators that sense, integrate, and resolve cellular stress [54, 55]. TRIB3 has been discovered to associate with the E3 ubiquitin ligase COP1 to recruit protein substrates for ubiquitin‐mediated degradation including SIRT1 and FOXO1 [56, 57] while conversely, TRIB3 cooperates with WWP1 to promote EGFR protein stability [58]. In another context, TRIB3 indirectly shields FOXO1 from proteasomal degradation via SKP2 and NEDD4L [59]. Nonetheless, there have been fragmentary clues suggesting a connection to the DDR. For instance, TRIB3 is modulated by DNA‐damaging agents like cisplatin and contributes to chemoresistance [60, 61, 62, 63]. Other incidental findings include physical interactions between TRIB3 and DDR effectors such as BCLAF1 (a p53 stabilizer that antagonizes MDM2) and DDX helicases including DDX5 [64]. Yet, the field has predominantly confined TRIB proteins to UPR/ER stress biology, leaving their DDR roles unmapped.

**FIGURE 8:**
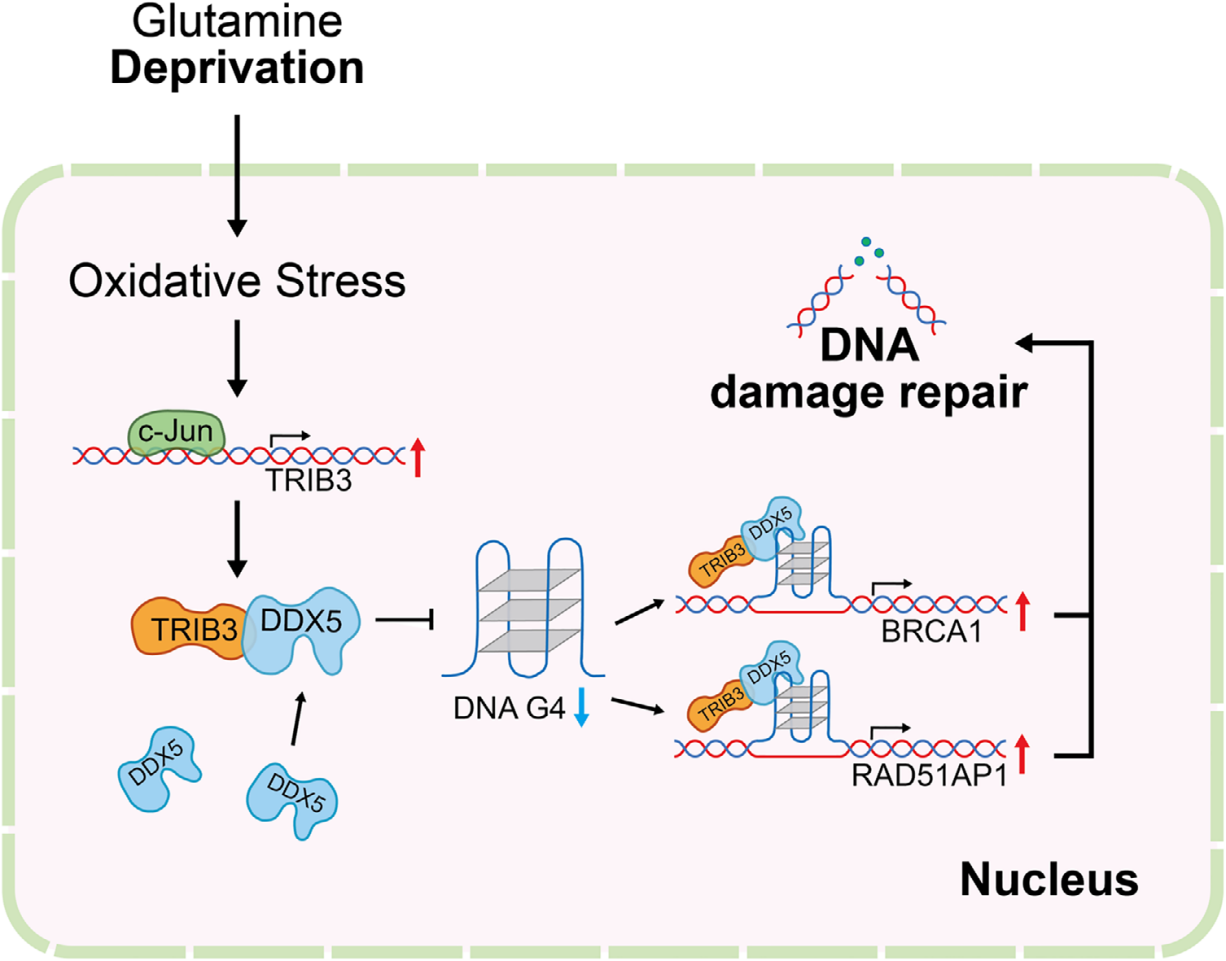
Proposed molecular mechanism of TRIB3/DDX5‐mediated G‐quadruplex resolution under glutamine (Gln) deprivation. Gln deprivation induces nuclear oxidative stress in hepatocellular carcinoma (HCC) cells, activating c‐Jun and upregulating TRIB3 transcription. The stress‐induced TRIB3 protein recruits DDX5 to resolve genomic G4 structures, facilitating transcriptional activation of homologousrecombination (HR) pathway genes (including BRCA1 and RAD51AP1) and promoting DNA damage repair, thereby countering oxidative stress‐induced genomic instability.

Here, we identify a novel function for TRIB3 in genomic stability through its role in resolving G4‐DNA structures (Figure 8). This function relies on the partnership between TRIB3 and DDX5, a multifunctional helicase with established roles in DNA repair, RNA splicing, transcriptional regulation, and as corroborated here, the unwinding of G4‐DNA [65]. During Gln deprivation, the resulting oxidative stress activates c‐Jun, in turn, transactivating TRIB3 to increase its expression in HCC cells. De novo TRIB3 binds at G4 sites to recruit DDX5 and harness its G4‐unwinding capability, establishing a dual protective role: (1) acting to clear genome‐wide G4s that otherwise stall replication forks, and (2) selectively relieving the transcriptional repression of DNA damage pathway genes. This activity, particularly under Gln deprivation, safeguards the DDR machinery, enabling cells to alleviate replication stress and prevent replication catastrophe, thus positioning TRIB3 as a critical mechanistic bridge between metabolic adaptation and genome maintenance. This advance in the understanding of TRIB3 function also justifies investigating analogous roles for other TRIB family members.

Surprisingly, we found that TRIB3 participates in genome maintenance through G4‐DNA resolution under basal conditions. Consistent with this, depleting TRIB3 or DDX5 in cells without Gln deprivation resulted in DNA damage accompanied by perturbed expression of DNA repair genes and their pathways. Moreover, we found marked G4‐DNA accumulation after knockdown of TRIB3 or DDX5, which was further enhanced following Gln withdrawal. The relationship uncovered between TRIB3 and DNA damage pathways suggests a degree of specificity, or at least preferential involvement, for TRIB3 to maintain DDR related gene expression. Our subsequent focused investigation of HR pathway genes revealed co‐occupancy of G4‐DNA, TRIB3, and DDX5 at the *BRCA1* and *RAD51AP1* promoters, with TRIB3 knockdown corresponding to increased G4 signals. Crucially, we demonstrate that TRIB3 binds G4‐DNA, which is consistent with a model in which it specifies G4 sites for DDX5 recruitment, thereby enabling DDX5's unwinding activity to relieve transcriptional repression. However, DDX5 itself is a multifunctional helicase with well‐documented roles in genome maintenance: beyond its ability to bind to and resolve G4‐DNA, it also promotes RNA:DNA hybrid (R‐loop) resolution in an ATP‐dependent manner and can functionally couple to transcriptional termination machinery (e.g., XRN2) [66]. In line with this broader functional repertoire, DDX5 has independently been reported to transcriptionally regulate MYC and Col2a1 through promoter G4 resolution [44, 67]. Notably, DDX5 overexpression only partially rescued the molecular and genomic defects associated with TRIB3 depletion, indicating that TRIB3 may engage in both locus‐specific G4 interactions and more general, context‐dependent modes of G4 regulation.

How TRIB3 achieves G4 site preferences remains an open question. G4 topologies are not uniform and can adopt diverse conformations, including parallel and hybrid structures [68, 69], and the effects of artificial G4 stabilization on individual genes also vary considerably, with *c‐MYC* and *BRCA1* being particularly pyridostatin‐sensitive. We found TRIB3 demonstrated robust binding to both parallel and hybrid G4 structures in vitro, suggesting that specificity is at least partially conferred by the underlying G4 sequence. However, neither sequence nor topology are likely to fully account for site selectivity in vivo. Additional layers of regulation such as chromatin context and interactions with unidentified DNA‐binding proteins such as transcription factors or cofactors are almost certainly involved. The prior demonstration that the co‐occurrent R‐loops and G4s may reciprocally exacerbate genomic stress [70] is of clear potential relevance. Understanding the dependencies that guide TRIB3 recruitment to specific genomic loci could provide insights into the evolution of DDR pathways and their functional integration with stress responses.

The clinical relevance of our findings emerges against the evolving therapeutic landscape for advanced HCC. Current first‐line systemic therapies for advanced/unresectable HCC are dominated by immune checkpoint inhibitor (ICI) based combinations, most notably atezolizumab plus bevacizumab and the STRIDE regimen (durvalumab plus tremelimumab) [71, 72], both providing improved outcomes compared with prior tyrosine kinase inhibitor (TKI) regimens (although lenvatinib remains an option for patients ineligible for immunotherapy/anti‐VEGF therapy) [73]. Despite these advances, disease control remains incomplete; for example, nearly half of patients receiving atezolizumab‐bevacizumab experience progression within the first 6 months, underscoring the limited durability of benefit for a substantial subset. This therapeutic limitation, combined with the rising global incidence of HCC, highlights the need for strategies targeting novel vulnerabilities. Our identification of the TRIB3‐DDX5‐G4‐DNA axis, which supports HCC cell viability through genomic maintenance, reveals one such vulnerability. TRIB3 is frequently overexpressed in HCC and correlates with aggressive disease. While TRIB3 exerts pleiotropic effects, its role in this specific axis presents new therapeutic opportunities for HCC and other malignancies characterized by oncogenic TRIB3 expression [20, 74].

One avenue to pursue would involve the development of small‐molecule inhibitors that disrupt TRIB3‐DDX5 nuclear complexes. Alternatively, analogs of pyridostatin that selectively stabilize G4 structures targeted by TRIB3‐DDX5 would conceivably impair the DDR machinery and amplify replication stress in cancer cells. Based on our findings, the most logical strategy would involve combinatorial therapies that synergize with Gln metabolism inhibitors such as CB‐839 to amplify metabolic stress. Indeed, it was encouraging to observe in our xenograft studies that switching mice to a Gln‐limited diet was sufficient to patently increase DNA damage and apoptosis in TRIB3‐deficient tumors. Similarly, co‐treatment with PARP inhibitors may improve their antagonism of BRCA1‐dependent survival pathways [75, 76]. However, the clinical feasibility of TRIB3‐targeting therapies would depend on their therapeutic window together with its overexpression status and conserved function in different cancer genomes.

Our study has several limitations. First, although we demonstrated TRIB3‐DDX5‐G4‐DNA interactions and G4 resolvase activity in vitro, future work would benefit from quantitative biophysical approaches such as bio‐layer interferometry (BLI) or surface plasmon resonance (SPR) to define binding affinities and kinetics, and to directly assess whether and how TRIB3 modulates DDX5 unwinding activity. Second, the association between TRIB3‐DDX5 and DDR pathway genes remains incompletely explained and will require systematic interrogation to determine whether this bias reflects intrinsic G4 sequence features, chromatin context, or higher‐order regulatory mechanisms. Third, while our in vivo data support a role for TRIB3 in maintaining genomic stability under nutritional stress, the absence of direct interrogation of G4 dynamics or DDX5 perturbation‐together with the pronounced growth suppression caused by TRIB3 loss‐limits mechanistic resolution and necessitates validation studies in more physiologically relevant models. Fourth, much of our evidence for altered DDR relies on γ‐H2A.X accumulation and transcriptional changes, whereas direct measurements of DNA repair processes remain limited; future studies will address this by tracking repair factor recruitment, foci dynamics, and functional repair assays. Finally, because TRIB3 can be induced by diverse tumor microenvironmental stresses, the phenotypes described here may reflect convergent signaling inputs beyond Gln limitation alone, and future efforts will be required to dissect the relative contributions of distinct stress cues within the TRIB3‐DDX5‐G4 regulatory axis.

In conclusion, TRIB3 plays a critical role in genome maintenance by resolving G4‐DNA structures through its interaction with DDX5. This function provides new insights into the interplay between UPR, DDR, and metabolic adaptation in cancers. Targeting TRIB3/DDX5/G4 axis offers promising opportunities for therapeutic intervention, with potential synergistic effects when combined with existing clinical treatments. Further research is needed to validate these findings and optimize therapeutic strategies.

## 4 Materials and Methods

### 4.1 Cell Culture

HEK 293T (Cat No. AW‐CH0004, RRID: CVCL_0063), HepG2 (Cat No. AW‐CH0092, RRID: CVCL_0027) and Huh7 (Cat No. AW‐CH0160, RRID: CVCL_0336) cell lines were purchased from Anwei‐sci Cell Center, Shanghai, China. All cells were routinely cultured in high glucose Dulbecco's modified eagle medium (DMEM) (Thermo Fisher, USA) supplemented with 10% FBS, 2 mM Gln, 1 mM pyruvate, and kept in a humidified incubator at 37°C, with 5% CO_2_. Cell authentication was performed by Short Tandem Repeat (STR) profiling and mycoplasma testing routinely conducted using the MycoBlue Mycoplasma Detector Kit (Vazyme, China). For the Gln deprivation experiments, cells were cultured in Gln‐deficient DMEM (Thermo Fisher) supplemented with 1/100 GlutaMAX supplement (Thermo Fisher) to maintain equivalent.

### 4.2 Animal Experiments

Four‐to‐five‐week‐old male BALB/c nude (nu/nu) mice were purchased from Spfbiotech (Beijing, China) and housed in the Laboratory Animal Center of Zhengzhou University under specific pathogen‐free (SPF) conditions. Experiments were initiated after 1 week acclimatization with animals assigned to experimental groups in a random manner. Suspensions of Huh7 cells stably expressing a Dox‐inducible TRIB3 knockdown construct (Tet‐on shTRIB3) or the corresponding control cells were mixed 1:1 (v/v) with Ceturegel Matrix, LDEV‐Free (Cat# 40183ES, YEASEN, China) and 5 × 10^6^ cells/100 µL immediately implanted into the inner aspect of the hind leg. Regular tumor volume measurements were initiated after 10 days implantation and once tumors reached >50 mm^3^, 0.2 mg/mL Dox (Cat#T1687L, TargetMol, China) was administered in drinking water to induce knockdown. Concurrently, the indicated mice groups were switched from standard chow to an amino acid‐modified diets (Cat#SY93GAA and Cat#SYGLNDEF, Shuyu Biotechnology, China). Dox administration/dietary interventions were continued for a further 3 weeks before humanely euthanizing the mice, determining tumor weights and volumes, and preparing tissues for downstream analyses.

### 4.3 RNA Extraction and Reverse Transcription—Quantitative Real‐Time PCR (qPCR) Analysis

Total RNA was extracted from cells using the Super FastPure Cell RNA Isolation Kit (Vazyme) according to the manufacturer's instructions. Subsequently, one µg of the total RNA was reverse transcribed into cDNA using a HiScript III All‐in‐one RT SuperMix (Vazyme). QPCR reactions (10 µl) were undertaken using the SYBR Green SupTaq HS Premix qPCR Kit (AGBio, China) containing 1 µl of cDNA template, and 0.5 µl each of the forward and reverse primers (10 µM). Relative gene expression fold changes were calculated using the 2^−ΔΔCt^ method based on threshold cycle (Ct) values using the beta‐actin primers as the reference. The primer sequences are shown in the Table S3.

### 4.4 Western Blotting

Cultured cells were harvested and lysed in RIPA buffer (Beyotime, China) supplemented with protease inhibitor cocktail (Roche, USA). Pre‐cleared lysates were obtained by centrifugation at 12,000 × *g* for 15 min at 4°C and protein concentrations quantified using the Pierce Rapid Gold BCA Assay (Thermo Fisher). Alternatively, proteins were extracted from tissues using a tissue homogenizer (KZ‐II, Servicebio) and then lysed with RIPA buffer. Protein samples (20 µg/lane) were separated on 12% Tris‐glycine gels and transferred to nitrocellulose membranes (PALL, USA). After blocking with 5% non‐fat milk in TBST, membranes were incubated with primary antibodies at 4°C overnight and species‐matched HRP‐conjugated secondary antibodies at room temperature (RT) for 1.5 h. Signals were detected with SuperSignal West Pico PLUS Chemiluminescence Substrate (Thermo Fisher) on a ChemiDoc MP Imager (Bio‐Rad, USA). The antibodies used are shown in Table S4.

### 4.5 RNA Interference and Gene Overexpression

Stable gene knockdown using short hairpin RNAs (shRNAs) and overexpression was accomplished using lentiviral mediated transduction. Lentiviral particles for knockdown experiments were prepared by transfecting HEK 293T cells with pLKO.1 or FH1t(UTG)‐based shRNAs, and the packaging plasmids pREV, pGag, and pVSVG at 2:2:2:1 ratio. For overexpression, the pCDH (containing the gene of interest), pREV, pGag, and pVSVG vectors were transfected at a 2:2:2:1 ratio. After 48 h, viral supernatants were filtered with 0.45 µm filter membrane and used directly to infect target cells in presence of 10 µg/ml polybrene (Sigma, USA). Culture supernatants were removed and cells selected with 4 µg/ml puromycin treatment for 48 h. Targeting sequences are shown in Table S3. Alternatively, transient expression experiments were performed using the Lipofectamine 2000 reagent (Cat#11668019, Thermo Fisher). Plasmid DNA‐Lipofectamine 2000 complexes prepared in Opti‐MEM were used to transfect target cells for 6 h before replacing the transfection medium with complete growth medium. Subsequent treatments were performed 24 h post‐transfection.

### 4.6 Alkaline Comet Assay (Single‐Cell Gel Electrophoresis, SCGE)

Alkaline comet assays were performed using the Comet Assay Kit (R&D Systems, USA) according to the manufacturer's instructions. Briefly, pretreated HepG2 cells (3000 cells/sample) were embedded in low‐melting‐point agarose and immobilized on comet assay slides. Following overnight lysis at 4°C, slides were subjected to alkaline unwinding in freshly prepared solution (200 mM NaOH, 1 mM EDTA, pH > 13) for 1 h. Electrophoresis was then performed in alkaline electrophoresis solution (200 mM NaOH, 1 mM EDTA, pH > 13) at 1 V/cm for 30 min (300 mA constant current). Slides were subsequently dried at 37°C, stained with SYBR Gold for 30 min at RT, and visualized by fluorescence microscopy. Tail movement was subsequently analyzed with the CASP software.

### 4.7 Immunofluorescence Staining

Cells were seeded and cultured on 14‐mm TC‐treated coverslips prior to fixation with 4% paraformaldehyde (PFA) for 15 min at RT. After twice washing with PBS (5 min/wash), cells were permeabilized with 0.1% Triton X‐100 in PBS for 10 min at RT. Afterwards, cells were blocked with PBST (0.1% Tween‐20 in PBS) containing 0.5% goat serum for 1 h at RT. For experiments involving BG4 detection [77], the cells were incubated with 0.25 mg/mL RNase A for 1 h at 37°C, followed by three washes before blocking. Primary antibodies were diluted in blocking buffer and incubated overnight at 4°C before washing three times with PBST and the addition of fluorophore‐conjugated species‐matched secondary antibodies for 1.5 h at RT. Where indicated, F‐actin was stained using Actin‐Tracker Green‐488 probe (Cat#C2201S, Beyotime) before mounting coverslips using ProLong Glass Antifade Mountant with NucBlue (Thermo Fisher). Confocal images were acquired using a TCS SP8 Laser Confocal Microscope (Leica microsystems, Germany). The antibodies used are shown in Table S4, and the BG4‐FLAG recombinant protein was a kindly gift from Professor Lianxin Liu in USTC.

### 4.8 Reactive Oxygen Species Detection

Cellular reactive oxygen species (ROS) was detected using ROS assay kit (S0033, Beyotime) following the manufacturers’ instructions.

### 4.9 Nuclear Hydrogen Peroxide Detection

Nuclear hydrogen peroxide measurements were performed according to the protocol of Pak et al. [40]. Briefly, HepG2 cells were transiently transfected with pCDH‐NLS‐HyPer7 plasmid. At 24 h post‐transfection, cells were harvested and seeded onto 14 mm TC‐treated coverslips. Following overnight attachment, cells were subjected to Gln deprivation, then fixed with 4% PFA. Coverslips were mounted using antifade mounting medium (Beyotime) and imaged by confocal microscopy with sequential excitation at 405 and 488 nm. Emission was captured at 520 nm, and the Ex_500_/Ex_400_ ratio was calculated for quantitative analysis.

### 4.10 Reduced Glutathione Detection

Reduced GSH was detected using the GSH and GSSG assay kit (S0053, Beyotime) following the manufacturers’ instructions.

### 4.11 Glutamic Acid Assay

Glu was detected using the Glutamic Acid Colorimetric Assay Kit (E‐BC‐K903‐M, Elabscience, China) following the manufacturers’ instructions.

### 4.12 RNA‐seq Analysis

Total RNA was extracted from HepG2 cells treated with Gln deprivation, and RNA integrity was verified by Bioanalyzer (RIN > 8.0). Strand‐specific RNA‐seq libraries were prepared with the NEBNext Ultra II Kit and sequenced on an Illumina NovaSeq 6000 platform (PE150). Reads were aligned to the GRCh38 genome using STAR, and differential expression analysis was performed with DESeq2 (FDR < 0.05). Kyoto Encyclopedia of genes and genomes (KEGG) and GSEA were analyzed, respectively. Sequencing data were deposited in the National Center for Biotechnology Information Gene Expression Omnibus database (GSE307270, GSE307271).

### 4.13 Immunoprecipitation Assays

Cells were first washed with ice cold PBS and harvested using cell scrapers before lysis in Immunoprecipitation (IP) buffer (25 mM Tris‐HCl, 150 mM NaCl, 2.5 mM MgCl_2_, 10% glycerol, 2 mM EDTA, 0.5% Triton X‐100, 0.5% NP‐40, protease inhibitors, pH 7.5). Lysates were clarified by centrifugation with all subsequent steps performed at 4°C. To immunoprecipitate epitope‐tagged proteins, lysates were incubated with anti‐FLAG (L‐1011, Bio‐linkedin, China) or anti‐HA (L‐1009, Bio‐linkedin) magnetic beads overnight. For endogenous targets, lysates were precleared using Protein G Dynabeads (Cat#10004D, Thermo Fisher) before adding 1 µg control IgG, anti‐TRIB3 (ab75846, Abcam) or anti‐DDX5 (67025‐1‐Ig, Proteintech) antibodies to the precleared lysates overnight. Followed by Protein G capture for 2 h, lysates were then washed five times with IP‐lysis buffer and immunoprecipitants eluted with 20 µL 1×SDS loading buffer. For in vitro IP assays, purified recombinant proteins (as indicated) were mixed in IP buffer and incubated at 4°C with rotation to allow complex formation. Complexes were captured using tag‐specific affinity beads (e.g., anti‐FLAG magnetic beads for FLAG‐tagged bait, or His‐tag capture beads/resin for His‐tagged bait) under the same temperature conditions. Beads were then washed five times with IP buffer and bound proteins were eluted with 1× SDS loading buffer. IP samples were analyzed by immunoblotting using 10% of input lysate as a control.

### 4.14 Dual Luciferase Reporter Assay

Dual Luciferase reporter assays were conducted using the Dual‐Glo Luciferase Assay kit (E1910, Promega, USA) following the manufacturer's protocol. Briefly, HEK 293T cells seeded into 24‐well culture plates were transfected with the pGL3‐Basic, pGL3‐TRIB3‐promoter‐1 or pGL3‐TRIB3‐promoter‐2 firefly luciferase reporter plasmids in conjunction with pcDNA3.1 or pcDNA3.1‐FLAG‐JUN and Renilla reference plasmids (4:4:2 ratio). After 24 h, cells were washed twice with PBS and lysed using Passive Lysis Buffer before clarification by centrifugation (12,000 × *g*, 10 min, 4°C). Lysate aliquots (10 µL) were added to black‐walled, clear‐bottom 96‐well plates. Firefly and Renilla luciferase activity measurements were performed using a Varioskan LUX plate reader with sequential injection of 100 µL Luciferase Assay Reagent II (LAR II) followed by 100 µL Stop & Glo Reagent. Reporter activity was normalized as Firefly/Renilla 0 (RLU) ratios.

### 4.15 G4 streptavidin Pulldown Assay

The graphical model of G4 streptavidin pulldown assay was shown in Figure S6c. Briefly, lyophilized DNA probes (conjugate with biotin) were annealed in buffer (10 mM Tris‐HCl, pH 7.5, 110 mM KCl). HepG2 cells were washed with ice‐cold PBS. Pellets were resuspended in 300 µL hypotonic buffer (20 mM HEPES‐NaOH, pH 7.4, 10 mM NaCl, 3 mM MgCl_2_, 0.2 mM EDTA, 1 mM DTT, 1×protease inhibitors). After 15 min incubation on ice, 15 µL of 10% NP‐40 (Sigma) was added followed by vortex mixing. Nuclei were pelleted (900×g, 10 min, 4°C) and washed in hypotonic buffer. Nuclear pellets were resuspended in 100 µL high‐salt extraction buffer (20 mM HEPES‐KOH, pH 7.4, 0.5 M NaCl, 3 mM MgCl_2_, 0.2 mM EDTA, 1 mM DTT, 0.5% NP‐40, 1×protease inhibitors). Chromatin was sheared by ultrasonication. Lysates were clarified and supernatants quantified by BCA assay. Streptavidin magnetic beads (50 µL/sample; L‐1012, Bio‐linkedin) were pre‐blocked with 3% BSA (A7906, Sigma) and 0.2 mg/mL sheared salmon sperm DNA (Cat#15632011, Invitrogen) in binding buffer (25 mM HEPES, pH 7.5, 10.5 mM NaCl, 110 mM KCl, 1 mM MgCl_2_, 0.01 mM ZnCl_2_, 20% glycerol, 0.5% NP‐40, 1 mM DTT). Nuclear extracts (75 µg) were pre‐cleared with blocked beads. Fresh beads (50 µL/sample) were incubated with 5 pmol annealed G4 probes in 500 µL binding buffer (30 min, rotation at RT). Probe‐bound beads were washed three times and incubated with pre‐cleared nuclear proteins overnight at 4°C. For in vitro pulldown assays, probe‐bound beads were incubated with purified recombinant proteins (as indicated) in binding buffer in a final volume of 500 µL (rotation). After incubation, beads were captured magnetically and washed five times with binding buffer. Bound proteins were eluted by boiling in SDS sample buffer and analyzed by Western blotting. Beads‐only or non‐G4/mutant probes were included as negative controls. The DNA probes used in this study are summarized in Table S3.

### 4.16 CUT&Tag

CUT&Tag libraries were generated following the manufacturer's protocol (Vazyme, TD903‐01). Library quality control (QC) and sequencing were performed commercially by Frasergen (Wuhan, China). For each condition, two independent technical replicates were performed. Data cleaning and analysis involved trimming the adapters of reads and removing low‐quality bases by Trim_Galore software in paired‐end mode, mapping clean reads to the hg38 reference genome with Bowtie2 followed by removing PCR duplicates using Picard. Genome browser tracks in bigwig format were produced from replicates using deepTools and peaks were called with MACS2 using parameters “‐f BAMPE‐broad‐nomodel”. Bigwig data was visualized using the IGV software (Broad Institute). High‐confidence peaks were defined as peaks reproducibly detected across biological replicates. Genome‐wide signal comparison between samples was performed using deepTools. First, signal tracks in bigWig format were compared using bigwigCompare. In this step, the genome is divided into equally sized bins, and the read coverage signal from each bigWig file is calculated per bin. The resulting signal values are then summarized as a ratio, log2 ratio, difference, or other defined operations. In this study, the average log2 ratio between two groups was calculated to represent relative enrichment differences at the same scale across the genome. The sequencing data were deposited in the National Center for Biotechnology Information Gene Expression Omnibus database (GSE306768).

### 4.17 Proximity Ligation Assays

Assays were performed using the Duolink in Situ PLA kit (Sigma), following the manufacturer's instructions with modifications accordingly to Alagia et al. [78]. Briefly, cells seeded and cultured on 8 mm TC‐treated coverslips were washed with ice‐cold PBS and then fixed with 4% PFA for 15 min. After washing with PBS, cells were permeabilized with 0.1% Triton X‐100 for 10 min at RT followed by blocking with 40 µL blocking solution at 37°C for 1 h. Thereafter, primary antibody mixtures were incubated at 4°C overnight. For G4‐DNA detection, RNase A pretreatment was performed prior to blocking (as described above) with sequential application of antibodies, first anti‐FLAG overnight followed by FLAG + co‐antibody mixture incubation overnight. After washing, PLUS/MINUS probes (1:5 in diluent) were hybridized at 37°C for 1 h before ligase and polymerase amplification reactions for 30 and 100 min at 37°C, respectively. Slides were mounted with ProLong Glass + NucBlue (Cat# P36981, Thermo Fisher) and imaged by confocal microscopy (Leica TCS SP8).

### 4.18 Chromatin Immunoprecipitation Assay

Chromatin immunoprecipitation (ChIP) assays were performed according to the manufacturer's instructions (Beyotime) using rabbit IgG as the negative control. The DNA fragments of interest were analyzed by PCR using specific primers. All primer sequences are summarized in Table S3.

### 4.19 CD Spectra and Thermal Stability Assay

Oligonucleotide probes dissolved in annealing buffer (10 mM Tris and 110 mM KCl) were folded by heating followed by gradual cooling to room temperature in a thermocycler. CD measurements were performed using a J‐1700 CD spectrometer (JASCO). CD spectra were recorded from 220–350 nm at a scan depth of 200 mdeg/1.0 dOD, with a 0.5 nm step size. For thermal stability analysis, thermal denaturation was monitored by recording the CD signal from 25°C to 98°C at the wavelength corresponding to the maximum CD signal for each sequence determined from the spectra. The DNA probes used in this study are summarized in Table S3.

### 4.20 G4‐DNA Helicase Activity Assay

G4 structures formed by MYC‐G4 oligonucleotides were prepared with or without 100 mM KCl by heating solutions at 95°C for 5 min followed by incremental cooled (−1°C/min) to 25°C. Unwinding reactions were performed in 10 µL in black‐walled, clear‐bottom 384‐well microplates consisting of 100 nM MYC‐G4 probe in 50 mM Tris‐acetate pH 7.2, 2.5 mM MgCl_2_, 0.5 mM DTT buffer in the presence of varying concentrations of recombinant TRIB3‐His (Cat#AR51742PU, Origene, China), or 100 nM DDX5‐FLAG (Cat#TP300371, Origene) proteins. Reaction mixtures were incubated at 37°C for 5 min, with fluorescence detection (Ex 490 nm, Em 512–602 nm, 10 nm step length) performed using a Synergy H1 Multimode Microplate Reader (Bio‐Tek, USA). The DNA probes used in this study are summarized in Table S3.

### 4.21 Cell Proliferation Assay

Cell proliferation assays were preformed using the Cell counting kit‐8 (Cat#C0005, TargetMol) following the manufacturers’ instructions. Briefly, infected HepG2 cells were seeded in 96‐well culture plates at a density of 3000 cells/well. On days 1–4 post‐seeding, the assays were performed and the absorbance at 450 nm was measured using a Varioskan LUX microplate reader (Thermo Fisher), respectively.

### 4.22 Colony Formation Assay

The colony formation assays were performed to evaluate single‐cell proliferative capacity. HepG2 cells transduced with control shRNA or TRIB3‐targeting shRNA lentiviral particles were seeded in 6‐well plates at 1000 cells/well. Cells were maintained in Gln‐deficient medium for 12 days. Colonies were fixed with ice‐cold methanol for 10 min, stained with 1% (w/v) crystal violet for 15 min, thoroughly rinsed with deionized water, and air‐dried. Colonies (>50 cells) were quantified using Image J with particle analysis module. The colony formation ability was calculated with the following formula: clone‐forming efficiency ratio = (number of clones/number of cells inoculated) × 100%.

### 4.23 Homologous Recombination Efficiency Assay

Homologous recombination (HR) efficiency was quantified using an I‐SceI endonuclease‐based reporter system. Briefly, HepG2 cells were seeded in 6‐well plates at 50% confluency and transiently co‐transfected with the DR‐GFP reporter plasmid, pCBASceI (I‐SceI expression vector), and pCDH‐mCherry‐puro (transfection control) at 1:1:1 mass ratio (total 3 µg DNA) using Lipofectamine 2000 reagent. After 24 h, cells were subjected to Gln deprivation for different durations (0, 8, 16, or 24 h), and then harvested and analyzed on a FACS Aria III flow cytometer (BD Biosciences). HR efficiency was calculated as the percentage of mCherry‐positive cells exhibiting GFP signal (FITC‐positive).

### 4.24 Immunohistochemistry and TUNEL Staining

Five µm coronal sections prepared from FFPE xenografted tumor tissues from the animal experiments were deparaffinized and rehydrated. Immunohistochemistry (IHC) staining was performed using the indicated primary antibodies (Ki67 or TRIB3 antibodies diluted at 1: 200, DDX5 antibody diluted at 1: 400) with visualization using biotin‐conjugated secondary antibodies in combination with the SABC‐HRP kit (Beyotime) and DAB Peroxidase Substrate Kit (Beyotime). Whole slide images were acquired using the TISSUEFAXS system (Tissue Gnostics Asia Pacific Limited), using image snapshots captured in Caseviewer software to count positive cells with Image J software. For TUNEL staining, tissues were treated with protease K at 37°C for 20 min, and permeabilized with 0.1% Triton X‐100 at RT for 15 min. Slides were stained using One Step TUNEL Apoptosis Assay Kit (Beyotime) and mounted with ProLong Glass + NucBlue (P36981, Thermo Fisher). After acquiring confocal images, staining was quantified with Image J software.

### 4.25 Statistical Analysis

Statistical analysis was carried out using GraphPad Prism to assess differences between experimental groups. Statistical significance was analyzed by unpaired two‐tailed Student's *t*‐test for comparisons of two groups, one‐way ANOVA with Tukey's post‐test for comparisons of multi groups, two‐way ANOVA with Tukey's post‐test for bivariate comparisons. *p* values lower than 0.05 were considered to be statistically significant. (ns, not significant, ^*^ *p* < 0.05, ^**^ *p* < 0.01, ^***^ *p* < 0.001, ^****^ *p* < 0.0001.) All experiments were performed in triplicates.

## Author Contributions

Conceptualization: M.W., X.Y.L., Q.J., X.D.S.; Methodology: Q.J., X.D.S., Z.R.S., M.F.L.; Data analysis and curation, Q.J., X.D.S., X.Y.C., S.T., J.M.L.; Supervision: M.W., X.Y.L., G.Z.L.; Writing – original draft preparation: Q.J. X.D.S., X.Y.L.; Writing – review and editing: R.F.T., M.W.; funding acquisition: M.W., X.Y.L.; All authors approved the final version of the manuscript.

## Funding

The authors acknowledge the support of the National Natural Science Foundation of China (Grant Nos. 82472856, 32270818, 82372661, 32570636 and 32541002).

## Ethical approval statement

Animal studies complied with all relevant ethical regulations for animal research under the approval of the Institutional Animal Care and Use Committee of Zhengzhou University (Approval No. ZZU‐LAC20241115[50]).

## Conflicts of Interest

The authors declare no potential conflicts of interest.

## Supporting information




**Supporting File**: advs74697‐sup‐0001‐SuppMat.docx.

## Data Availability

The sequencing data (e.g., RNA‐seq and CUT&Tag data) underlying this article are available in the National Center for Biotechnology Information Gene Expression Omnibus database (GSE307270, GSE307271, GSE306768). Other data (e.g., LC‐MS data, primers, probes) underlying this article are available in the article and in its online supplementary material.

## References

[1] H. B. El‐Serag and K. L. Rudolph , “Hepatocellular Carcinoma: Epidemiology and Molecular Carcinogenesis,” Gastroenterology 132, no. 7 (2007): 2557–2576, 10.1053/j.gastro.2007.04.061. 17570226

[2] L. Xia , L. Oyang , J. Lin , et al., “The Cancer Metabolic Reprogramming and Immune Response,” Molecular Cancer 20, no. 1 (2021): 28, 10.1186/s12943-021-01316-8. 33546704 PMC7863491

[3] E. Roth , “Nonnutritive Effects of Glutamine,” The Journal of Nutrition 138, no. 10 (2008): 2025S–2031S, 10.1093/jn/138.10.2025S. 18806119

[4] J. Son , C. A. Lyssiotis , H. Ying , et al., “Glutamine Supports Pancreatic Cancer Growth Through a KRAS‐regulated Metabolic Pathway,” Nature 496, no. 7443 (2013): 101–105, 10.1038/nature12040. 23535601 PMC3656466

[5] D. Du , C. Liu , M. Qin , et al., “Metabolic Dysregulation and Emerging Therapeutical Targets for Hepatocellular Carcinoma,” Acta Pharm Sin B 12, no. 2 (2022): 558, 10.1016/j.apsb.2021.09.019. 35256934 PMC8897153

[6] Y. Liu , Z. Miao , and Q. Yang , “AGC1‐mediated Metabolic Reprogramming and Autophagy Sustain Survival of Hepatocellular Carcinoma Cells Under Glutamine Deprivation,” Cell Biochemistry and Biophysics 82, no. 3 (2024): 2037–2043, 10.1007/s12013-024-01311-y. 38789662

[7] E. M. Flowers , J. Sudderth , L. Zacharias , et al., “Lkb1 deficiency Confers Glutamine Dependency in Polycystic Kidney Disease,” Nature communications 9, no. 1 (2018): 814, 10.1038/s41467-018-03036-y. PMC5827653 29483507

[8] A. Cacace , M. Sboarina , T. Vazeille , and P. Sonveaux , “Glutamine Activates STAT3 to Control Cancer Cell Proliferation Independently of Glutamine Metabolism,” Oncogene 36, no. 15 (2017): 2074–2084, 10.1038/onc.2016.364. 27748760 PMC5245769

[9] P. S. Liu , Y. T. Chen , X. Li , et al., “CD40 signal Rewires Fatty Acid and Glutamine Metabolism for Stimulating Macrophage Anti‐tumorigenic Functions,” Nature Immunology 24, no. 3 (2023): 452–462, 10.1038/s41590-023-01430-3. 36823405 PMC9977680

[10] A. Halama , M. Kulinski , S. S. Dib , et al., “Accelerated Lipid Catabolism and Autophagy Are Cancer Survival Mechanisms Under Inhibited Glutaminolysis,” Cancer letters 430 (2018): 133, 10.1016/j.canlet.2018.05.017. 29777783

[11] H. C. Yoo , Y. C. Yu , Y. Sung , and J. M. Han , “Glutamine Reliance in Cell Metabolism,” Experimental & Molecular Medicine 52, no. 9 (2020): 1496–1516, 10.1038/s12276-020-00504-8. 32943735 PMC8080614

[12] T. C. Welbourne , “Ammonia Production and Glutamine Incorporation Into Glutathione in the Functioning Rat Kidney,” Canadian Journal of Biochemistry 57, no. 3 (1979): 233–237, 10.1139/o79-029. 436006

[13] L. Chen and H. Cui , “Targeting Glutamine Induces Apoptosis: A Cancer Therapy Approach,” International Journal of Molecular Sciences 16, no. 9 (2015): 22830–22855, 10.3390/ijms160922830. 26402672 PMC4613338

[14] M. L. Schulte , A. Fu , P. Zhao , et al., “Pharmacological Blockade of ASCT2‐dependent Glutamine Transport Leads to Antitumor Efficacy in Preclinical Models,” Nature Medicine 24, no. 2 (2018): 194–202, 10.1038/nm.4464. PMC5803339 29334372

[15] M. I. Gross , S. D. Demo , J. B. Dennison , et al., “Antitumor Activity of the Glutaminase Inhibitor CB‐839 in Triple‐negative Breast Cancer,” Molecular Cancer Therapeutics 13, no. 4 (2014): 890–901, 10.1158/1535-7163.Mct-13-0870. 24523301

[16] P. A. Eyers , K. Keeshan , and N. Kannan , “Tribbles in the 21st Century: The Evolving Roles of Tribbles Pseudokinases in Biology and Disease,” Trends in Cell Biology 27, no. 4 (2017): 284–298, 10.1016/j.tcb.2016.11.002. 27908682 PMC5382568

[17] Y. Takahashi , N. Ohoka , H. Hayashi , and R. Sato , “TRB3 Suppresses Adipocyte Differentiation by Negatively Regulating PPARγ Transcriptional Activity,” Journal of Lipid Research 49, no. 4 (2008): 880–892, 10.1194/jlr.M700545-JLR200. 18187772

[18] H. Wang , L. Liang , Y. Xie , et al., “Pseudokinase TRIB3 Stabilizes SSRP1 via USP10‐mediated Deubiquitination to Promote Multiple Myeloma Progression,” Oncogene 44, no. 10 (2025): 694–708, 10.1038/s41388-024-03245-4. 39653795

[19] A. J. Bowers , S. Scully , and J. F. Boylan , “SKIP3, a Novel Drosophila tribbles Ortholog, Is Overexpressed in human Tumors and Is Regulated by Hypoxia,” Oncogene 22, no. 18 (2003): 2823–2835, 10.1038/sj.onc.1206367. 12743605

[20] X. J. Wang , F. F. Li , Y. J. Zhang , M. Jiang , and W. H. Ren , “TRIB3 promotes Hepatocellular Carcinoma Growth and Predicts Poor Prognosis,” Cancer Biomarkers 29, no. 3 (2020): 307–315, 10.3233/cbm-201577. 32716348 PMC12662516

[21] S. Zhou , H. Xu , and T. Wei , “Inhibition of Stress Proteins TRIB3 and STC2 Potentiates Sorafenib Sensitivity in Hepatocellular Carcinoma,” Heliyon 9, no. 6 (2023): e17295, 10.1016/j.heliyon.2023.e17295. 37389061 PMC10300369

[22] X. Y. Wang , Y. Liao , R. Q. Wang , et al., “Tribbles Pseudokinase 3 Converts Sorafenib Therapy to Neutrophil‐Mediated Lung Metastasis in Hepatocellular Carcinoma,” Adv Sci (Weinh) 12, no. 13 (2025): 2413682, 10.1002/advs.202413682. 39932456 PMC11967757

[23] M. Wennemers , J. Bussink , B. Scheijen , et al., “Tribbles Homolog 3 Denotes a Poor Prognosis in Breast Cancer and Is Involved in Hypoxia Response,” Breast Cancer Research 13, no. 4 (2011): R82, 10.1186/bcr2934. 21864376 PMC3236345

[24] N. Ohoka , S. Yoshii , T. Hattori , K. Onozaki , and H. Hayashi , “TRB3, a novel ER stress‐inducible gene, is induced via ATF4–CHOP pathway and is involved in cell death,” The EMBO Journal 24, no. 6 (2005): 1243–1255, 10.1038/sj.emboj.7600596. 15775988 PMC556400

[25] S. G. Sims and G. P. Meares , “Janus Kinase 1 Is Required for Transcriptional Reprograming of Murine Astrocytes in Response to Endoplasmic Reticulum Stress,” Frontiers in Cellular Neuroscience 13 (2019): 446, 10.3389/fncel.2019.00446. 31680865 PMC6797841

[26] S. H. Ahn , S. K. Jang , M. J. Kim , et al., “Downregulation of TRIB3 Enhances the Sensitivity of Lung Cancer Cells to Amino Acid Deprivation by Suppressing AKT Activation,” Am J Cancer Res 14, no. 4 (2024): 1622–1633, 10.62347/glsy2976. 38726284 PMC11076249

[27] R. Schwarzer , S. Dames , D. Tondera , A. Klippel , and J. Kaufmann , “TRB3 is a PI 3‐kinase Dependent Indicator for Nutrient Starvation,” Cellular Signalling 18, no. 6 (2006): 899–909, 10.1016/j.cellsig.2005.08.002. 16129579

[28] C. Jousse , C. Deval , A.‐C. Maurin , et al., “TRB3 inhibits the Transcriptional Activation of Stress‐regulated Genes by a Negative Feedback on the ATF4 Pathway,” Journal of Biological Chemistry 282, no. 21 (2007): 15851–15861, 10.1074/jbc.M611723200. 17369260

[29] T. Örd , D. Örd , P. Adler , J. Vilo , and T. Örd , “TRIB3 enhances Cell Viability During Glucose Deprivation in HEK293‐derived Cells by Upregulating IGFBP2, a Novel Nutrient Deficiency Survival Factor,” Biochimica et biophysica acta 1853, no. 10 Pt A (2015): 2492–2505, 10.1016/j.bbamcr.2015.06.006. 26094770

[30] T. Geng , W. Hu , M. H. Broadwater , et al., “Fatty Acids Differentially Regulate Insulin Resistance Through Endoplasm Reticulum Stress‐mediated Induction of Tribbles Homologue 3: A Potential Link Between Dietary Fat Composition and the Pathophysiological Outcomes of Obesity,” Diabetologia 56, no. 9 (2013): 2078–2087, 10.1007/s00125-013-2973-2. 23820633

[31] P. M. Pitale , I. V. Saltykova , Y. Adu‐Agyeiwaah , et al., “Tribbles Homolog 3 Mediates the Development and Progression of Diabetic Retinopathy,” Diabetes 70, no. 8 (2021): 1738–1753, 10.2337/db20-1268. 33975909 PMC8385618

[32] M. Gellert , M. N. Lipsett , and D. R. Davies , “Helix Formation by Guanylic Acid,” Proceedings of the National Academy of Sciences U S A 48, no. 12 (1962): 2013–2018, 10.1073/pnas.48.12.2013. PMC221115 13947099

[33] D. Sen and W. Gilbert , “Formation of Parallel Four‐stranded Complexes by Guanine‐rich Motifs in DNA and Its Implications for Meiosis,” Nature 334, no. 6180 (1988): 364–366, 10.1038/334364a0. 3393228

[34] J. L. Huppert and S. Balasubramanian , “Prevalence of Quadruplexes in the human Genome,” Nucleic Acids Research 33, no. 9 (2005): 2908–2916, 10.1093/nar/gki609. 15914667 PMC1140081

[35] J. Robinson , F. Raguseo , S. P. Nuccio , D. Liano , and M. Di Antonio , “DNA G‐quadruplex Structures: More Than Simple Roadblocks to Transcription?,” Nucleic Acids Research 49, no. 15 (2021): 8419–8431, 10.1093/nar/gkab609. 34255847 PMC8421137

[36] A. M. Zahler , J. R. Williamson , T. R. Cech , and D. M. Prescott , “Inhibition of telomerase by G‐quartet DMA structures,” Nature 350, no. 6320 (1991): 718–720, 10.1038/350718a0. 2023635

[37] K. Paeschke , M. L. Bochman , P. D. Garcia , et al., “Pif1 family Helicases Suppress Genome Instability at G‐quadruplex Motifs,” Nature 497, no. 7450 (2013): 458–462, 10.1038/nature12149. 23657261 PMC3680789

[38] J. Lopes , A. Piazza , R. Bermejo , et al., “G‐quadruplex‐induced Instability During Leading‐strand Replication,” Embo J 30, no. 19 (2011): 4033–4046, 10.1038/emboj.2011.316. 21873979 PMC3209785

[39] T. Q. Tran , M. B. Ishak Gabra , X. H. Lowman , et al., “Glutamine Deficiency Induces DNA Alkylation Damage and Sensitizes Cancer Cells to Alkylating Agents Through Inhibition of ALKBH Enzymes,” PLoS Biology 15, no. 11 (2017): 2002810, 10.1371/journal.pbio.2002810. PMC5673162 29107960

[40] V. V. Pak , D. Ezerina , O. G. Lyublinskaya , et al., “Ultrasensitive Genetically Encoded Indicator for Hydrogen Peroxide Identifies Roles for the Oxidant in Cell Migration and Mitochondrial Function,” Cell Metabolism 31, no. 3 (2020): 642–653.e6, 10.1016/j.cmet.2020.02.003. 32130885 PMC7088435

[41] M. Li , R. F. Thorne , R. Shi , et al., “DDIT3 Directs a Dual Mechanism to Balance Glycolysis and Oxidative Phosphorylation During Glutamine Deprivation,” Advanced Science (Weinheim) 8, no. 11 (2021): 2003732, 10.1002/advs.202003732. PMC8188220 34105294

[42] J. Wang , “Integrated Proteomic and Metabolomic Analysis of Muscle Atrophy Induced by Hindlimb Unloading,” Biomolecules 15, no. 7 (2024): 14, 10.3390/biom14070749. 39858409 PMC11764416

[43] P. Haberzettl and B. G. Hill , “Oxidized Lipids Activate Autophagy in a JNK‐dependent Manner by Stimulating the Endoplasmic Reticulum Stress Response,” Redox Biology 1, no. 1 (2013): 56, 10.1016/j.redox.2012.10.003. 24024137 PMC3757667

[44] G. Wu , Z. Xing , E. J. Tran , and D. Yang , “DDX5 helicase Resolves G‐quadruplex and Is Involved in MYC Gene Transcriptional Activation,” Proceedings of the National Academy of Sciences U S A 116, no. 41 (2019): 20453–20461, 10.1073/pnas.1909047116. PMC6789965 31548374

[45] G. Biffi , D. Tannahill , J. McCafferty , and S. Balasubramanian , “Quantitative Visualization of DNA G‐quadruplex Structures in human Cells,” Nature Chemistry 5, no. 3 (2013): 182–186, 10.1038/nchem.1548. PMC3622242 23422559

[46] R. Rodriguez , K. M. Miller , J. V. Forment , et al., “Small‐Molecule–Induced DNA Damage Identifies Alternative DNA Structures in Human Genes,” Nature Chemical Biology 8, no. 3 (2012): 301–310, 10.1038/nchembio.780. 22306580 PMC3433707

[47] K. I. E. McLuckie , M. Di Antonio , H. Zecchini , et al., “G‐quadruplex DNA as a Molecular Target for Induced Synthetic Lethality in Cancer Cells,” Journal of the American Chemical Society 135, no. 26 (2013): 9640–9643, 10.1021/ja404868t. 23782415 PMC3964824

[48] G. Miglietta , M. Russo , R. C. Duardo , and G. Capranico , “G‐quadruplex Binders as Cytostatic Modulators of Innate Immune Genes in Cancer Cells,” Nucleic Acids Research 49, no. 12 (2021): 6673, 10.1093/nar/gkab500. 34139015 PMC8266585

[49] H. S. Zhong , M. J. Dong , and F. Gao , “G4Bank: A Database of Experimentally Identified DNA G‐quadruplex Sequences,” Interdisciplinary Sciences 15, no. 3 (2023): 515–523, 10.1007/s12539-023-00577-9. 37389723

[50] M. Vorlíčková , I. Kejnovská , J. Sagi , et al., “Circular Dichroism and Guanine Quadruplexes,” Methods (San Diego, California) 57, no. 1 (2012): 64, 10.1016/j.ymeth.2012.03.011. 22450044

[51] R. Del Villar‐Guerra , J. O. Trent , and J. B. Chaires , “G‐Quadruplex Secondary Structure Obtained From Circular Dichroism Spectroscopy,” Angewandte Chemie International Edition 57, no. 24 (2018): 7171–7175, 10.1002/anie.201709184. 29076232 PMC5920796

[52] M. González‐Quiroz , A. Blondel , A. Sagredo , C. Hetz , E. Chevet , and R. Pedeux , “When Endoplasmic Reticulum Proteostasis Meets the DNA Damage Response,” Trends in Cell Biology 30, no. 11 (2020): 881–891, 10.1016/j.tcb.2020.09.002. 33036871

[53] O. Chatzidoukaki , E. Goulielmaki , B. Schumacher , and G. A. Garinis , “DNA Damage Response and Metabolic Reprogramming in Health and Disease,” Trends in Genetics 36, no. 10 (2020): 777–791, 10.1016/j.tig.2020.06.018. 32684438

[54] L. Richmond and K. Keeshan , “Pseudokinases: A Tribble‐edged Sword,” The FEBS Journal 287, no. 19 (2020): 4170, 10.1111/febs.15096. 31621188

[55] D. Mondal , A. Mathur , and P. K. Chandra , “Tripping on TRIB3 at the Junction of Health, Metabolic Dysfunction and Cancer,” Biochimie 124 (2016): 34–52, 10.1016/j.biochi.2016.02.005. 26855171

[56] X. Ren , N. Chen , Y. Chen , W. Liu , and Y. Hu , “TRB3 stimulates SIRT1 Degradation and Induces Insulin Resistance by Lipotoxicity via COP1,” Experimental Cell Research 382, no. 1 (2019): 111428, 10.1016/j.yexcr.2019.05.009. 31125554

[57] T. Wang , D. Rao , C. Fu , et al., “MET Promotes Hepatocellular Carcinoma Development Through the Promotion of TRIB3‐mediated FOXO1 Degradation,” Clinical and Molecular Hepatology 31, no. 3 (2025): 1032, 10.3350/cmh.2024.1163. 40211872 PMC12260640

[58] J. J. Yu , D. D. Zhou , X. X. Yang , et al., “TRIB3‐EGFR Interaction Promotes Lung Cancer Progression and Defines a Therapeutic Target,” Nature Communications 11, no. 1 (2020): 3660, 10.1038/s41467-020-17385-0. PMC7374170 32694521

[59] J.‐M. Yu , W. Sun , Z.‐H. Wang , et al., “TRIB3 Supports Breast Cancer Stemness by Suppressing FOXO1 Degradation and Enhancing SOX2 Transcription,” Nature Communications 10, no. 1 (2019): 5720, 10.1038/s41467-019-13700-6. PMC6915745 31844113

[60] J. Roška , L. Wachsmannová , L. Hurbanová , et al., “Differential Gene Expression in Cisplatin‐Resistant and ‐Sensitive Testicular Germ Cell Tumor Cell Lines,” Oncotarget 11, no. 51 (2020): 4735, 10.18632/oncotarget.27844. 33473258 PMC7771712

[61] H. Bolland , T. S. Ma , S. Ramlee , K. Ramadan , and E. M. Hammond , “Links Between the Unfolded Protein Response and the DNA Damage Response in Hypoxia: A Systematic Review,” Biochemical Society Transactions 49, no. 3 (2021): 1251–1263, 10.1042/bst20200861. 34003246 PMC8286837

[62] L. Kong , L. Kong , P. Li , et al., “Tribbles Pseudokinase 3 Promoted Renal Fibrosis by Regulating the Expression of DNA Damage‐inducible Transcript 3 in Diabetic Nephropathy,” Biomolecules and Biomedicine 24, no. 6 (2024): 1559, 10.17305/bb.2024.10419. 38733632 PMC11496876

[63] B. Qian , H. Wang , X. Men , et al., “TRIB3 is Implicated in Glucotoxicity‐ and Oestrogen Receptor‐Stress‐Induced β‐cell Apoptosis,” Journal of Endocrinology 199, no. 3 (2008): 407–416, 10.1677/joe-08-0331. 18818302

[64] Y. C. Lee , W. L. Wang , W. C. Chang , et al., “Tribbles Homolog 3 Involved in Radiation Response of Triple Negative Breast Cancer Cells by Regulating Notch1 Activation,” Cancers (Basel) 11, no. 2 (2019): 127, 10.3390/cancers11020127. 30678233 PMC6406679

[65] J. Sun , G. Wu , F. Pastor , et al., “RNA Helicase DDX5 Enables STAT1 mRNA Translation and Interferon Signalling in hepatitis B Virus Replicating Hepatocytes,” Gut 71, no. 5 (2022): 991, 10.1136/gutjnl-2020-323126. 34021034 PMC8606016

[66] S. Y. Mersaoui , Z. Yu , Y. Coulombe , et al., “Arginine Methylation of the DDX5 Helicase RGG/RG Motif by PRMT5 Regulates Resolution of RNA:DNA Hybrids,” The EMBO Journal 38, no. 15 (2019): 100986, 10.15252/embj.2018100986. PMC6669924 31267554

[67] Q. Liu , M. Han , Z. Wu , et al., “DDX5 Inhibits Hyaline Cartilage Fibrosis and Degradation in Osteoarthritis via Alternative Splicing and G‐quadruplex Unwinding,” Nature Aging 4, no. 5 (2024): 664–680, 10.1038/s43587-024-00624-0. 38760576 PMC11108786

[68] Z. Szeltner , G. Ferenc , T. Juhász , et al., “Probing Telomeric‐Like G4 Structures With Full or Partial 2'‐deoxy‐5‐hydroxyuridine Substitutions,” Biochimie 214, no. Pt A (2023): 33–44, 10.1016/j.biochi.2023.01.009. 36707016

[69] A. Berner , R. N. Das , N. Bhuma , et al., “G4‐Ligand‐Conjugated Oligonucleotides Mediate Selective Binding and Stabilization of Individual G4 DNA Structures,” Journal of the American Chemical Society 146, no. 10 (2024): 6926–6935, 10.1021/jacs.3c14408. 38430200 PMC10941181

[70] C. Kumar , S. Batra , J. D. Griffith , and D. Remus , “The Interplay of RNA:DNA Hybrid Structure and G‐quadruplexes Determines the Outcome of R‐Loop‐Replisome Collisions,” Elife 10 (2021), 10.7554/eLife.72286. PMC8479836 34494544

[71] R. S. Finn , S. Qin , M. Ikeda , et al., “Atezolizumab plus Bevacizumab in Unresectable Hepatocellular Carcinoma,” New England Journal of Medicine 382, no. 20 (2020): 1894–1905, 10.1056/NEJMoa1915745. 32402160

[72] G. K. Abou‐Alfa , G. Lau , M. Kudo , et al., “Tremelimumab plus Durvalumab in Unresectable Hepatocellular Carcinoma,” NEJM Evidence 1, no. 8 (2022): EVIDoa2100070, 10.1056/EVIDoa2100070. 38319892

[73] S. Cappuyns , V. Corbett , M. Yarchoan , R. S. Finn , and J. M. Llovet , “Critical Appraisal of Guideline Recommendations on Systemic Therapies for Advanced Hepatocellular Carcinoma: A Review,” JAMA Oncology 10, no. 3 (2024): 395–404, 10.1001/jamaoncol.2023.2677. 37535375 PMC10837331

[74] R. Q. Wang , F. Z. He , Q. Meng , et al., “Tribbles Pseudokinase 3 (TRIB3) Contributes to the Progression of Hepatocellular Carcinoma by Activating the Mitogen‐activated Protein Kinase Pathway,” Annals of Translational Medicine 9, no. 15 (2021): 1253, 10.21037/atm-21-2820. 34532390 PMC8421934

[75] J. H. Han and J. Huang , “DNA Double‐strand Break Repair Pathway Choice: The Fork in the Road,” Genome Instability & Disease 1, no. 1 (2019): 10.

[76] Y. D. Liu and L. Y. Lu , “BRCA1: A Key Player at Multiple Stages of Homologous Recombination in DNA Double‐strand Break Repair,” Genome Instability & Disease 2, no. 3 (2021): 164.

[77] R. Hänsel‐Hertsch , D. Beraldi , S. V. Lensing , et al., “G‐quadruplex Structures Mark human Regulatory Chromatin,” Nature Genetics 48, no. 10 (2016): 1267–1272, 10.1038/ng.3662. 27618450

[78] A. Alagia , R. F. Ketley , and M. Gullerova , “Proximity Ligation Assay for Detection of R‐Loop Complexes Upon DNA Damage,” Methods in Molecular Biology 2528 (2022): 289–303, 10.1007/978-1-0716-2477-7_19. 35704199

