# Supplementary material for: Glutamine Deprivation Triggers Tribbles Homolog 3 Dependent G‐Quadruplex Resolution to Maintain DNA Repair and Tumor Survival
Source: Adv Sci (Weinh). 2026 Mar 7;13(27):e20798. doi: 10.1002/advs.202520798 (PMC13170200; doi:10.1002/advs.202520798)
Supplement: Supplementary file 1 — Supporting File: advs74697‐sup‐0001‐SuppMat.docx. [file ADVS-13-e20798-s001.docx]

# Supplementary Information

Glutamine Deprivation Triggers Tribbles Homolog 3 Dependent G-quadruplex Resolution to Maintain DNA Repair and Tumor Survival

Qiang Ji^1,#^, Xuedan Sun^2,#^, Zhangran Sun^1^, Mengfan Li^1^, Xinyu Cheng^3^, Shuai Tian^1^, Rick F. Thorne^1^, Jinming Li^1^, Guangzhi Liu^4,✉^, Mian Wu^1,✉^, Xiaoying Liu^1,3,✉^

^1^ Translational Research Institute of Henan Provincial People’s Hospital and School of Basic Medical Sciences, Henan University, Zhengzhou, 450046, China.

^2^ Department of Hepatobiliary Surgery, Centre for Leading Medicine and Advanced Technologies of IHM, The First Affiliated Hospital of USTC, Division of Life Sciences and Medicine, University of Science and Technology of China, Hefei, 230032, China.

^3^ School of Life Sciences, Anhui Medical University, Hefei, 230032, China.

^4^ Henan Key Laboratory of Stem cell Differentiation and Modification, Henan Provincial People’s Hospital, Zhengzhou, Henan 450053, China.

^#^ These authors contributed equally to the work.

*To whom correspondence should be addressed: Guangzhi Liu, Mian Wu or Xiaoying Liu

E-mail: [guangzhi72](mailto:huangshoujun@ahau.edu.cn)@126.com, wumian@ustc.edu.cn or [liuxiaoying@ahmu.edu.cn](mailto:liuxiaoying@ahmu.edu.cn)

# Supplementary Figures


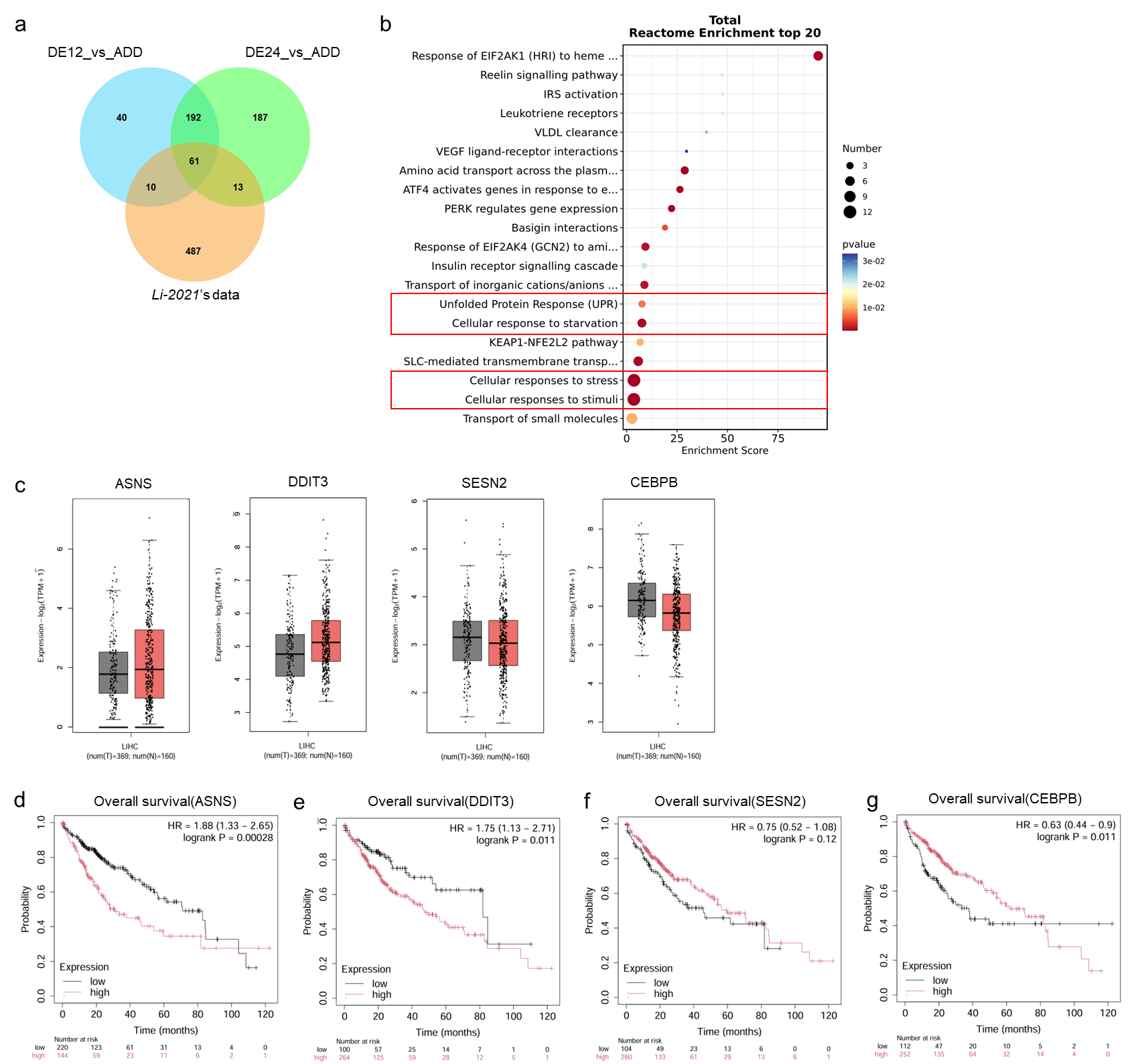


**Figure S1. Identification of nutrient stress-related genes in HCC (related to Figure 2).**

(**a, b**) Venn diagram intersection of comparing DEGs (Log2FC>2, P<0.01) identified by RNA-seq analysis of Gln-deprived HepG2 cells at 12 h (DE12) and 24 h (DE24) compared with glucose restriction for 48 h [1]. (b) The top 20 Reactome pathways enrichments from the 61 overlapping genes identified in (a).

(**c**) Expression of TRIB3, ASNS, DDIT3, SESN2 and CEBPB mRNA in liver hepatocellular carcinoma compared to normal liver tissue. The TCGA LIHC and normal liver dataset was interrogated using Gepia2 (c).

(**d**-**g**) Overall survival (OS) of liver cancer patients whose tumors were stratified by ASNS (d), DDIT3 (e), SESN2 (f), and CEBPB (g) expression. Plots were obtained using Kaplan-Meier Plotter (https://kmplot.com). Numbers at risk are indicated below each plot.

Data information: (c) values are median, first and third quartile. n = 369 (Tumor group), 160 (Normal group). (d-g) n = 364 patients.


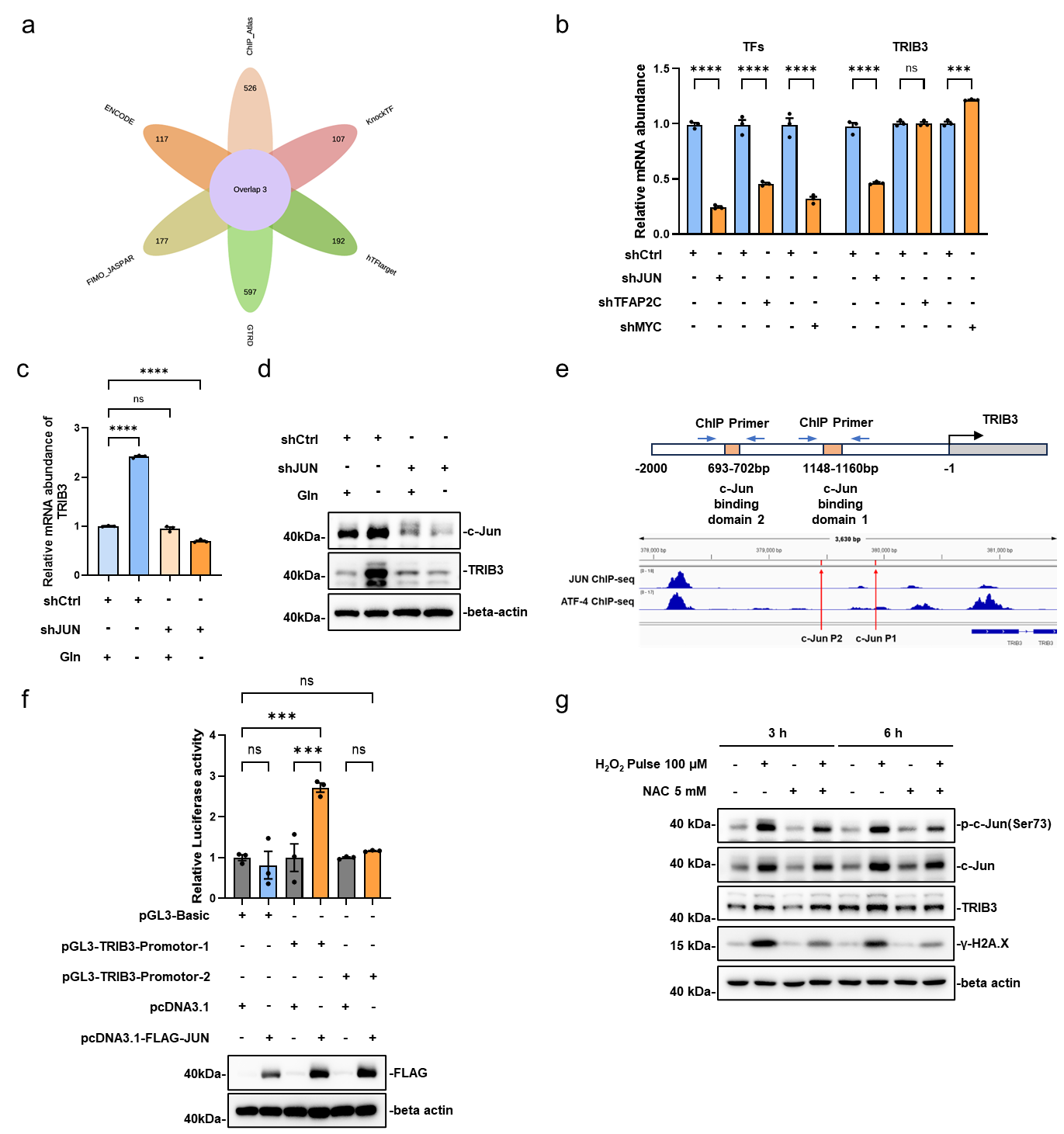


**Figure S2. c-Jun transcriptionally activates TRIB3.**

**(a)** Venn diagram illustrating the intersection of six algorithmic predictions (ENCODE, ChIP_Atlas, KnockTF, hTFtarget, GTRD, FIMO_Jaspar) of transcription factors targeting the TRIB3 promoter, identified using TF Target Finder (https://jingle.shinyapps.io/TF_Target_Finder/).

**(b)** HepG2 cells were transiently transfected with a control shRNA vector (shCtrl) or shRNAs targeting each of the three common transcription factors identified in (a; JUN, TFAP2C, MYC). Their respective transcript levels together with TRIB3 mRNA were measured using qPCR.

(**c, d**) HepG2 cells transfected with shCtrl or shJUN were cultured with or without Gln deprivation for 12 hours before measuring TRIB3 mRNA expression by qPCR (c); TRIB3 and c-Jun protein levels were measured by Western blotting (d).

(**e**) (top) Schematic diagram showing c-Jun binding sites and ChIP primer locations within the TRIB3 promoter region predicted by JASPAR database analysis, (bottom) IGV snapshot of the TRIB3 locus (chr20: 377,866–381,495; GRCh38) displaying ENCODE HepG2 JUN/c-Jun ChIP-seq signal (GEO: GSM935364; bigWig: ENCFF732BEG) and an ENCODE HepG2 ATF4 ChIP-seq track (GEO: GSE175148; bigwig: ENCFF450YCE). Red arrows indicate the locations of the c-Jun ChIP-qPCR amplicons P1 and P2 used in this study.

(**f**) Dual-luciferase reporter assays undertaken in HepG2 cells after expression of the indicated pGL3 promoter constructs, with and without c-Jun overexpression.

(**g**) Western blotting analysis of HepG2 cells pretreated with N-acetyl-L-cysteine (NAC, 5 mM) for 1 h, followed by a H₂O₂ (100 μM, 1 h) in the continued presence of NAC. Cells were cultured in fresh medium containing NAC without H₂O₂, for an additional 3 h or 6 h before analysis.

Data information: (b, c, f) values are mean ± SEM, n=3 biological replicates; (b) two-way ANOVA with Turkey’s multiple comparisons test; (c, f) one-way ANOVA with Turkey’s multiple comparison test; (i) two-tailed unpaired t test. ns, not signif,icant, ***, P < 0.001, **** P < 0.0001.


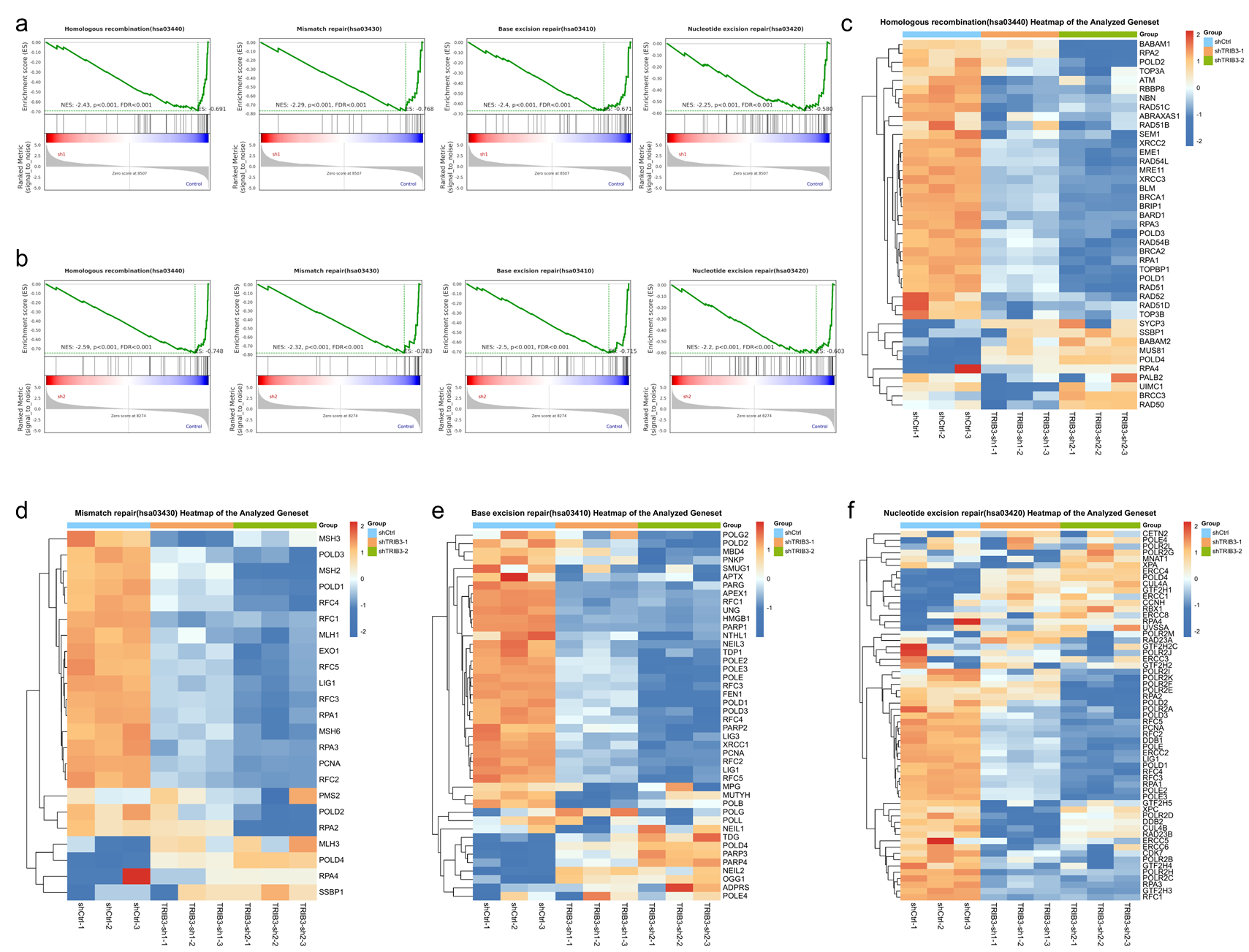


**Figure S3. Gene set enrichment analysis (GSEA) of RNA-seq data (related to Figure 3).**

(**a**) Gene set enrichment analysis (GSEA) from the RNA-seq data of HepG2 cells with shTRIB3-1 knockdown showing significant downregulation of HR (NES = -2.43, p<0.001, FDR<0.001), MMR (NES = -2.29, p<0.001, FDR<0.001), BER (NES = -2.4, p<0.001, FDR<0.001), and NER (NES= -2.25 p<0.001, FDR<0.001) pathway genes.

(**b**) GSEA from the RNA-seq data of HepG2 cells with shTRIB3-2-knockdown showing distinct downregulation of genes involved in HR (NES = -2.59, p<0.001, FDR<0.001), MMR (NES = -2.32, p<0.001, FDR<0.001), BER (NES = -2.5, p<0.001, FDR<0.001), and NER (NES = -2.2, p<0.001, FDR<0.001) DNA repair pathways.

(**c-f**) The corresponding heatmaps confirmed highly concordant gene expression changes between the two independent TRIB3 knockdown sequences.


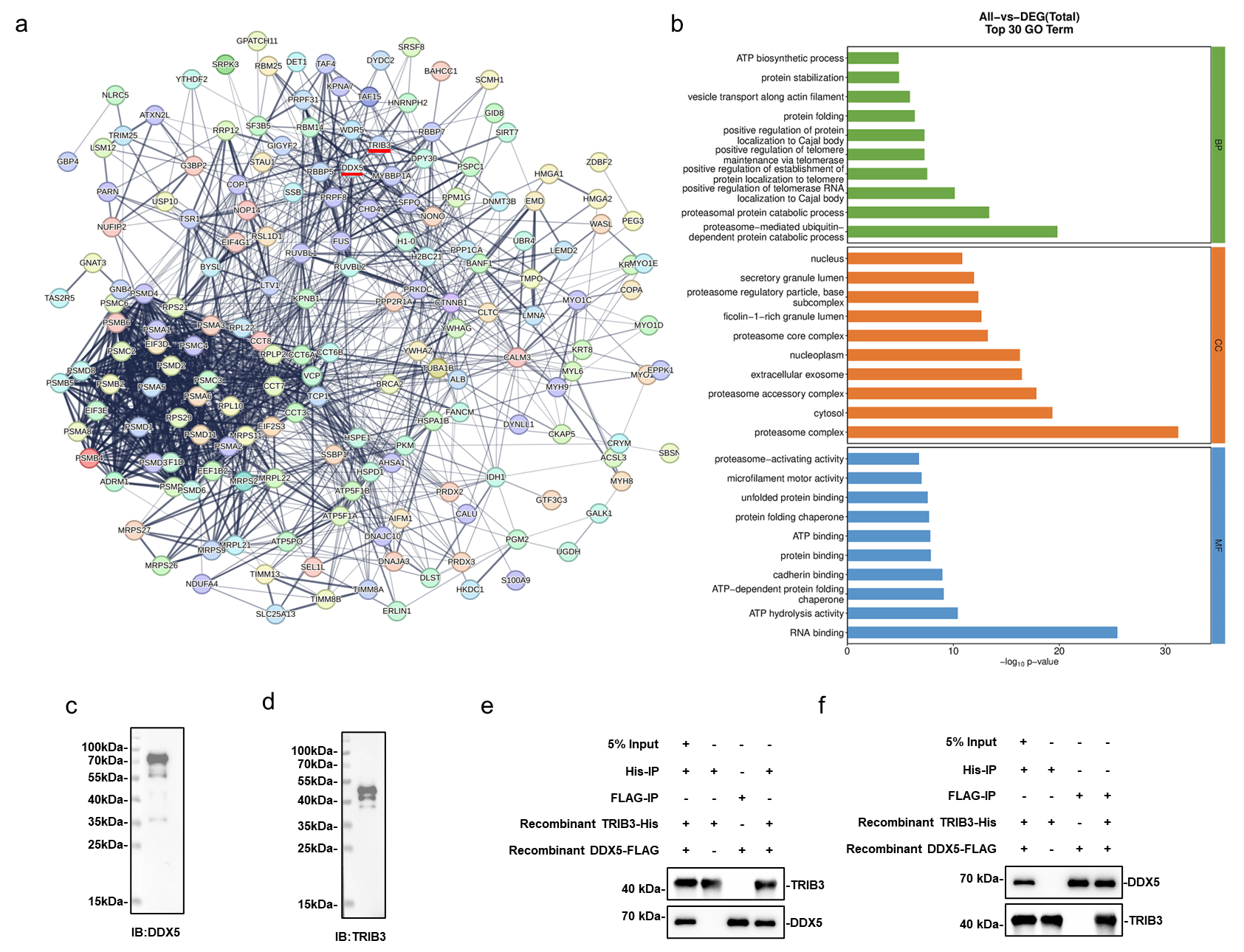


**Figure S4. Network and functional annotation of TRIB3 interactome and validation of direct TRIB3–DDX5 binding *in vitro*.**

(**a**) The String database was used to construct a PPI network based on the 202 candidate TRIB3-binding proteins identified in LC-MS analysis (Figure 4a) to shortlist potential candidates for further analysis.

(**b**) Gene Ontology (GO) enrichment of the 202 candidate TRIB3-binding proteins identified in LC-MS analysis (Figure 4a).

(**c, d**) Verification of recombinant purified DDX5-FLAG (d) and TRIB3-His (e) proteins using Western blotting against specific antibodies to DDX5 and TRIB3.

(**e, f**) Reciprocal co-immunoprecipitation analyses were performed between the recombinant DDX5-FLAG and TRIB3-His proteins from (c, d). DDX5 was recovered using TRIB3-His (e) and TRIB3 was recovered using DDX5-FLAG as bait. Western blotting was used to detect the DDX5 and TRIB3 proteins with 5 % input shown as a loading control.


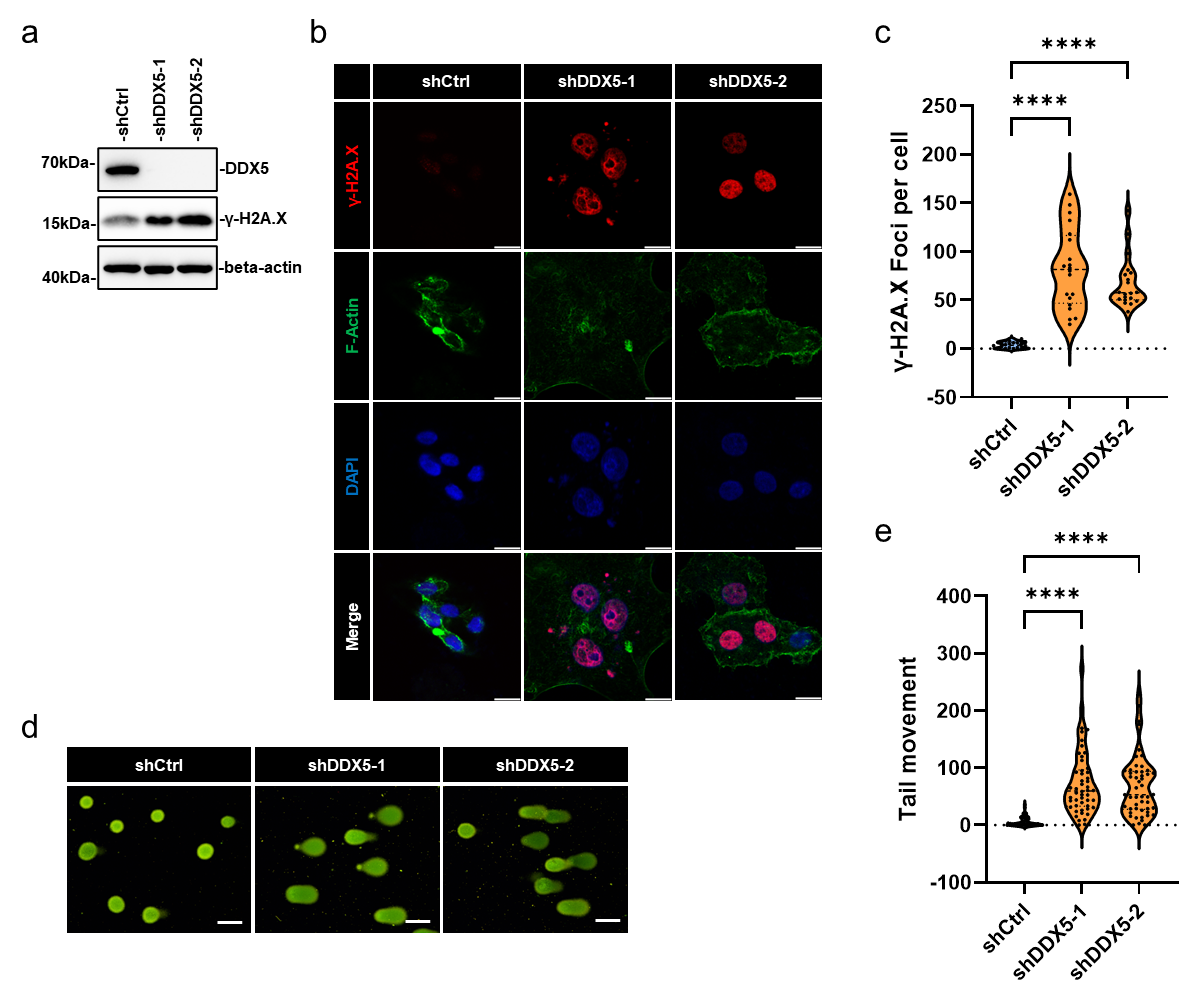


**Figure S5. Knockdown of DDX5 induces DNA damage.**

(**a**) The effect of DDX5 shRNA knockdown on γ-H2A.X (p-Ser139) expression in HepG2 cells determined by Western blotting.

(**b, c**) Representative confocal images showing γ-H2A.X (red), F-actin (green), and DAPI (blue) staining in HepG2 cells with or without DDX5 knockdown (scale bars, 10 μm) (c); quantification of γ-H2A.X foci per cell (c).

(**d, e**) Representative images of alkaline comet assay in HepG2 cells following DDX5 knockdown (scale bars, 10 μm) (d); quantification of tail movement (e).

Data information: (c, e) values are median, first and third quartile; (c) n=20, (e) n=50; (c, e) one-way ANOVA with Turkey’s multiple comparison test. **** P < 0.0001.


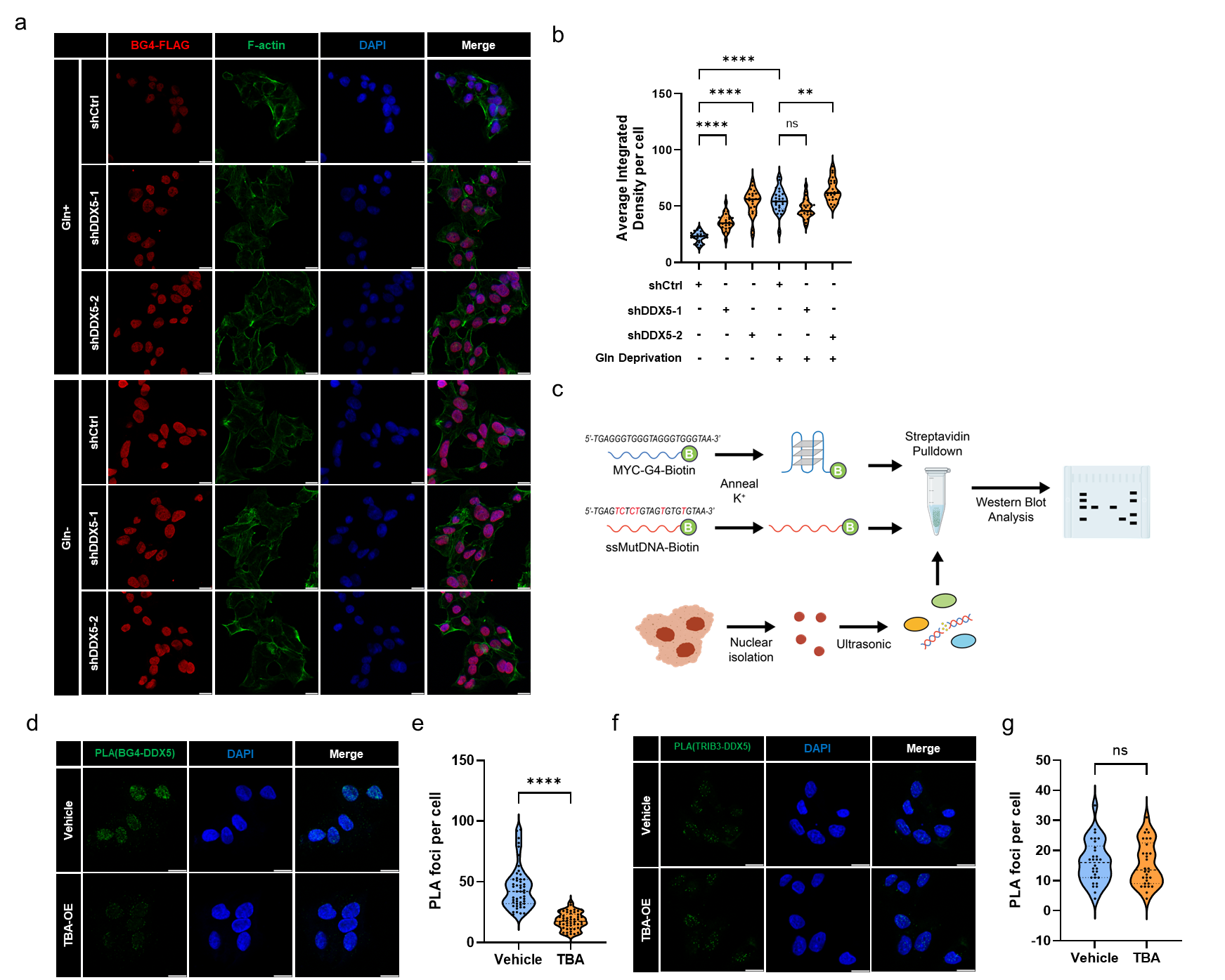


**Figure S6. Effects of TRIB3/DDX5 knockdown on G4 genomic occupancy.**

(**a, b**) Representative confocal images of BG4 (red), F-actin (green), and DAPI (blue) staining in HepG2 cells with DDX5 knockdown in combination with Gln deprivation (scale bars, 10 μm) (b); quantification of average integrated BG4 density per cell (c).

(**c**) G4-DNA pulldown assay. Biotinylated G4-DNA probes are annealed and incubated with HepG2 cell nuclear extracts prepared by sonication. Thereafter, the probes and binding proteins were recovered with streptavidin beads and the samples subject to Western blotting analysis. The G4-MYC probe is shown as an example.

(**d-g**) PLA assays undertaken between BG4-DDX5 (d, e) and TRIB3-DDX5 (f, g) in HepG2 cells transfected without (vehicle) or with 100 nM of G4 decoy TBA (thrombin-binding aptamer; 5′-GGTTGGTGTGGTTGG-3’; TBA-OE). Representative PLA images showing interaction foci (green) and DAPI nuclear staining (blue) (scale bars, 10 μm). (d, f). Quantification of interaction foci per cell (e, g).

Data information: (b) values are median, first and third quartile, (b) n=24, (e) n=50, (g) n=30; (b) one-way ANOVA with Turkey’s multiple comparison test, (e, g) two-tailed unpaired t test. ns, not significant, ** P< 0.01 **** P < 0.0001.


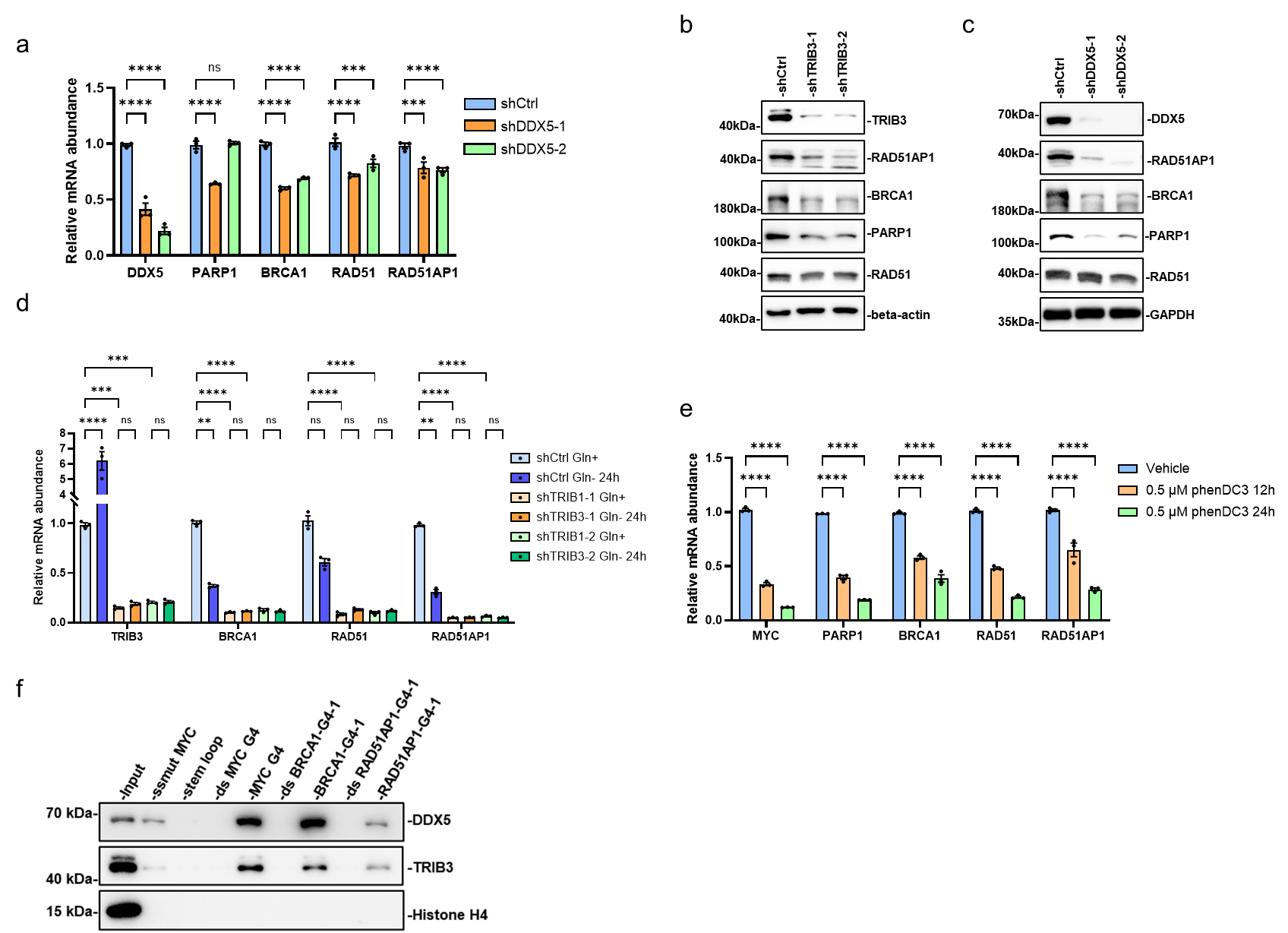


**Figure S7. Analysis of HR gene expression following knockdown of TRIB/DDX5.**

**(a)** Relative mRNA expression of HR-related genes in HepG2 cells with or without DDX5 shRNA knockdown determined by qPCR.

**(b, c)** HepG2 cells subject to knockdown of TRIB3 (b) or DDX5 (c) and the expression of HR-related proteins determined by Western blotting.

**(d)** Relative mRNA expression of TRIB3 and HR pathway related genes in HepG2 cells with or without TRIB3 knockdown and under basal conditions or Gln deprivation conditions, as determined by qPCR.

**(e)** Relative mRNA expression levels of TRIB3 and the HR-related genes in HepG2 cells after treatment with PhenDC3 (0.5 μM) for 0, 12, 24 h. MYC served as a positive control.

**(f)** Biotinylated oligonucleotide probes corresponding to MYC-G4, BRCA1-G4-1, and RAD51AP1-G4-1 (Figure 7) were used to generate matched G4, stem-loop, and double-stranded DNA (dsDNA) structural controls. Prior to incubation with lysates, all probes were folded by annealing in anneal buffer. Probes were then incubated with HepG2 cell lysates followed by streptavidin-affinity pull-down, and TRIB3, DDX5 and Histone H4 were analyzed by Western blotting.

Data information: (a, d, e) values are mean ± SEM, n=3 biological replicates; (a, d, e) one-way ANOVA with Turkey’s multiple comparison test. ns, not significant, ** P < 0.01, *** P < 0.001, **** P < 0.0001.


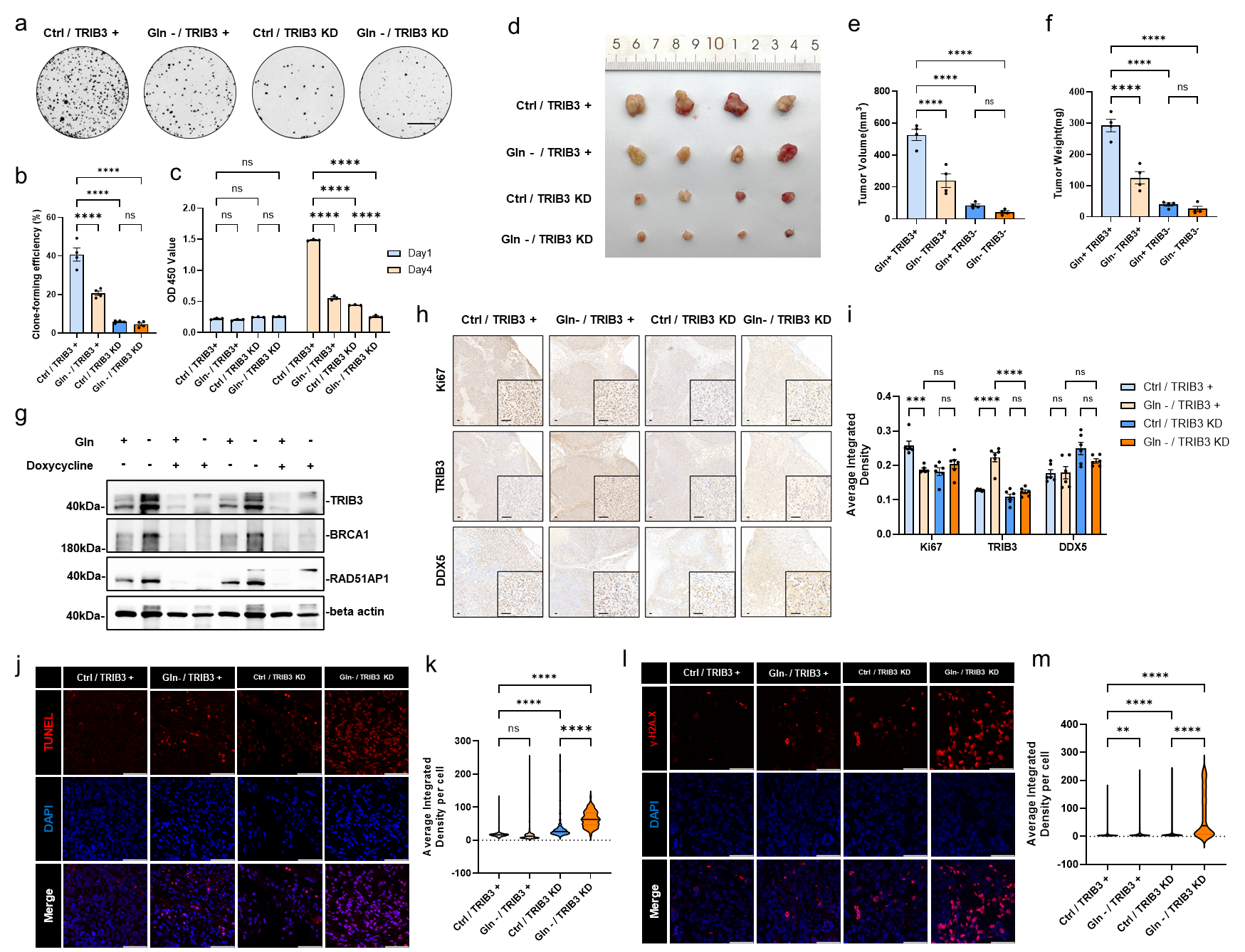


**Figure S8. TRIB3 knockdown in combination with Gln-deficient diet suppresses tumor growth in nude mouse xenografts.**

(**a, b**) Representative images of colony formation assays in HepG2 cells after 14 d under control (Ctrl) and glutamine deprivation (Gln-) conditions and without (TRIB+) and with TRIB3 (TRIB3 KD) knockdown (scale bars, 1 cm) (a); quantification of clone-forming efficiency ratios (b).

(**c**) Cell proliferation measurements of HepG2 cells as per (a) at 1 and 4 d determined using the Cell viability counting kit 8 (CCK-8) assay.

(**d-f**) Doxycycline (Dox)-inducible TRIB3 knockdown and control isogenic Huh7 cell sublines were used to establish subcutaneous xenografts in nude mice before inducing knockdown with Dox-supplemented water. Subgroups of mice received either a normal (Ctrl) or glutamine-deficient diet (Gln-) for a further 3 weeks. Images of the excised tumors (d), tumor volume (mm^3^) (e), tumor weight (mg) (f) mouse xenografts.

**(j-m)** Tumor sections from (d-f) were subject to TUNEL staining to measure apoptosis (j, k) or γ-H2A.X staining to measure DNA damage (l, m). Representative confocal images of TUNEL staining with nuclei decorated by DAPI (blue) (red) (scale bars, 10 μm) (j); quantification of average integrated density of TUNEL signals per cell (k). Representative confocal images of γ-H2A.X staining with nuclei decorated by DAPI (blue) (red) (scale bars, 10 μm) (l); quantification of average integrated density of γ-H2A.X signals per cell (m).

Data information: (b, c, e, f, i) values are mean ± SEM, (k, m) values are median, first and third quartile; (b) n=4 biological replicates; (c) n=3 biological replicates; (e, f) n=4, (i) n=6, (k) n>300, (m) n>540; (b, c, e, f, i, k, m) one-way ANOVA with Turkey’s multiple comparison test. ns, not significant, * P< 0.05, *** P < 0.001, **** P < 0.0001.

References：

1. Li, M., Thorne, R. F., Shi, R., et al., "DDIT3 Directs a Dual Mechanism to Balance Glycolysis and Oxidative Phosphorylation during Glutamine Deprivation," *Adv Sci (Weinh)* 2021, 8 (11), e2003732, <https://doi.org/10.1002/advs.202003732>.

# Supplementary Tables

**Supplementary Table S1**. Unique protein identifies of TRIB3-interactors identified from LC-MS analysis, related to Figure 4.

| **Header** | **Accession** | **Protein FDR Confidence: Combined** | **Sum PEP Score** | **Coverage [%]** | **# Peptides** | **# PSMs** | **# Unique Peptides** | **Abundances (Normalized): F2: Sample** | **Modifications** |
| --- | --- | --- | --- | --- | --- | --- | --- | --- | --- |
| HSPD1 | P10809 | High | 328.729 | 76 | 41 | 123 | 41 | 13313044419 |  |
| TRIB3 | Q96RU7 | High | 282.751 | 73 | 26 | 145 | 26 | 42048251529 |  |
| TUBA1B | P68363 | High | 151.688 | 67 | 21 | 57 | 3 | 8457642214 |  |
| PARN | O95453 | High | 117.069 | 46 | 27 | 48 | 27 | 2740827165 |  |
| KRT8 | P05787 | High | 113.563 | 53 | 28 | 44 | 22 | 2116952744 |  |
| HSPA1B | P0DMV9 | High | 65.512 | 35 | 20 | 24 | 18 | 926848160.5 |  |
| NONO | Q15233 | High | 63.856 | 39 | 19 | 27 | 17 | 1067859031 |  |
| H4C1; H4C11; H4C12; H4C13; H4C14; H4C15; H4C16; H4C2; H4C3; H4C4; H4C5; H4C6; H4C8; H4C9 | P62805 | High | 62.727 | 54 | 10 | 29 | 10 | 5534445478 |  |
| SFPQ | P23246 | High | 52.424 | 33 | 19 | 24 | 18 | 1151815254 |  |
| DDX5 | P17844 | High | 47.525 | 28 | 16 | 17 | 11 | 1789298142 |  |
| RPLP2 | P05387 | High | 42.2 | 80 | 6 | 12 | 6 | 660906849 |  |
| H2AC25 | Q7L7L0 | High | 34.924 | 35 | 6 | 14 | 2 | 2746280898 |  |
| HNRNPH2 | P55795 | High | 34.515 | 16 | 6 | 14 | 1 | 16950108 |  |
| H2BC21 | Q16778 | High | 34.426 | 36 | 4 | 13 | 1 | 3013079992 |  |
| ATP5F1A | P25705 | High | 34.114 | 22 | 10 | 10 | 10 | 336471304 |  |
| MYH9 | P35579 | High | 33.541 | 9 | 12 | 12 | 12 | 315417347 |  |
| PSMD2 | Q13200 | High | 33.278 | 13 | 8 | 9 | 8 | 139975562.3 |  |
| SLC25A13 | Q9UJS0 | High | 32.994 | 23 | 12 | 13 | 12 | 272332236 |  |
| CCT3 | P49368 | High | 29.134 | 26 | 10 | 12 | 10 | 455249506 |  |
| COP1 | Q8NHY2 | High | 28.064 | 16 | 8 | 8 | 8 | 347139834 |  |
| G3BP2 | Q9UN86 | High | 24.784 | 24 | 7 | 8 | 7 | 174615511 |  |
| ATP5F1B | P06576 | High | 24.73 | 19 | 7 | 7 | 7 | 167373333 |  |
| MYO1C | O00159 | High | 23.353 | 10 | 8 | 9 | 8 | 177982229 |  |
| RPL22 | P35268 | High | 23.041 | 41 | 5 | 10 | 5 | 73316362630 |  |
| PRPF31 | Q8WWY3 | High | 21.252 | 19 | 7 | 9 | 7 | 1661097972 |  |
| CCT8 | P50990 | High | 21.005 | 13 | 6 | 6 | 6 | 203164759 |  |
| RRP12 | Q5JTH9 | High | 20.958 | 8 | 8 | 8 | 8 | 125390596 |  |
| CALM3 | P0DP25 | High | 20.721 | 19 | 2 | 5 | 2 | 395471774 |  |
| MYCBP | Q99417 | High | 20.374 | 67 | 6 | 6 | 6 | 379409091 |  |
| SSB | P05455 | High | 17.995 | 15 | 5 | 6 | 5 | 183742708 |  |
| CCT6A | P40227 | High | 17.081 | 17 | 6 | 6 | 5 | 148778780 |  |
| CLTC | Q00610 | High | 16.186 | 6 | 7 | 8 | 7 | 205588332 |  |
| FUS | P35637 | High | 15.504 | 10 | 3 | 4 | 2 | 134837932 |  |
| PSMC4 | P43686 | High | 15.475 | 20 | 7 | 7 | 7 | 124847958 | Acetyl [N-Term] |
| ATXN2L | Q8WWM7 | High | 15.045 | 6 | 5 | 6 | 5 | 107250372 |  |
| PEG3 | Q9GZU2 | High | 14.952 | 5 | 5 | 5 | 5 | 192551069 |  |
| KRT15 | P19012 | High | 14.24 | 10 | 5 | 8 | 1 | 7458690 |  |
| TIMM13 | Q9Y5L4 | High | 13.18 | 53 | 4 | 4 | 4 | 375975353.5 |  |
| LMNA | P02545 | High | 13.119 | 6 | 3 | 4 | 3 | 65410410.5 |  |
| PSPC1 | Q8WXF1 | High | 12.202 | 11 | 5 | 6 | 4 | 88014462.5 |  |
| ERLIN1 | O75477 | High | 11.513 | 17 | 5 | 5 | 3 | 119743973 |  |
| WDR5 | P61964 | High | 11.377 | 19 | 4 | 5 | 4 | 106293285 |  |
| PSMD1 | Q99460 | High | 11.161 | 3 | 3 | 3 | 3 | 53267734 |  |
| PSMD3 | O43242 | High | 11.15 | 12 | 5 | 5 | 5 | 62525382 |  |
| HSPE1 | P61604 | High | 10.505 | 33 | 3 | 4 | 3 | 124908848 |  |
| CCT7 | Q99832 | High | 10.32 | 13 | 4 | 4 | 4 | 34779755.5 |  |
| H3-3A; H3-3B | P84243 | High | 10.025 | 20 | 4 | 11 | 2 | 1772741424 |  |
| RBM14 | Q96PK6 | High | 9.604 | 6 | 2 | 2 | 2 | 19703164 |  |
| TAF15 | Q92804 | High | 9.432 | 5 | 2 | 2 | 1 | 29662176 |  |
| HMGA1 | P17096 | High | 9.39 | 23 | 3 | 4 | 3 | 132560115 |  |
| PSMD7 | P51665 | High | 9.363 | 9 | 2 | 3 | 2 | 50288369 |  |
| BYSL | Q13895 | High | 9.354 | 12 | 4 | 4 | 4 | 59876846 |  |
| EMD | P50402 | High | 8.756 | 15 | 3 | 3 | 3 | 45452655 |  |
| PSMB6 | P28072 | High | 8.669 | 13 | 3 | 3 | 3 | 82159746 |  |
| TCP1 | P17987 | High | 8.499 | 9 | 3 | 3 | 3 | 84796926 |  |
| RBBP5 | Q15291 | High | 8.286 | 6 | 3 | 3 | 3 | 49309270 |  |
| PSMA6 | P60900 | High | 8.225 | 13 | 3 | 3 | 3 | 56024767.5 |  |
| PSMA3 | P25788 | High | 8.217 | 15 | 4 | 4 | 4 | 80725344 |  |
| PSMA5 | P28066 | High | 7.963 | 13 | 2 | 2 | 2 | 62217480 |  |
| PRDX2 | P32119 | High | 7.572 | 15 | 2 | 2 | 1 | 15498492 |  |
| CCT6B | Q92526 | High | 6.989 | 5 | 2 | 2 | 1 | 44239024 |  |
| PSMD6 | Q15008 | High | 6.953 | 12 | 4 | 4 | 4 | 31619152 |  |
| H3-7 | Q5TEC6 | High | 6.631 | 20 | 3 | 8 | 1 | 126023228 |  |
| SSBP1 | Q04837 | High | 6.565 | 14 | 2 | 2 | 2 | 35617766.5 |  |
| SF3B5 | Q9BWJ5 | High | 6.44 | 38 | 2 | 2 | 2 | 77571616 |  |
| HMGA2 | P52926 | High | 6.419 | 33 | 2 | 2 | 2 | 45963866 |  |
| LSM12 | Q3MHD2 | High | 6.414 | 17 | 2 | 2 | 2 | 50226852 |  |
| TMPO | P42166 | High | 6.39 | 5 | 2 | 2 | 2 | 41275220 |  |
| NOP14 | P78316 | High | 6.201 | 2 | 1 | 1 | 1 | 77525320 |  |
| EIF2S3 | P41091 | High | 6.149 | 6 | 2 | 2 | 2 | 29664032 |  |
| PRPF8 | Q6P2Q9 | High | 6.035 | 1 | 3 | 3 | 3 | 18201231.5 |  |
| A1CF | Q9NQ94 | High | 5.781 | 6 | 2 | 2 | 2 | 295993464 |  |
| UGDH | O60701 | High | 5.561 | 5 | 2 | 2 | 2 | 23077950 |  |
| RUVBL1 | Q9Y265 | High | 5.528 | 3 | 1 | 1 | 1 | 17081484 |  |
| PSMA8 | Q8TAA3 | High | 5.046 | 5 | 1 | 1 | 1 | 25441922 |  |
| UBR4 | Q5T4S7 | High | 4.954 | 1 | 2 | 2 | 2 | 8265047.75 |  |
| PSMB5 | P28074 | High | 4.945 | 5 | 1 | 1 | 1 | 31809852 |  |
| RPL10 | P27635 | High | 4.781 | 23 | 3 | 3 | 3 | 30898561 |  |
| ALB | P02768 | High | 4.576 | 3 | 2 | 2 | 2 | 275784824 |  |
| EIF4G1 | Q04637 | High | 4.554 | 1 | 2 | 2 | 2 | 36949923.5 |  |
| PSMD8 | P48556 | High | 4.445 | 9 | 3 | 3 | 3 | 51194459 |  |
| YWHAG | P61981 | High | 4.21 | 9 | 2 | 2 | 1 | 14872885 |  |
| BANF1 | O75531 | High | 4.131 | 13 | 1 | 1 | 1 | 35941744 |  |
| S100A9 | P06702 | High | 4.124 | 13 | 1 | 1 | 1 | 11876401 |  |
| PSMC2 | P35998 | High | 4.085 | 5 | 2 | 2 | 2 | 30662771 |  |
| PSMD11 | O00231 | High | 4.08 | 3 | 1 | 1 | 1 | 49959252 |  |
| TIMM8A | O60220 | High | 3.969 | 11 | 1 | 1 | 1 | 55590504 |  |
| IDH1 | O75874 | High | 3.962 | 3 | 1 | 1 | 1 | 5368311.5 |  |
| MRPS2 | Q9Y399 | High | 3.8 | 7 | 2 | 2 | 2 | 11979390.5 |  |
| MYL6 | P60660 | High | 3.796 | 19 | 2 | 2 | 2 | 72905629 |  |
| VCP | P55072 | High | 3.76 | 1 | 1 | 1 | 1 | 3037240.25 |  |
| TAF4 | O00268 | High | 3.584 | 1 | 1 | 1 | 1 | 14136238 |  |
| GTF3C3 | Q9Y5Q9 | High | 3.53 | 1 | 1 | 1 | 1 | 5148152.5 |  |
| TIMM8B | Q9Y5J9 | High | 3.519 | 13 | 1 | 1 | 1 | 13860478 |  |
| MRPL22 | Q9NWU5 | High | 3.482 | 11 | 2 | 2 | 2 | 17360934 |  |
| AIFM1 | O95831 | High | 3.412 | 4 | 2 | 2 | 2 | 19466810.5 |  |
| ATP5PO | P48047 | High | 3.37 | 7 | 1 | 1 | 1 | 21414530 |  |
| PSMA2 | P25787 | High | 3.213 | 13 | 2 | 2 | 2 | 15834678.5 |  |
| EEF1D | P29692 | High | 3.175 | 4 | 1 | 1 | 1 | 19524420 |  |
| DYNLL1 | P63167 | High | 3.154 | 13 | 1 | 1 | 1 |  |  |
| GNAT3 | A8MTJ3 | High | 3.098 | 3 | 1 | 1 | 1 | 19778534 |  |
| YWHAZ | P63104 | High | 3.055 | 8 | 2 | 2 | 1 | 53961704 |  |
| DNAJA3 | Q96EY1 | High | 3.024 | 5 | 2 | 2 | 2 | 19813102 |  |
| RBM25 | P49756 | High | 3.006 | 3 | 2 | 2 | 2 | 3869046.75 |  |
| MYO1D | O94832 | High | 3.004 | 1 | 1 | 1 | 1 | 7083184.5 |  |
| GNB4 | Q9HAV0 | High | 2.987 | 3 | 1 | 1 | 1 | 18899492 |  |
| PSMB4 | P28070 | High | 2.967 | 4 | 1 | 1 | 1 | 18848202 |  |
| NDUFA4 | O00483 | High | 2.859 | 12 | 1 | 1 | 1 | 102040576 |  |
| SBSN | Q6UWP8 | High | 2.8 | 6 | 1 | 1 | 1 | 4746797.5 |  |
| PSMC6 | P62333 | High | 2.726 | 3 | 1 | 1 | 1 | 20077554 |  |
| MRPL21 | Q7Z2W9 | High | 2.683 | 9 | 1 | 1 | 1 | 10506362 |  |
| MRPS11 | P82912 | High | 2.659 | 8 | 1 | 1 | 1 |  |  |
| PSMD4 | P55036 | High | 2.65 | 5 | 1 | 1 | 1 | 15786647 |  |
| ADRM1 | Q16186 | High | 2.595 | 4 | 1 | 1 | 1 | 12442635 |  |
| CARHSP1 | Q9Y2V2 | High | 2.593 | 11 | 1 | 1 | 1 | 6976536.5 |  |
| YTHDF2 | Q9Y5A9 | High | 2.585 | 2 | 1 | 1 | 1 | 7627348.5 |  |
| BPIFA1 | Q9NP55 | High | 2.533 | 7 | 1 | 1 | 1 | 12156479 |  |
| MRPS27 | Q92552 | High | 2.503 | 4 | 1 | 1 | 1 | 9888859 |  |
| CKAP5 | Q14008 | High | 2.441 | 1 | 1 | 1 | 1 | 15522999 |  |
| PSMA1 | P25786 | High | 2.402 | 4 | 1 | 1 | 1 | 28242806 |  |
| KPNB1 | Q14974 | High | 2.35 | 2 | 1 | 1 | 1 | 9436348 |  |
| H1-0 | P07305 | High | 2.33 | 5 | 1 | 1 | 1 | 31713194 |  |
| SRPK3 | Q9UPE1 | High | 2.33 | 2 | 1 | 1 | 1 | 19672582 |  |
| DLST | P36957 | High | 2.294 | 2 | 1 | 1 | 1 | 14905887 |  |
| SLX9 | Q9NSI2 | High | 2.291 | 6 | 1 | 1 | 1 | 27262852 |  |
| RBBP7 | Q16576 | High | 2.26 | 2 | 1 | 1 | 1 | 11030514 |  |
| STAU1 | O95793 | High | 2.246 | 2 | 1 | 1 | 1 | 14969092 |  |
| PPM1G | O15355 | High | 2.176 | 4 | 2 | 2 | 2 | 222019093 |  |
| GALK1 | P51570 | High | 2.113 | 3 | 1 | 1 | 1 | 17915334 |  |
| LEMD2 | Q8NC56 | High | 2.06 | 3 | 1 | 1 | 1 | 13479208 |  |
| RUVBL2 | Q9Y230 | High | 2.05 | 2 | 1 | 1 | 1 | 14960095 |  |
| USP10 | Q14694 | High | 2.025 | 2 | 1 | 1 | 1 |  |  |
| AHSA1 | O95433 | High | 2.022 | 7 | 1 | 1 | 1 | 10342700 |  |
| CFAP57 | Q96MR6 | High | 2.005 | 2 | 1 | 13 | 1 | 1877256099 |  |
| SFXN4 | Q6P4A7 | High | 1.952 | 4 | 1 | 1 | 1 | 15002771 |  |
| PPP2R1A | P30153 | High | 1.939 | 2 | 1 | 1 | 1 | 9328552 |  |
| TRIM25 | Q14258 | High | 1.928 | 1 | 1 | 1 | 1 | 8258323 |  |
| SLC39A7 | Q92504 | High | 1.921 | 3 | 1 | 1 | 1 | 8589788 |  |
| NUFIP2 | Q7Z417 | High | 1.912 | 1 | 1 | 1 | 1 | 5335335.5 |  |
| DNAJC10 | Q8IXB1 | High | 1.911 | 1 | 1 | 1 | 1 | 11122261 |  |
| SEL1L | Q9UBV2 | High | 1.909 | 1 | 1 | 1 | 1 | 2907594.75 |  |
| CTNNB1 | P35222 | High | 1.891 | 1 | 1 | 1 | 1 | 6028390.5 |  |
| HKDC1 | Q2TB90 | High | 1.868 | 1 | 1 | 1 | 1 | 28896504 |  |
| SRSF8 | Q9BRL6 | High | 1.831 | 3 | 1 | 1 | 1 | 135411152 |  |
| KPNA7 | A9QM74 | High | 1.805 | 7 | 1 | 1 | 1 | 119344480 |  |
| CALU | O43852 | High | 1.794 | 4 | 1 | 1 | 1 | 17458798 |  |
| BAHCC1 | Q9P281 | High | 1.581 | 1 | 1 | 1 | 1 | 1732033408 |  |
| NLRC5 | Q86WI3 | High | 1.581 | 0 | 1 | 1 | 1 | 19259908 |  |
| WASL | O00401 | High | 1.539 | 2 | 1 | 1 | 1 | 2218599.25 |  |
| ARHGEF5 | Q12774 | High | 1.517 | 2 | 1 | 1 | 1 | 42194752 |  |
| RSL1D1 | O76021 | High | 1.513 | 2 | 1 | 1 | 1 | 7462239 |  |
| COPA | P53621 | High | 1.486 | 1 | 1 | 1 | 1 | 4823255 |  |
| MRPS9 | P82933 | High | 1.448 | 3 | 1 | 1 | 1 | 18823830 |  |
| RPS29 | P62273 | High | 1.407 | 14 | 1 | 1 | 1 | 50291052 |  |
| DYDC2 | Q96IM9 | High | 1.404 | 7 | 1 | 1 | 1 | 56515792 |  |
| MYO15A | Q9UKN7 | High | 1.393 | 0 | 1 | 1 | 1 | 364428480 |  |
| EEF1B2 | P24534 | High | 1.392 | 4 | 1 | 1 | 1 | 13808653 |  |
| RPS21 | P63220 | High | 1.388 | 11 | 1 | 1 | 1 | 13038206 |  |
| PPP1CA | P62136 | High | 1.386 | 3 | 1 | 1 | 1 | 9169152 |  |
| MYBBP1A | Q9BQG0 | High | 1.378 | 1 | 1 | 1 | 1 | 4269058 |  |
| NAPSA | O96009 | High | 1.366 | 4 | 1 | 1 | 1 |  |  |
| SLC4A5 | Q9BY07 | High | 1.346 | 1 | 1 | 1 | 1 | 12798632 |  |
| LTV1 | Q96GA3 | High | 1.312 | 1 | 1 | 1 | 1 | 12641239 |  |
| CFAP157 | Q5JU67 | High | 1.305 | 4 | 1 | 1 | 1 |  |  |
| PHEX | P78562 | High | 1.293 | 3 | 1 | 1 | 1 | 80897584 |  |
| GBP4 | Q96PP9 | High | 1.292 | 2 | 1 | 1 | 1 | 4746040.5 |  |
| CDKL3 | Q8IVW4 | High | 1.284 | 5 | 1 | 1 | 1 | 262621840 |  |
| DPY30 | Q9C005 | High | 1.269 | 16 | 1 | 1 | 1 | 6097314 |  |
| GIGYF2 | Q6Y7W6 | High | 1.264 | 2 | 1 | 1 | 1 | 3778895.5 |  |
| DET1 | Q7L5Y6 | Medium | 1.203 | 3 | 1 | 1 | 1 | 4278955.5 |  |
| PKM | P14618 | Medium | 1.198 | 2 | 1 | 1 | 1 | 3802284 |  |
| EIF3E | P60228 | Medium | 1.192 | 2 | 1 | 1 | 1 | 13629041 |  |
| IGHV3-72 | A0A0B4J1Y9 | Medium | 1.157 | 9 | 1 | 1 | 1 | 11304410 |  |
| AGBL3 | Q8NEM8 | Medium | 1.157 | 1 | 1 | 2 | 1 | 171168928 |  |
| PRKDC | P78527 | Medium | 1.138 | 0 | 1 | 1 | 1 | 7403259 |  |
| ACSL3 | O95573 | Medium | 1.116 | 2 | 1 | 1 | 1 |  |  |
| ZNF75CP | Q92670 | Medium | 1.106 | 2 | 1 | 1 | 1 | 29157282 |  |
| EIF3D | O15371 | Medium | 1.09 | 3 | 1 | 1 | 1 | 7399356.5 |  |
| FANCM | Q8IYD8 | Medium | 1.087 | 0 | 1 | 1 | 1 | 62200144 |  |
| PGM2 | Q96G03 | Medium | 1.086 | 1 | 1 | 1 | 1 | 12238420 |  |
| PSMC3 | P17980 | Medium | 1.077 | 4 | 1 | 1 | 1 | 18736812 |  |
| EPPK1 | P58107 | Medium | 1.076 | 1 | 1 | 1 | 1 | 48024280 |  |
| SCMH1 | Q96GD3 | Medium | 1.064 | 1 | 1 | 1 | 1 | 9799587 |  |
| TTLL11 | Q8NHH1 | Medium | 1.057 | 2 | 1 | 1 | 1 |  |  |
| TSR1 | Q2NL82 | Medium | 1.046 | 2 | 1 | 1 | 1 | 22193350 |  |
| MYH8 | P13535 | Medium | 1.044 | 1 | 1 | 2 | 1 | 296453184 |  |
| GID8 | Q9NWU2 | Medium | 1.012 | 10 | 1 | 1 | 1 | 8930104 |  |
| MRPS26 | Q9BYN8 | Medium | 0.995 | 5 | 1 | 1 | 1 | 66067092 |  |
| DNMT3B | Q9UBC3 | Medium | 0.989 | 2 | 1 | 1 | 1 |  |  |
| SNX31 | Q8N9S9 | Medium | 0.98 | 3 | 1 | 1 | 1 | 47188296 |  |
| INPP4B | O15327 | Medium | 0.96 | 3 | 1 | 1 | 1 | 110083632 |  |
| TAS2R5 | Q9NYW4 | Medium | 0.959 | 3 | 1 | 1 | 1 |  |  |
| PSD4 | Q8NDX1 | Medium | 0.948 | 1 | 1 | 1 | 1 | 15428688 |  |
| SIGLEC16 | A6NMB1 | Medium | 0.934 | 1 | 1 | 1 | 1 |  |  |
| PRDX3 | P30048 | Medium | 0.928 | 5 | 1 | 1 | 1 | 6227934.5 |  |
| GPATCH11 | Q8N954 | Medium | 0.926 | 4 | 1 | 1 | 1 | 2975657.5 |  |
| CHD4 | Q14839 | Medium | 0.908 | 1 | 1 | 1 | 1 | 5001507 |  |
| PSMB2 | P49721 | Medium | 0.889 | 3 | 1 | 1 | 1 | 90648144 |  |
| BRCA2 | P51587 | Medium | 0.886 | 1 | 1 | 1 | 1 |  |  |
| ABCA13 | Q86UQ4 | Medium | 0.885 | 0 | 1 | 1 | 1 |  |  |
| CRYM | Q14894 | Medium | 0.881 | 4 | 1 | 1 | 1 | 160457664 |  |
| MYO1E | Q12965 | Medium | 0.864 | 2 | 1 | 1 | 1 | 103656776 |  |

**Supplementary Table S2**. G-scores of G4 motifs identified in the BRCA1 and RAD51AP1 promotor regions determined using QGRS mapper.

| BRCA1: | | | |
| --- | --- | --- | --- |
| Search Parameters: QGRS Max Length: 30 \| Min G-Group Size: 2 \| Loop size: from 0 to 36 \| Loop search string: | | | |
| QGRS sequences found (overlaps not included) | | |  |
| **Position** | **Length** | **QGRS** | **G-Score** |
| 40 | 27 | **GGCTGGAGTGCAATGGCGCGATCTCGG** | 14 |
| 190 | 16 | **GGTCAGGCTGGTCTGG** | 20 |
| 526 | 26 | **GGGTGAAGGCCTCCTGAGCGCAGGGG** | 8 |
| 718 | 22 | **GGTACAATCAGAGGATGGGAGG** | 13 |
| 764 | 15 | **GGGGCTCTGGATTGG** | 17 |
| 1037 | 11 | **GGGGGTGGAGG** | 21 |
| 1344 | 18 | **GGTCAGTGGCCTGCGGGG** | 16 |
| 1388 | 21 | **GGCAGGGGAAATGCGCTCTGG** | 10 |
| 1517 | 27 | **GGGGTGGTCGTTTTGAGGGACAAGTGG** | 15 |
| 1633 | 21 | **GGCAGAGCTGGCAGCGGACGG** | 16 |
| 1677 | 20 | **GGGGCGGGAAGCTGGTAAGG** | 19 |
| 1707 | 18 | **GGTTAGCTAGGGGTGGGG** | 14 |
| 1742 | 23 | **GGTTTGGAGAAGTTCAAGGGAGG** | 13 |
| 1956 | 29 | **GGCCTTGGCGTCCATTCTGGCCGTGCTGG** | 15 |
| 2014 | 21 | **GGGGGCCCCTCTCTGGGCTGG** | 13 |
| 2049 | 28 | **GGCTCCCTCTGCTTGCGGGGAAGTGTGG** | 7 |
| 2078 | 25 | **GGGAGAGGCGGGTGTGGGAACTGGG** | 39 |
| 2139 | 20 | **GGTGGGCGCGGGCTCAGCGG** | 17 |
| 2172 | 19 | **GGCCCCGGGCAGTCAGGGG** | 15 |
| 2430 | 25 | **GGCTGAGAGGAGTGCAGGCGCCCGG** | 20 |
| 2493 | 26 | **GGCTCCTGAGTCAGATGGGGACTTGG** | 7 |
| RAD51AP1: | | | |
| Search Parameters: QGRS Max Length: 30 \| Min G-Group Size: 2 \| Loop size: from 0 to 36 \| Loop search string: | | | |
| QGRS sequences found (overlaps not included) | | |  |
| **Position** | **Length** | **QGRS** | **G-Score** |
| 1 | 16 | **GGCTCTGGTGGACCGG** | 18 |
| 21 | 27 | **GGAAAAGCGGTGCCTTGGGGAGGGCGG** | 20 |
| 106 | 26 | **GGACGACTGGTAACAGGGGGCGGAGG** | 21 |
| 218 | 23 | **GGACCATGGTGCGGCCTGTGAGG** | 17 |
| 263 | 12 | **GGGGGTGGGAGG** | 20 |
| 284 | 28 | **GGAAAAGACATGAGACTAAGGGGAAAGG** | 4 |
| 325 | 18 | **GGAGGGCAGCGATGGTGG** | 15 |
| 343 | 15 | **GGTTAGGATGGACGG** | 20 |

**Supplementary Table S3**. shRNA sequences, primers and DNA-probes used in this study.

| **shRNA sequences** | **Sequence (5'-3')** | | |
| --- | --- | --- | --- |
| shTRIB3-1 | TGGATGACAACTTAGATACCG | |  |
| shTRIB3-2 | GCCGTGCTCTTCCGCCAGATG | |  |
| shJUN | CGCAAACCTCAGCAACTTCAA | |  |
| shTFAP2C | GCTGAGCTATCTCCTAACTTT | |  |
| shMYC | CAGTTGAAACACAAACTTGAA | |  |
| shDDX5-1 | AGGTGGAAACATACAGAAGAA | |  |
| shDDX5-2 | GCTCCTATTCTGATTGCTACA | |  |
| **Primer used in ChIP assays** | | | |
| **Name** | **Sequence(5'-3')** | |  |
|  | **Forward** | **Reverse** | **Manufacturer** |
| ChIP-JUN-TRIB3-1 | TACACTCCACTGAGCATCCG | GAGGCCTAAGGTCTGCTGAG | General Biol |
| ChIP-JUN-TRIB3-2 | ACAGACATGAGCCAGCTCAC | GATCTCGGCTCACTGCAACC | General Biol |
| ChIP-J-MMP1 | TCTAATGATTGCCTAGTCTATTCATAGC | CTCCAATATCCCAGCTAGGAAGCTCCCTC | General Biol |
| ChIP-GAPDH | TACTAGCGGTTTTACGGGCG | TCGAACAGGAGGAGCAGAGAGCGA | General Biol |
| **Primers used in qRT-PCR analysis** | | |  |
| **Name** | **Sequence(5'-3')** | |  |
|  | **Forward** | **Reverse** | **Manufacturer** |
| qRT-TRIB3 | TTTCCTGACACCTACCTGGC | CGGGACGCTCGGTATCTAAG | General Biol |
| qRT-DDX5 | GCTTCCTGCCATTGTCCACATC | GCAGCTACTTGCTGCACCTGTT | General Biol |
| qRT-JUN | GTGACGGACTGTTCTATGACT | GGGTTACTGTAGCCATAAGGT | General Biol |
| qRT-MYC | CGACGAGACCTTCATCAAAAAC | CTTCTCTGAGACGAGCTTGG | General Biol |
| qRT-TFAP2C | ATTAAGAGGATGCTGGGCTCTG | CACTGTACTGCACACTCACCTT | General Biol |
| qRT-PARP1 | CCAAGCCAGTTCAGGACCTCAT | GGATCTGCCTTTTGCTCAGCTTC | General Biol |
| qRT-BRCA1 | CTGAAGACTGCTCAGGGCTATC | AGGGTAGCTGTTAGAAGGCTGG | General Biol |
| qRT-RAD51 | TCTCTGGCAGTGATGTCCTGGA | TAAAGGGCGGTGGCACTGTCTA | General Biol |
| qRT-RAD51AP1 | CTTCTGGAAGGCAGTGATGGTG | AGAGAAGTCTTCGTCATTATCCTC | General Biol |
| qRT-Actin | GACCTGACTGACTACCTCATGAAGAT | GTCACACTTCATGATGGAGTTGAAGG | General Biol |
| **Probes used in G4 assays** | | | |
| **Name** | **Sequence(5'-3')** | **Modification** | **Manufacturer** |
| Myc G4-Biotin | TGAGGGTGGGTAGGGTGGGTAATTTTT | 5’-biotin | General Biol |
| ss mutMyc | TGAGTCTCTGTAGTGTGTGTAATTTTT | 5’-biotin | General Biol |
| R51AP1-G4-1-Biotin | TGAGGAAAAGCGGTGCCTTGGGGAGGGCGGTAATTTTT | 5’-biotin | General Biol |
| R51AP1-G4-2-Biotin | TGAGGACGACTGGTAACAGGGGGCGGAGGTAATTTTT | 5’-biotin | General Biol |
| BRCA1-G4-1-Biotin | TGAGGGAGAGGCGGGTGTGGGAACTGGGGTAATTTTT | 5’-biotin | General Biol |
| BRCA1-G4-2-Biotin | TGAGGTCAGGCTGGTCTGGTAATTTTT | 5’-biotin | General Biol |
| Stemloop-Biotin | ACGCTTACTTTTGTAAGCGTAATTTTTT | 5’-biotin, | General Biol |
| ds-MYCG4-antisense | AAAAATTACCCACCCTACCCACCCTCA |  | General Biol |
| ds-BRCA1-G4-1 antisense | AAAAATTACCCCAGTTCCCACACCCGCCTCTCCCTCA |  | General Biol |
| ds-RAD51AP1-G4-1 antisense | AAAAATTACCGCCCTCCCCAAGGCACCGCTTTTCCTCA |  | General Biol |
| Myc G4-Pu28 | TGGGGAGGGTGGGGAGGGTGGGGAAGGT | 5’-FAM, 3’-BHQ-1 | General Biol |

**Supplementary Table S4**. Reagents and Resources used in this study.

| **Reagent or Resource** | **Source** | **Identifier** |
| --- | --- | --- |
| **Cell Lines** | | |
| HEK-293T | Anwei-sci Cell Center | Cat# AW-CH0004; RRID:CVCL_0063 |
| HepG2 | Anwei-sci Cell Center | Cat# AW-CH0092; RRID:CVCL_0027 |
| Huh7 | Anwei-sci Cell Center | Cat# AW-CH0160; RRID:CVCL_0336 |
| **Antibodies** | **Source** | **Identifier** |
| Phospho-Histone H2A.X (Ser139) (D7T2V) Mouse mAb | Cell Signaling Technology | Cat# 80312; RRID:AB_2799949 |
| Recombinant Anti-TRIB3 antibody[EPR3151Y] | Abcam | Cat# ab75846; RRID:AB_1310768 |
| Anti-COP1 antibody[1E4] | Abcam | Cat# ab56400; RRID:AB_2178761 |
| GAPDH Monocolonal antibody(1E6D9) | Proteintech | Cat# 60004-1-Ig; RRID:AB_2107436 |
| Beta Actin Monocolonal antibody(2D4H5) | Proteintech | Cat# 66009-1-Ig; RRID:AB_2687938 |
| HSP90 Polycolonal antibody | Proteintech | Cat# 13171-1-AP; RRID:AB_2120924 |
| VDAC1/2 Polyclonal antibody | Proteintech | Cat# 10866-1-AP; RRID:AB_2257153 |
| PARP1 Polyclonal antibody | Proteintech | Cat# 13371-1-AP; RRID:AB_2160459 |
| c-Jun (60A8) Rabbit mAb | Cell Signaling Technology | Cat# 9165; RRID:AB_2130165 |
| DYKDDDDK tag Monoclonal antibody(8H6A10) | Proteintech | Cat# 66008-4-Ig; RRID:AB_2918475 |
| Rabbit anti HA-Tag pAb | Abclonal | Cat# AE036; RRID:AB_2771924 |
| DDX5 Monoclonal antibody(2D3D2) | Proteintech | Cat# 67025-1-Ig; RRID:AB_2882340 |
| hnRNP H2 Antibody | Novus biologicals | Cat# NBP1-89816; RRID:AB_11028969 |
| TCP1 Polyclonal antibody | Proteintech | Cat# 10320-1-AP; RRID:AB_10694136 |
| SSBP1 Polyclonal antibody | Proteintech | Cat# 12212-1-AP; RRID:AB_2195320 |
| Histone H3 Polyclonal antibody | Proteintech | Cat# 17168-1-AP; RRID:AB_2716755 |
| c-Myc (D84C12) Rabbit mAb | Cell Signaling Technology | Cat# 5605; RRID:AB_1903938 |
| BRCA1 Polyclonal antibody | Proteintech | Cat# 22362-1-AP; RRID:AB_2879090 |
| RAD51 Polyclonal antibody | Proteintech | Cat# 14961-1-AP; RRID:AB_2177083 |
| RAD51AP1 Polyclonal antibody | Proteintech | Cat# 11255-1-AP; RRID:AB_2300786 |
| Rabbit anti mouse IgG (H&L) | Abcam | Cat# ab46540; RRID:AB_2614925 |
| Donkey anti rabbit IgG (H&L) | Abcam | Cat# ab6701; RRID:AB_956011 |
| Goat anti rabbit IgG (H&L)-AF 488 conjugate | BOSTER Biological Technology | Cat# BA1127; RRID:AB_3713475 |
| Goat anti rabbit IgG (H&L)-AF 594 conjugate | BOSTER Biological Technology | Cat# BA1141; RRID:AB_2941989 |
| Goat anti mouse IgG (H&L)-HRP conjugate | Zenbio | Cat# 511103; RRID:AB_2893489 |
| Goat anti rabbit IgG (H&L)-HRP conjugate | Zenbio | Cat# 511203; RRID:AB_2927753 |
| Normal Mouse IgG | Sigma aldrich | Cat# 12-371; RRID:AB_145840 |
| Normal Rabbit IgG | Sigma aldrich | Cat# 12-370; RRID:AB_145841 |
| **Plasmids** |  |  |
| pLKO.1-puro | This paper |  |
| pLKO.1-shTRIB3-1 | This paper |  |
| pLKO.1-shTRIB3-2 | This paper |  |
| pLKO.1-shDDX5-1 | This paper |  |
| pLKO.1-shDDX5-2 | This paper |  |
| pLKO.1-shJUN | This paper |  |
| pLKO.1-shMYC | This paper |  |
| pLKO.1-shTFAP2C | This paper |  |
| FH1t(UTG)-shTRIB3-1 | This paper |  |
| pGL3-basic | This paper |  |
| pGL3-TRIB3-JUN-P1 | This paper |  |
| pGL3-TRIB3-JUN-P2 | This paper |  |
| Renilla | This paper |  |
| pCAG4-RTR2 (RRE) | This paper |  |
| pKGP1 (REV) | This paper |  |
| CAGG5-VSVG | This paper |  |
| pCDH-CMV-MCS-EF1-Puro | This paper |  |
| pcDH-CMV-TRIB3-FL | This paper |  |
| pCDH-CMV-TRIB3-3×FLAG | This paper |  |
| pCDH-CMV-DDX5-3×HA | This paper |  |
| pCDH-CMV-TRIB3-myc-puro | This paper |  |
| pcDNA3.1(+) | This paper |  |
| pcDNA3.1-TRIB3-3×FLAG | This paper |  |
| pcDNA3.1-TRIB3-Δ1-117-3×FLAG | This paper |  |
| pcDNA3.1-TRIB3-Δ118-314-3×FLAG | This paper |  |
| pcDNA3.1-TRIB3-Δ315-358-3×FLAG | This paper |  |
| pcDNA3.1-DDX5-HA | This paper |  |
| pcDNA3.1-DDX5-Δ1-123-HA | This paper |  |
| pcDNA3.1-DDX5-Δ124-300-HA | This paper |  |
| pcDNA3.1-DDX5-Δ301-475-HA | This paper |  |
| pcDNA3.1-DDX5-Δ476-614-HA | This paper |  |
| pDRGFP | This paper |  |
| pCBASceI | This paper |  |
| Lenti-EF1α-mCherry-Puro | This paper |  |
| **Chemicals, peptides, and recombinant proteins** | **Source** | **Identifier** |
| BG4-Flag Recombinant Protein | A kindly gift from professor Lianxin Liu, USTC, China |  |
| DDX5 Human Recombinant Protein | Origene | Cat# TP300371 |
| TRIB3 (1-358, His-tag) Human Protein | Origene | Cat# AR51742PU-S |
| Pyridostatin | Selleck | Cat# E8172; CAS: 1085412-37-8 |
| PhenDC3 | Selleck | Cat# E1070; CAS: 929895-45-4 |
| Etoposide | Selleck | Cat# S1225; CAS: 33419-42-0 |
| Doxycycline Hyclate | Sigma | Cat# D9891; CAS: 24390-14-5 |
| Doxycycline Hyclate | TargetMol | Cat# T1687L; CAS: 24390-14-6 |
| Tris-Saturated Phenol | Sangon | Cat# A504193-0100 |
| **Critical commercial assays** | **Source** | **Identifier** |
| DMEM (High Glucose) | Thermo Fischer | Cat# 12800082 |
| DMEM (High Glucose, no glutamine) | Thermo Fischer | Cat# 11960044 |
| Opti-MEM reduced medium | Thermo Fischer | Cat# 31985062 |
| GlutaMAX supplement | Thermo Fischer | Cat# 35050061 |
| Sodium Pyruvate(100mM) | Thermo Fischer | Cat# 11360070 |
| 0.25% Trypsin-EDTA | Thermo Fischer | Cat# 25200056 |
| Fetal Bovine Serum | LONSERA | Cat# S711-001S |
| MycoBlue Mycoplasma Detector | Vazyme | Cat# D101 |
| Lipofectamine 2000 Reagent | Thermo Fischer | Cat# 11668019 |
| Cell Counting Kit-8 | TargetMol | Cat# C0005 |
| PrimSTAR MAX DNA Polymerase | TAKARA | Cat# R045 |
| ClonExpress Ultra One Step Cloning | Vazyme | Cat# C115 |
| Endo Free Plasmid mini Kit II | Omega Bio-tek | Cat# D6950-02 |
| DL 10,000 DNA Ladder | TAKARA | Cat# 3584A |
| DL 2,000 DNA Ladder | TAKARA | Cat# 3427A |
| 6×DNA Loading Buffer | TAKARA | Cat# 9156 |
| Reactive Oxygen Species Assay Kit | Beyotime | Cat# S0033 |
| GSH and GSSG Assay Kit | Beyotime | Cat# S0053 |
| Dual-Luciferase® Assay System | Promega | Cat# E1910 |
| ChIP assay Kit | Beyotime | Cat# P2078 |
| Comet assay kit | R&D systems | Cat# 4250-050-K |
| NE-PER Nuclear and Cytoplasmic Extraction Reagents | Thermo Fischer | Cat# 78833 |
| Mitochondria Isolation Kit for Mammalian Cells | Thermo Fischer | Cat# 89874 |
| Pierce™ Rapid Gold BCA Assay | Thermo Fischer | Cat# A53226 |
| SuperSignal™ West Pico PLUS Chemiluminescence Substrate | Thermo Fischer | Cat# 34580 |
| SYBR Green Premix SupTaq HS qPCR Kit（Low Rox Plus） | AGBio | Cat# AG11762 |
| HiScript III All-in-one RT SuperMix Perfect for qPCR | Vazyme | Cat# R333 |
| Hyperactive Universal CUT&Tag Assay Kit for Illumina | Vazyme | Cat# TD903 |
| TruePrp Index Kit V2 for Illumina | Vazyme | Cat# TD202 |
| Duolink® In Situ PLA® Probe Anti-Mouse PLUS | Sigma-Aldrich | Cat# DUO92001 |
| Duolink® In Situ PLA® Probe Anti-Rabbit MINUS | Sigma-Aldrich | Cat# DUO92005 |
| Duolink® In Situ Detection Reagents Green | Sigma-Aldrich | Cat# DUO92014 |
| **Software** |  |  |
| GraphPad Prism (RRID:SCR_002798) |  |  |
| Fiji (RRID:SCR_002285) |  |  |
| SAMTOOLS (RRID:SCR_002105) |  |  |
| STRING (RRID:SCR_005223) |  |  |
| Integrative Genomics Viewer (RRID:SCR_011793) |  |  |
| STAR (RRID:SCR_004463) |  |  |
| **Oligonucleotides** |  |  |
| shRNA sequences | Table S3 |  |
| DNA probes | Table S3 |  |
| qPCR primers | Table S3 |  |
